# Blocking STAT3/5 through direct or upstream kinase targeting in leukemic cutaneous T‐cell lymphoma

**DOI:** 10.15252/emmm.202115200

**Published:** 2022-11-07

**Authors:** Helena Sorger, Saptaswa Dey, Pablo Augusto Vieyra‐Garcia, Daniel Pölöske, Andrea R Teufelberger, Elvin D de Araujo, Abootaleb Sedighi, Ricarda Graf, Benjamin Spiegl, Isaac Lazzeri, Till Braun, Ines Garces de los Fayos Alonso, Michaela Schlederer, Gerald Timelthaler, Petra Kodajova, Christine Pirker, Marta Surbek, Michael Machtinger, Thomas Graier, Isabella Perchthaler, Yi Pan, Regina Fink‐Puches, Lorenzo Cerroni, Jennifer Ober, Moritz Otte, Jana D Albrecht, Gary Tin, Ayah Abdeldayem, Pimyupa Manaswiyoungkul, Olasunkanmi O Olaoye, Martin L Metzelder, Anna Orlova, Walter Berger, Marion Wobser, Jan P Nicolay, Fiona André, Van Anh Nguyen, Heidi A Neubauer, Roman Fleck, Olaf Merkel, Marco Herling, Ellen Heitzer, Patrick T Gunning, Lukas Kenner, Richard Moriggl, Peter Wolf

**Affiliations:** ^1^ Unit of Functional Cancer Genomics, Institute of Animal Breeding and Genetics University of Veterinary Medicine Vienna Austria; ^2^ Department of Pediatric and Adolescent Surgery, Vienna General Hospital Medical University of Vienna Vienna Austria; ^3^ Department of Dermatology and Venereology Medical University of Graz Graz Austria; ^4^ Department of Pathology Medical University of Vienna Vienna Austria; ^5^ Department of Chemical and Physical Sciences University of Toronto Mississauga Mississauga ON Canada; ^6^ Centre for Medicinal Chemistry University of Toronto Mississauga Mississauga ON Canada; ^7^ Diagnostic & Research Center for Molecular Bio‐Medicine, Institute of Human Genetics Medical University of Graz Graz Austria; ^8^ Department of Medicine I CIO‐ABCD, CECAD and CMMC Cologne University Cologne Germany; ^9^ Unit of Laboratory Animal Pathology University of Veterinary Medicine Vienna Vienna Austria; ^10^ Centre for Cancer Research Medical University of Vienna Vienna Austria; ^11^ Comprehensive Cancer Center Medical University of Vienna Vienna Austria; ^12^ Core Facility Flow Cytometry, Center for Medical Research (ZMF) Medical University of Graz Graz Austria; ^13^ Department of Dermatology University Hospital Mannheim Mannheim Germany; ^14^ Department of Dermatology University Hospital Wuerzburg Wuerzburg Germany; ^15^ University Clinic for Dermatology, Venereology and Allergology Innsbruck Medical University of Innsbruck Innsbruck Austria; ^16^ Janpix, a Centessa Company London UK; ^17^ Department of Hematology, Cellular Therapy, and Hemostaseology University of Leipzig Leipzig Germany; ^18^ Christian Doppler Laboratory for Applied Metabolomics (CDL‐AM), Division of Nuclear Medicine Medical University of Vienna Vienna Austria; ^19^ CBmed GmbH Center for Biomarker Research in Medicine Graz Austria; ^20^ BioTechMed Graz Graz Austria

**Keywords:** lymphoma, STAT3, STAT5, targeting, T‐cell, Cancer, Chromatin, Transcription & Genomics

## Abstract

Leukemic cutaneous T‐cell lymphomas (L‐CTCL) are lymphoproliferative disorders of skin‐homing mature T‐cells causing severe symptoms and high mortality through chronic inflammation, tissue destruction, and serious infections. Despite numerous genomic sequencing efforts, recurrent driver mutations have not been identified, but chromosomal losses and gains are frequent and dominant. We integrated genomic landscape analyses with innovative pharmacologic interference studies to identify key vulnerable nodes in L‐CTCL. We detected copy number gains of loci containing the *STAT3/5* oncogenes in 74% (*n* = 17/23) of L‐CTCL, which correlated with the increased clonal T‐cell count in the blood. Dual inhibition of STAT3/5 using small‐molecule degraders and multi‐kinase blockers abolished L‐CTCL cell growth *in vitro* and *ex vivo*, whereby PAK kinase inhibition was specifically selective for L‐CTCL patient cells carrying *STAT3/5* gains. Importantly, the PAK inhibitor FRAx597 demonstrated encouraging anti‐leukemic activity *in vivo* by inhibiting tumor growth and disease dissemination in intradermally xenografted mice. We conclude that STAT3/5 and PAK kinase interaction represents a new therapeutic node to be further explored in L‐CTCL.

## Graphical abstract

L‐CTCL remains a poorly understood rare T‐cell cancer entity. Frequently amplified *STAT3/5* oncogenes and related kinase action relevant for T‐cell survival were identified as vulnerable nodes for targeting, thereby improving the prospects of a translatable targeted drug for L‐CTCL.

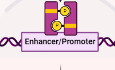

**The paper explained**

**Problem**

Leukemic forms of cutaneous T‐cell lymphomas (L‐CTCL) are lymphoproliferative disorders of skin‐homing mature T‐cells, for which oncogenic drivers and key chromosomal rearrangements relating to disease progression remain elusive. Current therapies lead to abysmal and often short‐lived responses. Given the struggle that L‐CTCL patients face due to high symptomatic burden, inefficient treatments, and considerable mortality, we explored novel targeted therapy options.

**Results**

*STAT3/5* copy number gains were present in 74% (*n* = 17/23) of L‐CTCL patients and correlated with disease clonality. Targeting the resulting STAT3/5 overexpression and hyperactivation using novel specific small‐molecule degraders and (multi)kinase blockers resulted in cell death, whereby PAK kinase inhibition was specifically effective in patient cells carrying *STAT3/5* gains. Furthermore, PAK kinase inhibition induced significant decreases in tumor size and CTCL cell dissemination in an intradermal xenograft mouse model.

**Impact**

The highly frequent *STAT3/5* copy number gains in L‐CTCL, which result in STAT3/5 overexpression and hyperactivation, might serve as a novel marker for treatment guidance and disease monitoring. Further development of the characterized STAT3/5 degraders and improvement of lead structure IQDMA/PAK kinase inhibitors should be continued in the search for curative targeted drugs.

## Introduction

Cutaneous T‐cell lymphoma (CTCL) represents a heterogeneous group of non‐Hodgkin lymphomas characterized by the infiltration and expansion of neoplastic mature T‐cells, primarily in the skin. The two most common subtypes of CTCL are the skin‐confined mycosis fungoides (MF) and the leukemic Sézary syndrome (SS), together accounting for over 50% of cutaneous lymphoma patients (Willemze *et al*, 2005, 2019). Most early‐stage MF patients, who characteristically present with cutaneous patches and plaques, are generally successfully treated with conventional skin‐directed therapies, like topical steroids, photo(chemo)therapy, or single‐agent cytotoxic chemotherapeutic agents, such as methotrexate (Agar *et al*, 2010). However, a significant fraction of these patients (~25%) will progress into an advanced disease with a median overall survival (OS) < 4 years, and for those patients who show involvement of blood, lymph nodes, and visceral organs, OS drops to ~13 months (Scarisbrick *et al*, 2014; Murray *et al*, 2019). In contrast, patients with primary (*de novo*) SS present upfront with leukemic disease (> 1,000 Sézary cells/μl blood) and generalized erythroderma. Responses to available therapies in SS are often not profound, and the prognosis is poor with a 5‐year OS rate of only 36% (Agar *et al*, 2010; Willemze *et al*, 2019).

Currently, FDA‐approved agents for CTCL include histone deacetylase inhibitors (HDACi), such as vorinostat or romidepsin, and the monoclonal antibody mogamulizumab or antibody–drug conjugates, like brentuximab vedotin or denileukin diftitox (Devata & Wilcox, 2016). However, overall response rates to these agents are in the range 30–35% (Devata & Wilcox, 2016). Therefore, there is an unmet clinical need for more effective therapies, especially for advanced and leukemic CTCL (L‐CTCL). This, in turn, calls for a better understanding of CTCL pathogenesis and its actionable vulnerabilities.

Comprehensive genomic analyses on large CTCL patient cohorts failed to identify recurrent driver mutations, but chromosomal instability was revealed as a key disease feature associated with adverse clinical outcomes (Karenko *et al*, 2007; van Doorn *et al*, 2009; Kiel *et al*, 2015). Specifically, chromosome 17 copy number alterations (CNA) were frequently detected (Caprini *et al*, 2009, 2018; van Doorn *et al*, 2009; Lin *et al*, 2012; Choi *et al*, 2015; da Silva Almeida *et al*, 2015; Kiel *et al*, 2015; Wang *et al*, 2015; Prasad *et al*, 2016; Woollard *et al*, 2016; Fanok *et al*, 2018). They present either as chromosome 17p deletions, resulting in the loss of *TP53*, and/or duplications of chromosomal segments, or the entire arm of chromosome 17q, where *STAT3* and *STAT5A/B* genes are located. Also frequent are chromosome 8q gene amplifications driving high *c‐MYC* oncoprotein expression (Karenko *et al*, 2007; van Doorn *et al*, 2009; Laharanne *et al*, 2010; Salgado *et al*, 2010; Kiel *et al*, 2015; Fanok *et al*, 2018).

STAT3/5 hyperactivation in CTCL was suggested to contribute to disease progression (Eriksen *et al*, 2001; Netchiporouk *et al*, 2014). In particular, increased/constitutive activation of STAT3/5 in CTCL was proposed to be linked to inadequate responses to HDACi, such as vorinostat (Eriksen *et al*, 2001; Fantin *et al*, 2008). These findings suggest that enhanced oncogenic STAT3/5 activation could be an important disease‐driving event. However, none of the previous studies addressed the efficacy of direct STAT3/5 targeting. Furthermore, no targetable kinases upstream of oncogenic STAT3/5 action, other than JAK kinases, were identified.

Here, we integrated shallow whole‐genome (sWGS) and whole‐exome sequencing (WES) on a cohort of SS and advanced leukemic MF patients to reveal in‐depth the genomic landscape of L‐CTCL. The most frequent genomic aberrations were copy number gains of *MYC*, loss of *TP53*, and losses of *SOCS1* and/or *STAT1* co‐occurring with copy number gains of *STAT3*/5; the latter of which were overall present in 74% of L‐CTCL patients and resulted in increased expression and hyperactivation of STAT3/5. Since the observed *STAT3/5* copy number gains in our cohort correlated with disease severity, we hypothesized that targeting STAT3/5 could be beneficial to eliminate L‐CTCL cells. By utilizing human cell models that have a genetic fingerprint representative of L‐CTCL, we comprehensively investigated the therapeutic efficacy of STAT3/5 targeting by using dual STAT3/5 degraders and kinase inhibitors. The small‐molecule STAT3/5 degraders, IQDMA, a multi‐kinase inhibitor, and PAK kinase inhibitor exhibited remarkable potency in abolishing L‐CTCL cell growth *in vitro* and *ex vivo*, whereby PAK kinase inhibition was specifically selective for primary L‐CTCL cells with *STAT3/5* gains. Furthermore, the PAK kinase inhibitor significantly reduced tumor growth and disease dissemination *in vivo*. In summary, *STAT3/5* copy number gain and PAK kinase interaction can be exploited as vulnerable nodes in L‐CTCL.

## Results

### *STAT3* */5* copy number gains are frequent in L‐CTCL and co‐occur with copy number loss of *STAT1* / *SOCS1*

A total of 35 L‐CTCL patients were enrolled in the study, specifically SS and leukemic advanced MF (L‐AMF) patients, with the requirement of being erythrodermic and having more than 1,000 Sézary cells/mm^3^ in peripheral blood (Table EV1). A distinct malignant T‐cell clone in the blood was detected in 24 of 35 patients by TCRVβ immunophenotyping (Appendix Fig S1 and Table EV1). Overall, the malignant clone size in the blood ranged from 11.5 to 95.7%/CD3^+^ cells. The histology of skin biopsies was consistent with the diagnosis of SS or L‐AMF (Table EV2).

**Figure 1:**
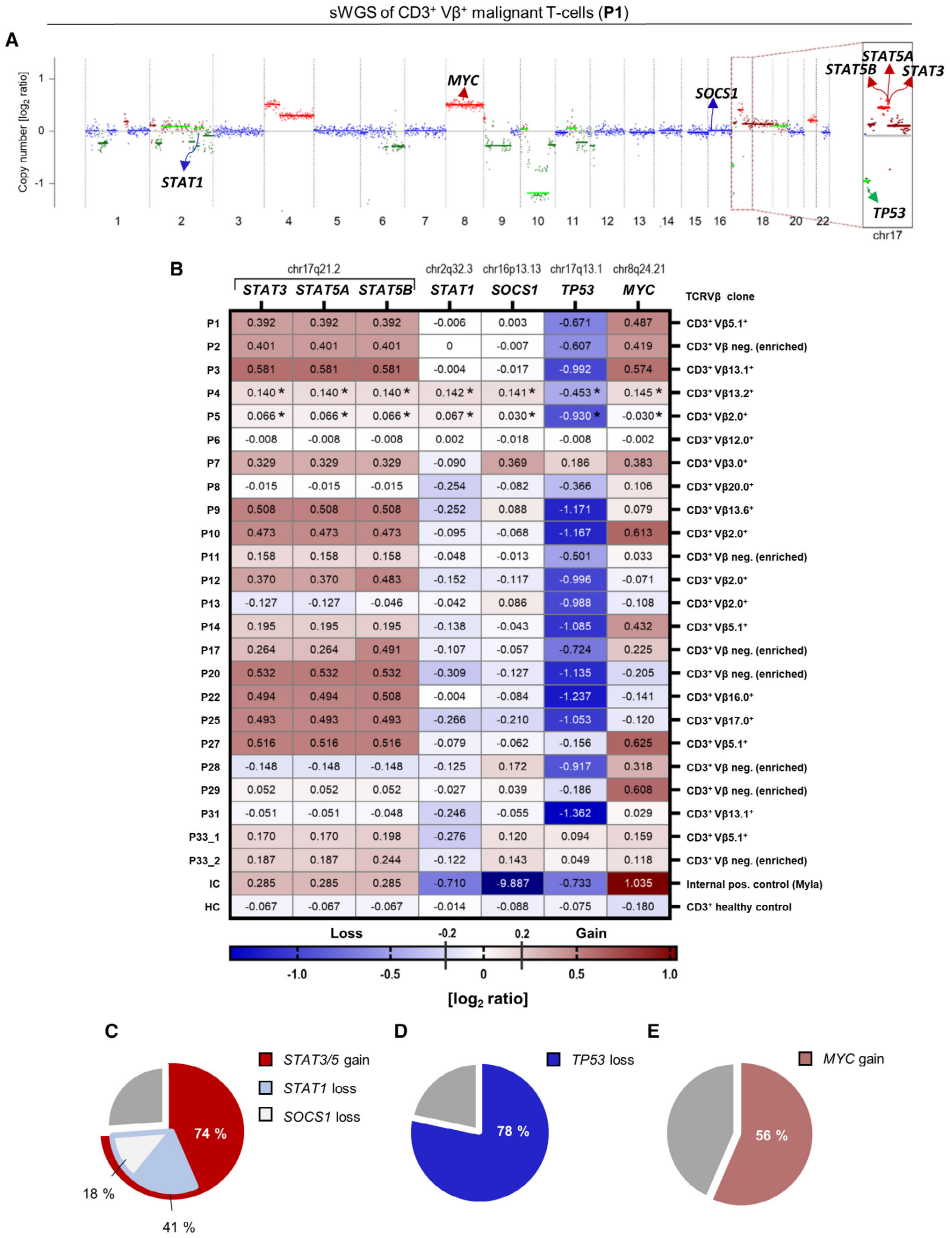
L‐CTCL patients present with *STAT3/5* and *MYC* copy number gains, as well as *TP53*, *STAT1*, and *SOCS1* copy number loss AsWGS performed on sorted CD3^+^/Vβ^+^ malignant T‐cells isolated from the blood of P1, shown as a representative example. Copy number profiles and ploidy estimates were calculated using ichorCNA. Blue represents balanced genomic regions, green indicates loss/deletion, and red indicates gain/amplification of regions on chromosome 17. Darker color shades indicate higher log_2_ ratios. Copy number profiles from all six patients (P1–P6) are shown in Appendix Fig S2.BHeatmap of extracted CNA [log_2_ ratios] of STAT genes on chr17 (i.e., *STAT3*, *STAT5A*, and *STAT5B*), and other relevant tumor suppressors (i.e., *STAT1*, *SOCS1*, and *TP53*) and oncogenes (*MYC*). Red indicates gain/amplification and blue indicates loss/deletion. The balance threshold was set to ±0.1. P33 presented with two malignant clones in the blood and both were sequenced (P33_1 (Clone 1) and P33_2 (Clone 2)). *Tetraploid malignant clone.C–EPie charts representing the identified genetic alterations in the patient cohort based on the CNA log_2_ ratios. (C) *STA3/5* copy number gain (*n* = 17/23), *STAT1* copy number loss (*n* = 7/17), *SOCS1* loss (*n* = 3/17), (D) *TP53* loss (*n* = 18/23), and (E) *MYC* gain (*n* = 13/23).

To elucidate the genetic landscape of driver mutations in L‐CTCL, we performed WES on sorted CD3^+^/Vβ^+^ malignant T‐cells and donor‐matched sorted CD19^+^ healthy B‐cells from six patients (P1–P6). Algorithms included filtering for variants with variant allele frequencies (VAF) > 0.2 for single‐nucleotide variants (SNVs) and > 0.05 for indels, while excluding synonymous and intronic variants (except for putative splice mutations). Using these stringent filter criteria, an average of 69 somatic mutations (range 19–120) were identified in the T‐cell fraction per patient sample, with a mean of five mutations (range 0–7) located in a Catalog of Somatic Mutations in Cancer (COSMIC) cancer census gene (Dataset EV1. Whole‐exome sequencing). Additionally, variants in genes, which according to the COSMIC database are found mutated in CTCL at a low frequency, were observed in P1 (*SMARCB1*), P2 (*FAT4*), P4 (*PLCG1*), and P5 (*TP53*, *FAT4*; Dataset EV1. Whole‐exome sequencing, analysis criteria). In contrast to the low frequency of somatic mutations in cancer driver genes, sWGS of patient DNA from the six patients (P1–P6) revealed a high frequency of somatic copy number alterations (CNA; Fig 1A and Appendix Fig S2). Most of these CNA were also reported in larger cohorts of CTCL patients, such as gains of 8q, harboring the *MYC* locus, or deletions of the *TP53* locus on 17p (Fig 1A and Appendix Fig S2; Karenko *et al*, 2007; van Doorn *et al*, 2009; Laharanne *et al*, 2010; Salgado *et al*, 2010; Kiel *et al*, 2015; McGirt *et al*, 2015). Notably, four patients (66%; P2–P5) showed a gain of 17q, and one patient (16%; P1) had a high‐level amplification of the 17q11.2–21.31 region, which includes the *STAT3* and *STAT5A/B* genes (Fig 1A and Appendix Fig S2).

**Figure EV1:**
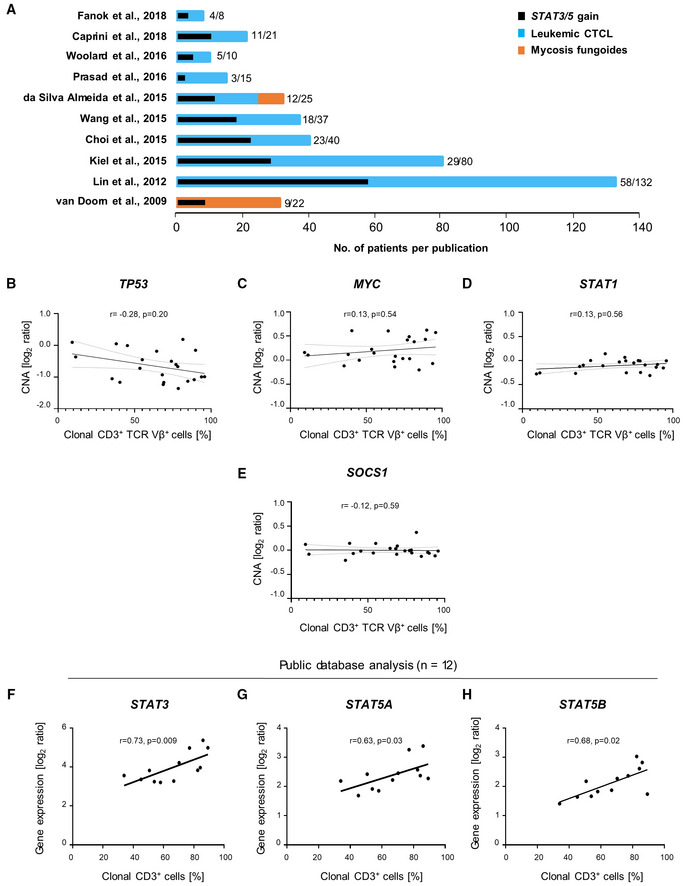
*STAT3/5* expression correlates with disease clonality AGraphical summary of the literature analysis on genomic CTCL studies performed in the last 10 years including larger cohorts of patients, as described in Table EV3. The number of patients carrying 17q gain, specifically of the region containing the *STAT3/5* genes (17q11.2‐17q21.31) is written and depicted in black, in comparison to the total number of patients included and depicted in blue or orange. Blue color depicts Sezary syndrome patients, whereas orange depicts mycosis fungoides patients.B–ESpearman correlation analysis using CNA log_2_ ratios and the percentage of clonal CD3^+^ cells detected in patients with (B) *TP53*, (C) *MYC*, (D) *STAT1*, and (E) *SOCS1*.F–HSpearman correlation analysis on expression data extracted from the Oncomine™ Platform as published in Caprini *et al* (2009). Patients with 17q (*STAT3/5*) gains were selected and the percentage of clonal CD3^+^ cells was correlated with the respective *STAT3/5* gene expression for the (F) *STAT3*_208991 reporter, (G) *STAT5A*_203010 reporter, and (H) *STAT5B*_212550 reporter. Source data are available online for this figure.

Expanding on our findings and investigating the frequency of the identified genetic aberrations on a larger cohort, we performed sWGS of sorted CD3^+^/Vβ^+^ malignant T‐cells from additional 17 patients, which passed the required quality control (Fig 1B and Table EV1). Focusing specifically on above‐described regions and extracting the CNA log_2_ ratios from the sequencing data, we revealed that 74% of samples (*n* = 17/23) carry *STAT3/5* copy number gains (range 0.170–0.581 log_2_ ratios; including tetraploid samples from P4 and P5; Fig 1B and C, and Dataset EV2. CNA log_2_ ratios). Also confirming previous findings, 78% of samples (*n* = 18/23) carried copy number losses of *TP53* (range −0.501 to −1.362 log_2_ ratios; Fig 1B and D, and Dataset EV2. CNA log_2_ ratios), while 56% (*n* = 13/23) had copy number gains of *MYC* (range 0.118–0.625 log_2_ ratios; Fig 1B and E, and Dataset EV2. CNA log_2_ ratios). Importantly, we noted also other genetic changes in tumor suppressors co‐occurring with *STAT3/5* gains, namely *STAT1* copy number losses in 41% (*n* = 7/17; range −0.122 to −0.309 log_2_ ratios) and *SOCS1* losses in 18% of samples (*n* = 3/17; range −0.117 to −0.210; Fig 1B and C, and Dataset EV2. CNA log_2_ ratios). Finally, we compared our findings, particularly the 17q gains, to recent genome‐wide studies covering more CTCL patients (Fig EV1A). A gain of 17q, specifically of the region encompassing *STAT3/*5, was present in ~50% of patients with the advanced and leukemic disease (Fig EV1A and Table EV3). Overall, we conclude that copy number gains of *STAT3/5* and *MYC*, as well as copy number losses of *TP53*, are the most frequent genomic abnormalities in L‐CTCL. Moreover, we are the first to show that copy number gain of *STAT3/5* co‐occurs with loss of *STAT1* and *SOCS1*, indicating that lost growth inhibitory function and lost negative feedback control play an important role in L‐CTCL disease pathogenesis.

### Elevated *STAT3/5* copy number and expression levels correlate with increased clonal proliferation in L‐CTCL

**Figure 2:**
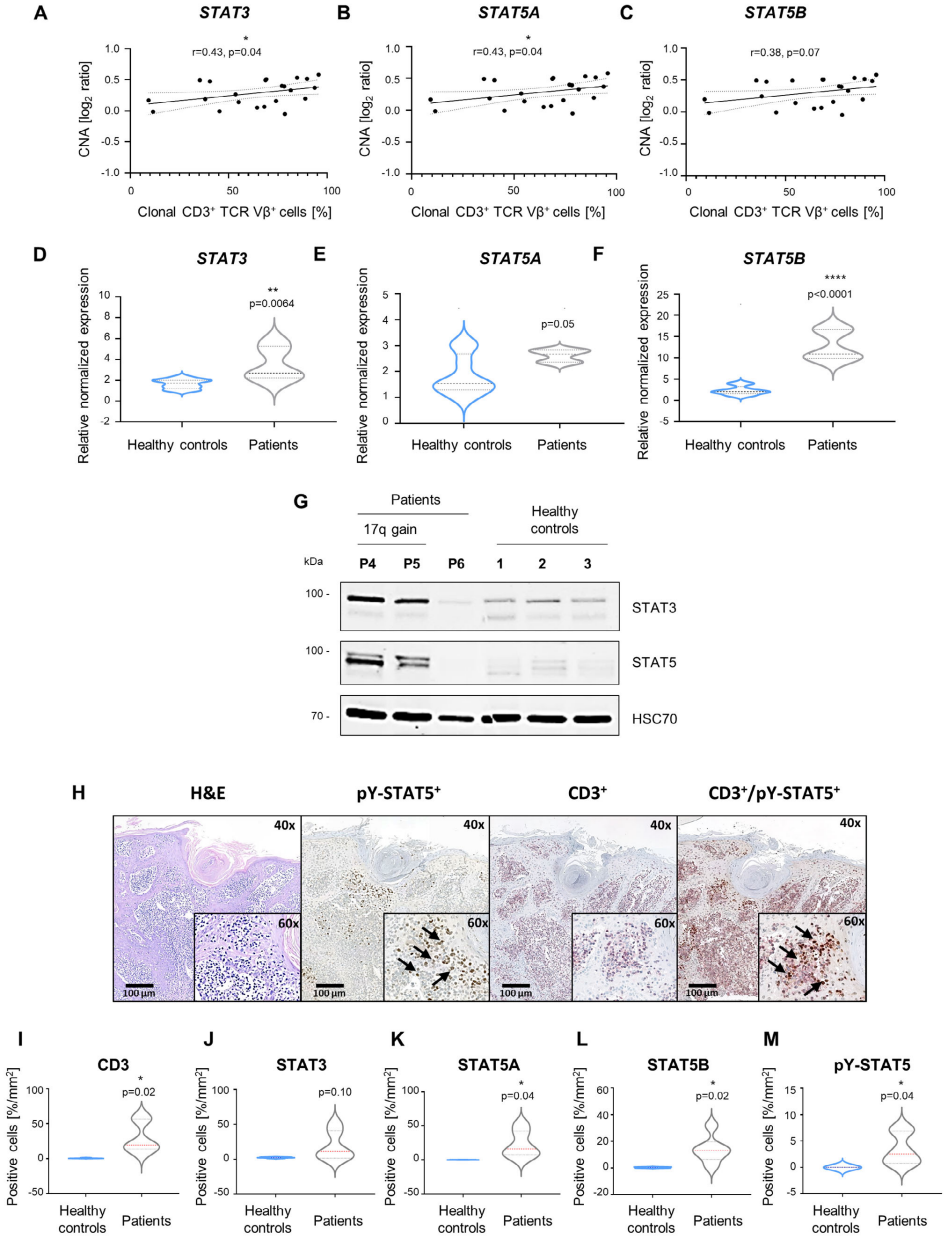
*STAT3/5* copy number gain correlates with disease clonality and results in STAT3/5 overexpression and hyperactivation A–CSpearman correlation analysis using CNA log_2_ ratios and the percentage of clonal CD3^+^ cells detected in patients for (A) *STAT3*, (B) *STAT5A*, and (C) *STAT5B*.D–FRT–qPCR expression data of (C) *STAT3*, (D) *STAT5A*, and (E) *STAT5B* genes from whole PBMCs isolated from patient blood (*n* = 5) or healthy controls (*n* = 9). *GAPDH* expression was used for normalization. Statistical significance was calculated using a two‐tailed unpaired *t*‐test. *P*‐value: < 0.01 (**) and < 0.0001 (****). One experiment was performed in technical triplicates. The bold dashed line in the middle of the violin plot denotes the median value, while the thin dotted lines denote the interquartile range.GImmunoblotting for total STAT3/5 protein isolated from the respective whole PBMCs from patient blood (*n* = 3) or healthy controls (*n* = 3). HSC70 was used as loading control. One experiment was performed.HRepresentative image of H&E, CD3, pY‐STAT5 single staining, and CD3/pY‐STAT5 double staining on skin biopsy tissue from P3 (*n* = 1), which demonstrates infiltration of CD3^+^ pY‐STAT5^+^ (13.45%) cells into the dermal and subcutaneous layer of the skin. Arrows indicate pY‐STAT5^+^ single and CD3^+^ pY‐STAT5^+^ cells.I–MQuantification of IHC staining from skin‐biopsy sections of L‐CTCL patients (*n* = 6) and healthy individuals (*n* = 5) depicted in Appendix Fig S3. As the number of STAT5A/B‐positive cells was negligible (less than 0.03/per mm^2^) in healthy skin biopsy sections, healthy control samples were considered negative for pY‐STAT5, and levels were set to 0. Statistical significance was calculated using an unpaired Welch's *t*‐test. *P*‐value: < 0.05 (*). The bold dashed line in the middle of the violin plot denotes the median value, while the thin dotted lines denote the interquartile range. Source data are available online for this figure.

To investigate the biological significance of the most commonly observed genetic aberrations for CTCL pathogenesis, namely *STAT3/5*, *STAT1*, *SOCS1*, *TP53*, and *MYC*, we correlated the detected CNA log_2_ ratios to the percentage of clonal CD3^+^ malignant cells detected in peripheral blood (Figs 2A–C and EV1B–E, and Table EV1). We observed a significantly positive correlation for *STAT3* and *STAT5A* (*STAT3*: Spearman *r* = 0.43, *P* = 0.04; *STAT5A*: Spearman *r* = 0.43, *P* = 0.04) and a positive trend for *STAT5B* (*STAT5B*: Spearman *r* = 0.38, *P* = 0.07; Fig 2A–C). In contrast, *TP53* showed a trend for a negative correlation, whereas *MYC*, *STAT1*, and *SOCS1* did not correlate with disease clonality (*TP53*: Spearman *r* = −0.28, *P* = 0.20; *MYC*: Spearman *r* = 0.13, *P* = 0.54, *STAT1*: Spearman *r* = 0.13, *P* = 0.56; *SOCS1*: Spearman *r* = −0.12, *P* = 0.59), indicating that *STAT3/5* copy number gains correlate with disease severity (Fig EV1B–E).

Furthermore, to verify whether *STAT3/5* copy number gains are associated with elevated expression of *STAT3* and *STAT5A/B*, we performed RT–qPCR on mRNA isolated from peripheral blood mononuclear cells (PBMCs) of L‐CTCL patients (*n* = 5) and healthy donors (*n* = 9). Expression of *STAT3* and *STAT5B* was significantly increased in L‐CTCL samples compared to healthy controls (Fig 2D–F). Extracted expression and clinical data from a previously published study corroborated these results (Caprini *et al*, 2009). It showed a significant positive correlation between *STAT3/5* gene expression and the percentage of clonal CD3^+^ malignant cells in the blood of patients with 17q gains (*n* = 12; *STAT3*: Spearman *r* = 0.73, *P* = 0.009; *STAT5A*: Spearman *r* = 0.63, *P* = 0.03 and *STAT5B*: Spearman *r* = 0.68, *P* = 0.02; Fig EV1F–H).

Immunoblot analyses of STAT3/5 total protein levels in a set of representative patient samples (*n* = 3) and healthy controls (*n* = 3) revealed an upregulation in P4 and P5, in contrast to P6 and healthy controls, in concordance with the presence of *STAT3/5* copy number gain in P4 and P5, and its absence in P6 and healthy controls (Fig 2G). Furthermore, to investigate if total STAT3/5 protein expression and activation are prominent in L‐CTCL patient tumor tissue, we performed immunohistochemical staining of skin biopsy sections from P1 to P6, as well as from five healthy individuals (H1–H5), using CD3 as a T‐cell marker, and STAT3, STAT5A, STAT5B, and pY‐STAT5 as markers of STAT5 activation. We found dense infiltrates of STAT3^+^/STAT5A^+^/STAT5B^+^/pY‐STAT5^+^ cells in CD3^+^ regions of the dermal and subcutaneous layer of the skin in patients, where the percentage of positive cells was significantly increased for STAT5A/B and pY‐STAT5 in patients compared to healthy controls (Fig 2H–M and Appendix Fig S3). Moreover, CD3/pY‐STAT5 double staining from P3 demonstrated that 13.4% of the CD3^+^ infiltrates are pY‐STAT5^+^ cells (Fig 2H). Overall, we conclude that *STAT3/5* copy number gains, detected as the main genetic aberration in our L‐CTCL cohort, result in enhanced STAT3/5 expression and hyperactivation, indicating that enhanced oncogenic STAT3/5 activation could be an important disease‐driving event.

### Pharmacologic targeting of STAT3/5 using direct small‐molecule degraders and IQDMA is effective *in vitro*

**Figure 3:**
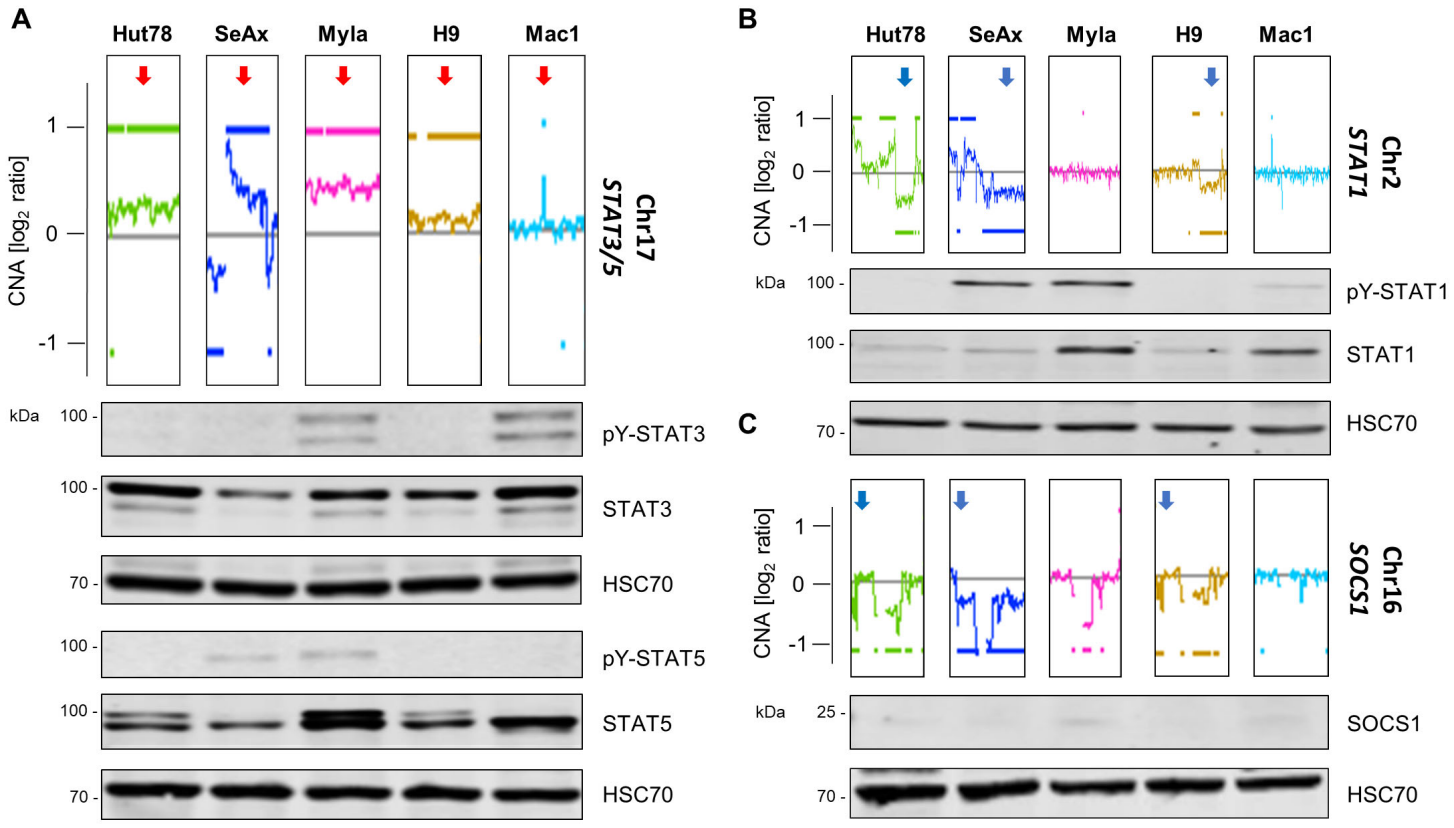
Established CTCL cell lines carrying *STAT3/5* gains can be employed as patient‐like models for *in vitro* testing A–CGraphic depicts the aberration patterns on (A) chromosome 17 (*STAT3/5*), (B) chromosome 2 (*STAT1*), and (C) chromosome 16 (*SOCS1*) in the CTCL cell lines used in the study. Aberration patterns are represented as the log_2_ ratio of the fluorescence intensity of the tumor DNA vs. reference DNA and were generated by aCGH. The whole‐chromosome aberration patterns of the cell lines are shown in Appendix Fig S4. Immunoblotting for total protein STAT1/3/5 and activation levels: phospho‐Tyr (701)–STAT1/phospho‐Tyr (705)–STAT3/phospho‐Tyr (694/699)–STAT5 and SOCS1 protein levels. The cytokine‐dependent cell line, SeAx, was collected 30 min after 5 ng/ml IL‐2 and IL‐4 cytokine addition. HSC70 was used as loading control. One representative out of three independent experiments is shown. Source data are available online for this figure.

To assess if human CTCL cell lines possess similar structural genomic variations and could serve as models for L‐CTCL, we performed array comparative genomic hybridization (aCGH). We selected five cell lines derived from patients with leukemic and advanced CTCL disease, namely SS (Hut78, SeAx, H9), advanced‐stage MF (Myla), and another advanced CTCL disease type (Mac1; Table EV4). Similar incidences of genomic abnormalities to those in primary samples were observed in Hut78, SeAx, Myla, H9, and Mac1 cells, which all carried *STAT3/*5 gains (Fig 3A and Appendix Fig S4, Dataset EV3. Array comparative genomic hybridization). Interestingly, the Mac1 cell line, which originates from a patient with CD30^+^ CTCL, possesses a gain of a small region on 17q containing specifically the *STAT3/5* gene loci (Fig 3A and Appendix Fig S4E, Dataset EV3. Array comparative genomic hybridization). Other significant similarities to patient samples included *MYC* amplification via 8q gains in Hut78, SeAx, H9, and Mac1 cells, *TP53* loss via 17p deletion in Hut78 and SeAx cells, as well as *STAT1* and *SOCS1* losses in Hut78, SeAx, and H9 cells (Fig 3B and C, and Appendix Fig S4, Dataset EV3. Array comparative genomic hybridization). Surprisingly, Myla and H9 cells displayed a *TP53* gain, but a previously reported STOP codon mutation at amino acid 103 of the TP53 protein in Myla cells indicates functional loss of checkpoint control (Fig 3A and Appendix Fig S4C and D, Table EV4, Dataset EV3. Array comparative genomic hybridization).

Robust STAT3/5 total protein and activation (pY‐STAT3/5) levels as detected by immunoblotting were consistent with the presence of *STAT3/5* gains (Fig 3A). Surprisingly, Hut78, despite carrying *JAK1/3* activating mutations and a *STAT3/5* gain, does not show detectable pY‐STAT5 levels when cultured (Fig 3A and Table EV4). However, Hut78 possesses higher total and activated STAT3 (Fig 3B and Table EV4), suggesting negative regulatory action by phosphatases and enhanced STAT3 oncogene action. Importantly, the detected *STAT1* and *SOCS1* losses were consistent with lower total protein STAT1/SOCS1 levels in Hut78, SeAx, and H9 cells (Fig 3B and C). We conclude that established human CTCL cell lines recapitulate genomic aberrations found in L‐CTCL patient samples. We, therefore, used them as models to investigate dual STAT3/5 targeting.

**Figure 4:**
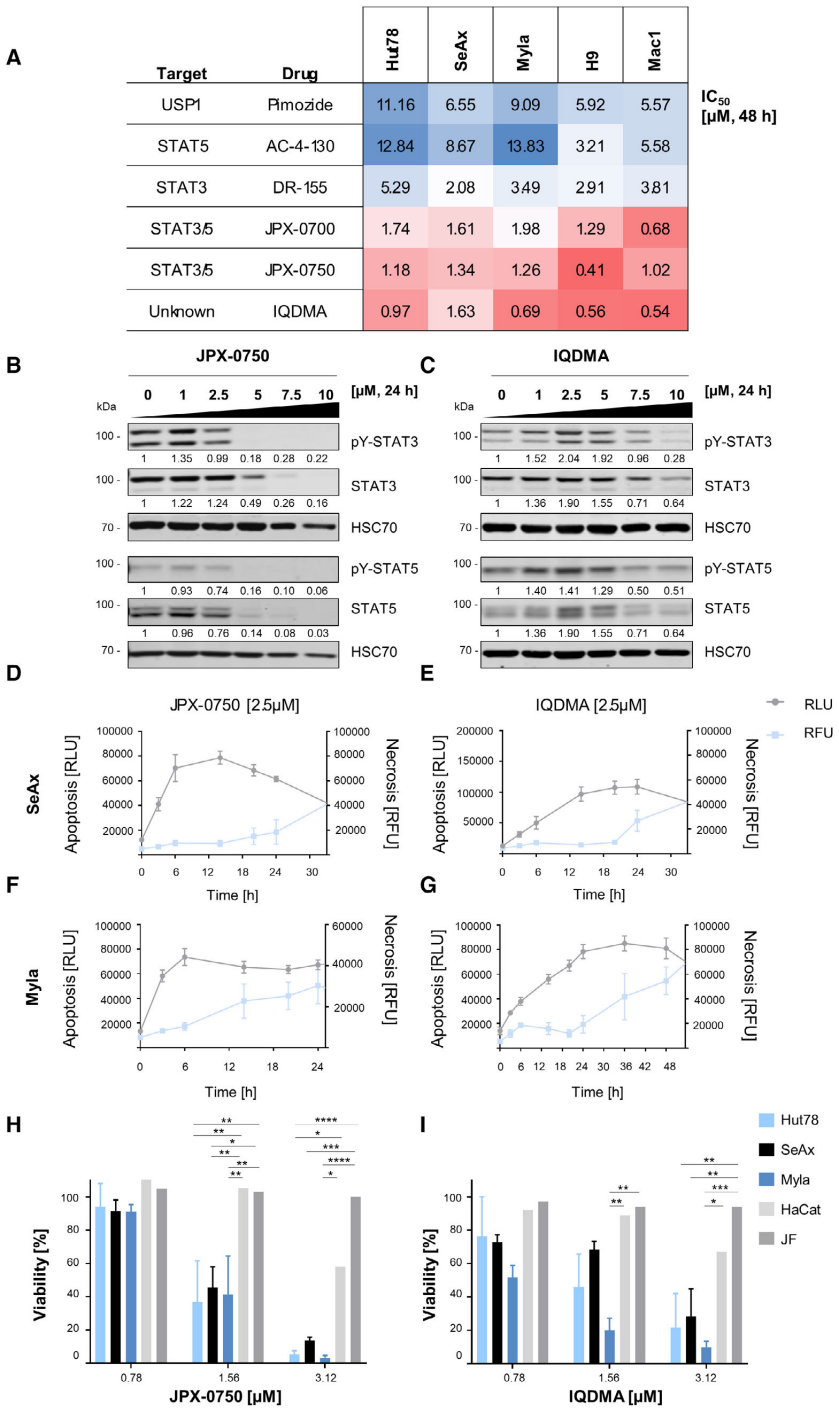
JPX‐0750 and IQDMA are potent, apoptosis‐inducing, and L‐CTCL cell‐specific compounds with distinctive modes of action AHeatmap of IC_50_ values calculated from drug–response analysis using CellTiter‐Glo viability assays upon 48 h drug treatment. One representative, out of three independent experiments performed in triplicates, is shown.B, CImmunoblot showing total protein STAT3/5 and activation levels: phospho‐Tyr (705)–STAT3/phospho‐Tyr (694/699)–STAT5A/B after 24 h treatment with (B) JPX‐0750 and (C) IQDMA. The cytokine‐dependent cell line, SeAx, was collected 30 min after 5 ng/ml IL‐2 and IL‐4 cytokine addition. HSC70 was used as loading control. The normalized levels of phospho‐ and total STAT3/5 quantified by densitometry are shown below the respective blots. One representative out of three independent experiments is shown.D–GReal‐time apoptosis/necrosis assay in the (D, E) SeAx and (F, G) Myla cell lines treated with 2.5 μM JPX‐0750 and IQDMA. RLU, Apoptosis [RLU] = relative luminescence; Necrosis [RFU] = relative fluorescence units. Error bars represent mean ± SD. One experiment in technical triplicates was performed.H, IThe effect of (H) JPX‐0750 and (I) IQDMA on the viability of CTCL cells in comparison to the viability of primary juvenile fibroblasts (JF) and keratinocytes (HaCat) at a low‐dose range (0.7–3 μM). One experiment, in triplicates, was performed with JF and HaCat cells, and three experiments, in triplicates, with the CTCL cells. Error bars represent mean ± SD. Statistical significance was calculated by two‐way ANOVA with Tukey's multiple comparisons test. *P*‐value: < 0.05 (*), < 0.01 (**), < 0.001 (***), and < 0.0001 (****). *P*‐value summaries are provided in Appendix Table S1. Source data are available online for this figure.

**Figure EV2:**
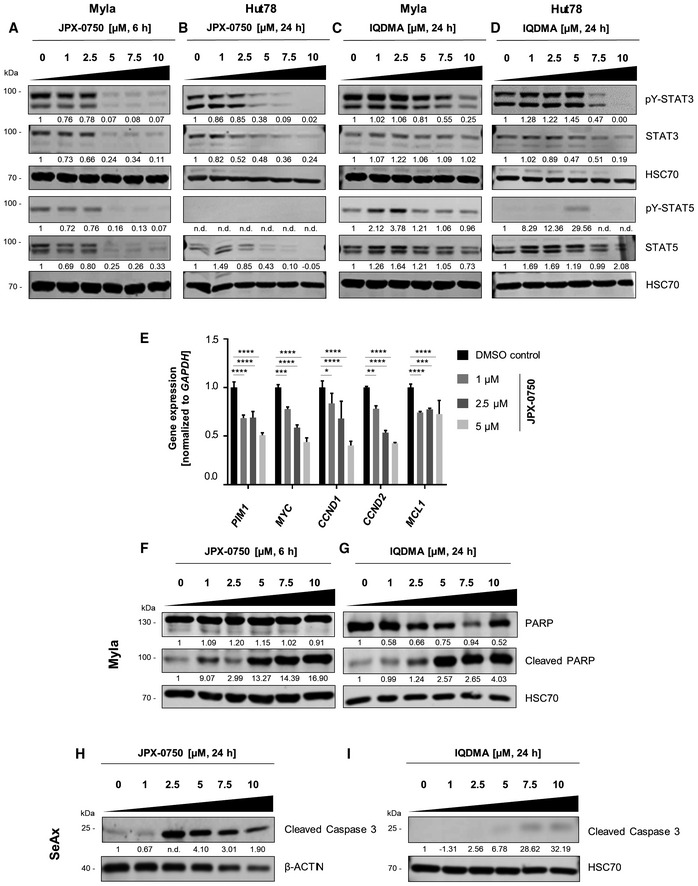
JPX‐0750 and IQDMA have distinct mechanisms of action A–DImmunoblot showing total and pY‐STAT3/pY‐STAT5 levels (i.e., phospho‐Tyr (705)‐STAT3 and phospho‐Tyr (694/699)‐STAT5A/B) in (A) Myla cells upon 6 h treatment with JPX‐0750 and (B) Hut78 cells upon 24 h treatment with JPX‐0750. (C) Myla and (D) Hut78 cells were treated with 24 h IQDMA. HSC70 was used as loading control. The normalized phospho‐ and total protein levels, quantified by densitometry, are shown below the respective blots. One representative out of three independent experiments is shown.ERT–qPCR for STAT5 target genes in the SeAx cell line after 24 h treatment with JPX‐0750. Gene expression was normalized to *GAPDH*. Statistical significance was calculated using two‐way ANOVA with multiple comparisons. *P*‐value: < 0.05 (*), < 0.01 (**), < 0.001 (***), and 0.0001 (****). *P*‐value summaries are provided in Appendix Table S1. Error bars represent mean ± SD. One experiment performed in triplicates is shown.F, GCleaved and total PARP protein levels in Myla cells treated with (F) JPX‐0750 for 6 h and (G) IQDMA for 24 h. HSC70 was used as loading control. The normalized total protein levels, quantified by densitometry, are shown below the respective blots. One representative out of two independent experiments is shown.H, IImmunoblotting for cleaved caspase 3 levels in SeAx cells after 24 h treatment with (H) JPX‐0750 and (I) IQDMA. β‐Actin or HSC70 were used as loading controls. The normalized total protein levels, quantified by densitometry, are shown below the respective blots. One representative out of two independent experiments is shown. Source data are available online for this figure.

We evaluated a series of confirmed and putative STAT3 and STAT5 inhibitors with varying chemotypes and target engagement profiles. These included four penta‐fluoro‐benzene sulfonamide‐based scaffolds (AC‐4‐130 (Wingelhofer *et al*, 2018), JPX‐0700, JPX‐0750 (Park *et al*, 2020), and DR‐155 (Kosack *et al*, 2019)), a benzimidazole analog (pimozide; Mistry *et al*, 2013), and an indoloquinoline derivative (IQDMA; Chien *et al*, 2008; Yang *et al*, 2008; Fig 4A). Distinguishing between the different direct STAT3/5 inhibitors based on potency of reducing cell viability (IC_50_) revealed that the newest generation of small‐molecule monovalent dual STAT3/5 degraders, JPX‐0700 and JPX‐0750 (IC_50_ range 0.41–1.74 μM), are much more potent than previously published inhibitors that target individual STATs, such as the STAT3 inhibitor DR‐155 (IC_50_ range 2.08–5.29 μM), or the STAT5 inhibitor AC‐4‐130 (IC_50_ range 3.21–13.83 μM; Fig 4A). Additionally, dual STAT3/5 degradation using JPX‐0700 or JPX‐0750 was also more efficient at inducing CTCL cell death than indirect STAT5 inhibition with pimozide (IC_50_ range 5.57–11.16 μM), which targets enzymes of ubiquitinylation upstream of STAT5 (Fig 4A). JPX‐0750 was effective at inducing STAT3/5 degradation in the cytokine‐dependent SeAx cells, as well as in Myla and Hut78 cells (Figs 4B, and EV2A and B). Notably, total STAT3‐degrading effects in the SeAx cells occurred at higher concentrations than STAT5 degradation, indicating that the anti‐STAT5 activity of this compound occurs more rapidly (Fig 4B). In line with this, JPX‐0750 significantly downregulated STAT5 target genes, *MYC*, *PIM1*, *D Type Cyclins*, and *MCL1* in the SeAx cell line after 24 h drug treatment (Fig EV2E and Appendix Table S1). Interestingly, IQDMA, previously classified as a STAT5 inhibitor, was similarly potent as the dual small‐molecule degraders (IC_50_ range 0.54–1.63 μM), questioning the perceived mode of action (Fig 4A; Lu *et al*, 2010). Immunoblotting of whole‐cell protein extracts showed that IQDMA decreased activated and total STAT5 levels in SeAx cells, activated STAT3 in SeAx, Myla, and Hut78 cells, and total STAT3 in SeAx and Hut78 cells, however, at 5‐7‐fold higher concentrations (7.5–10 μM) than the observed IC_50_ (0.69–1.63 μM), suggesting that the effect was rather indirect (Figs 4C, and EV2C and D).

Both, JPX‐0750 and IQDMA, reduced cell viability by inducing classical apoptosis, as demonstrated by caspase‐3/PARP cleavage in SeAx/Myla cells, respectively (Fig EV2F–I). Measuring the kinetics of apoptosis induction revealed that JPX‐0750 exhibits its effect considerably faster than IQDMA at the same concentration (2.5 μM), with a maximum number of cells in early apoptosis detectable already after 12 h in SeAx cells, and after 6 h in Myla cells (Fig 4D–G). This is in line with the observed total STAT3/5 degradation in Myla cells occurring already after 6 h treatment with JPX‐0750 (Fig EV2A). In contrast, IQDMA exhibited slower and weaker STAT3/5 inactivation in both cell lines, with the maximum number of cells apparent in early apoptosis after 24 h treatment (Fig 4D–G), again suggesting its rather indirect effect on STAT3/5.

In order to investigate whether these two potent compounds have a significant cytotoxic effect on other non‐malignant human cells, including those that reside in close proximity to malignant L‐CTCL cells in the skin, such as fibroblasts or keratinocytes, we treated primary human fibroblasts (i.e., juvenile fibroblasts, JF) and a keratinocyte cell line (HaCat) with JPX‐0750 and IQDMA. Comparing the viability of primary human fibroblasts and HaCat cells with CTCL cells (Hut78, SeAx, and Myla) at a low dose range (0.7–3 μM) revealed that both JF and HaCat cells remain 90% viable at the effective concentrations that kill L‐CTCL cells, indicating a therapeutic window for using these drugs (Fig 4H and I, and Appendix Table S1). In summary, we identified JPX‐0750 and IQDMA as potent, apoptosis‐inducing, and malignant cell‐specific compounds that block STAT3/5 signaling with distinctive modes of action.

### IQDMA is a broad‐range multi‐kinase inhibitor that suppresses STAT3/5 nuclear levels

**Figure 5:**
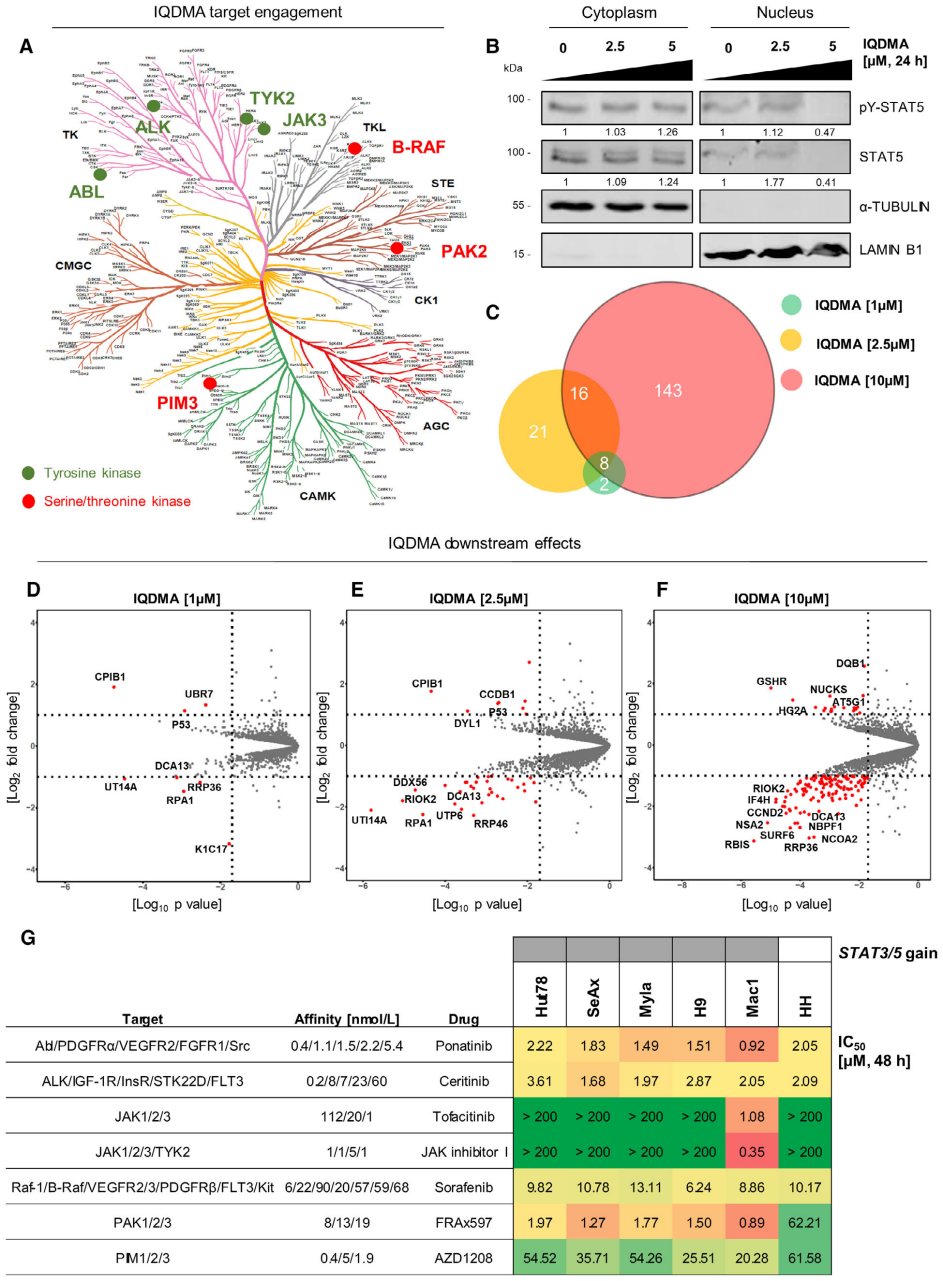
IQDMA is a multi‐kinase inhibitor downregulating STAT5 nuclear levels AKinome screen with 10 μM IQDMA.BSubcellular fractions of SeAx cells treated with IQDMA for 24 h and immunoblotted for pY‐STAT5 and total STAT5. The cells were collected 30 min after 1 ng/ml IL‐2 cytokine addition. α‐Tubulin and histone H3 were used as loading controls for cytoplasmic and nuclear fractions, respectively. The normalized levels of phospho‐ and total STAT5 in the nucleus and cytoplasm, quantified by densitometry, are shown below the respective blots. One representative out of two independent experiments is shown.CVenn diagram showing the number of proteins that are significantly up‐/downregulated as a result of IQDMA treatments at 1, 2.5, and 10 μM.D–FScatter plot depicting the profile of SeAx cells treated with (D) 1 μM IQDMA, (E) 2.5 μM IQDMA, and (F) 10 μM IQDMA. Protein abundances were determined using TMT‐based quantification mass spectrometry. Significant changes were assessed by a modified *t*‐test as implemented in the limma package, with the negative log_10_ *P*‐values on the *x*‐axis, and log_2_ fold change shown on the *y*‐axis.GHeatmap of IC_50_ values calculated from drug response analysis using CellTiter‐Blue or CellTiter‐Glo viability assays upon 48 h drug treatment. One representative of three independent experiments performed in triplicates is shown. Gray represents the presence of *STAT3/5* gain. Source data are available online for this figure.

**Figure EV3:**
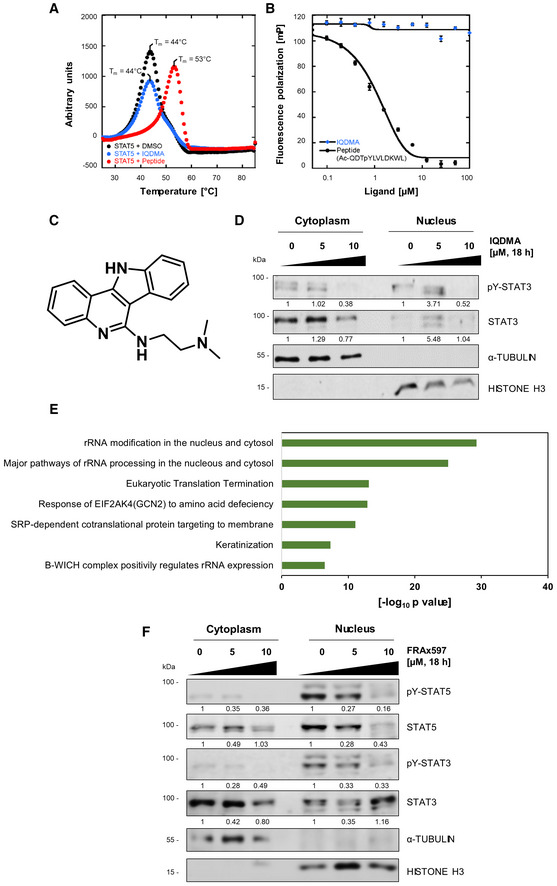
In‐depth analysis of IQDMA shows its multi‐kinase inhibitory function AThermal shift profiles of 2 μM STAT5B with 100 μM IQDMA or 100 μM STAT5‐binding peptide (positive control). The local maxima indicate the melt temperature of the protein (T_m_ ± 0.3°C). One representative out of two independent experiments performed in technical triplicates is shown.BFluorescence polarization assay using 180 nM STAT5B with 100 μM IQDMA or 100 μM peptide (positive control). The positive control exhibits a single‐site displacement profile. Error bars represent mean ± SD. Three independent experiments were performed.CIQDMA structure.DSubcellular fractions of SeAx cells treated with IQDMA for 18 h and immunoblotted for pY‐STAT3 (phospho‐Tyr (705)‐STAT3) and total STAT3. The cells were collected 30 min after 5 ng/ml IL‐2 cytokine addition. α‐Tubulin and histone H3 were used as loading controls for cytoplasmic and nuclear fractions, respectively. The normalized levels of phospho‐ and total STAT3 in the nucleus and cytoplasm, quantified by densitometry, are shown below the respective blots. One experiment was performed.EBar graph showing the cellular pathways that are most affected by IQDMA treatment as determined by network enrichment analysis.FSubcellular fractions of SeAx cells treated with FRAx597 for 18 h and immunoblotted for pY‐STAT3/pY‐STAT5 levels (i.e., phospho‐Tyr (705)‐STAT3 and phospho‐Tyr (694/699)‐STAT5A/B) and total STAT3/5 levels. The cells were collected 30 min after 5 ng/ml IL‐2 cytokine addition. α‐Tubulin and histone H3 were used as loading controls for cytoplasmic and nuclear fractions, respectively. The normalized levels of phospho‐ and total STAT3/5 in the nucleus and cytoplasm, quantified by densitometry, are shown below the respective blots. One experiment was performed. Source data are available online for this figure.

The remarkable potency of IQDMA prompted us to further investigate its mode of action. First, we validated whether IQDMA engages STAT5, using thermal shift and fluorescence polarization assays. Both assays demonstrated that IQDMA does not directly bind STAT5 (Fig EV3A and B). Given its structure displaying an adenosine backbone reminiscent of kinase inhibitors, we performed a kinome screen to broadly examine kinase engagement (Figs 5A and EV3C). Interestingly, the IQDMA target engagement profile included several kinases from the tyrosine (ABL, ALK, TYK2, and JAK3) and the serine/threonine (B‐RAF, PAK2, and PIM3) kinase families (Fig 5A and Dataset EV4. Kinome screen). Since it was reported that upstream kinases, such as PAK, can regulate the nuclear shuttling of STAT5, we investigated whether IQDMA has an impact on STAT5 nuclear levels (Berger *et al*, 2014; Chatterjee *et al*, 2014). In contrast to the cytoplasmic protein, total and activated STAT5 nuclear levels were downregulated in the SeAx cells after 24 h treatment at 5 μM, indicating that this might be a possible mechanism of action (Fig 5B). In the case of STAT3, activated nuclear levels were downregulated at 10 μM after 24 h treatment (Fig EV3D).

To examine IQDMA‐specific effects in a dose‐dependent manner without limiting the analysis to only kinases, we carried out global proteomics profiling of SeAx extracts after 24 h treatment with 1, 2.5 and 10 μM IQDMA (Fig 5C–F). The differential proteins affected in response to increasing IQDMA concentrations were found to be related to multiple cellular processes in ribosomal RNA processing/modification, protein translation/modification, metabolism, cell cycle progression, and surprisingly, keratinization (Figs 5D–F and EV3E, Dataset EV5. Global proteomics). Insight into the most significant differentially affected proteins at a low dose (1 μM) revealed downregulation of key regulators of proliferation, which also regulate the immune response, such as K1C17, a type I intermediate filament chain Keratin 17 protein. K1C17 is reportedly not expressed in normal T‐cells, but the corresponding gene, *KRT17*, was previously shown to be upregulated in CTCL skin lesions (Fig 5D and Dataset EV5. Global proteomics; Shin *et al*, 2007). Importantly, downregulation of a STAT3/5 downstream target involved in cell cycle progression, CCND2, was evident at 10 μM (Fig 5F and Dataset EV5. Global proteomics).

To compare the effect of IQDMA with other commonly used kinase drugs, we performed a focused drug screen, using either clinically approved kinase drugs, such as ponatinib (ABL), ceritinib (ALK), tofacitinib (JAK1/3), and sorafenib (B‐RAF), or drugs in pre‐clinical evaluation, such as FRAx597 (PAK1/2/3), AZD1208 (PIM1/2/3), and a non‐clinical broad‐range JAK inhibitor (JAK1/2/3/TYK2; Fig 5G). To investigate whether the effect of IQDMA is specific for cells with *STAT3/5* gains, we also included a human CTCL cell line that does not have 17q (*STAT3/5*) gain as control, the HH cells (Dataset EV3. Array comparative genomic hybridization). The inhibitor analysis highlighted the sensitivity of all cell lines to ponatinib (IC_50_ range 0.92–2.22 μM), ceritinib (IC_50_ range 1.68–3.61 μM), and sorafenib (IC_50_ range 6.24–13.11 μM), irrespective of *STAT3/5* gains (Fig 5G). In contrast, all cells were poorly responsive to AZD1208 (IC_50_ > 20 μM), and unresponsive to JAK kinase inhibition (IC_50_ > 200 μM), with exception of Mac1 cells (IC_50_ = 1.08 μM for tofacitinib and 0.35 μM for JAK inhibitor I) explained by the presence of a PCM1‐JAK2 driver kinase fusion (Fig 5G and Table EV4). Interestingly, the PAK inhibitor, FRAx597, was quite potent and specifically killed Hut78, SeAx, Myla, H9, and Mac1 cells with *STAT3/5* gains (IC_50_ range 0.89–1.97 μM), whereas the HH cells that do not have *STAT3/5* gain were unresponsive (IC_50_ = 62.21 μM; Fig 5G). Taken together, we conclude that IQDMA is a broad‐range multi‐kinase inhibitor that may indirectly block STAT3/5 nuclear translocation, but further investigation is required to explain the exact mechanism of action. Importantly, (multi)kinase inhibition, particularly of the PAK kinase, emerged as an effective targeting approach for L‐CTCL cells carrying *STAT3/5* gains.

### Upstream kinase inhibition, particularly of the PAK kinase, is effective in L‐CTCL and suppresses STAT3/5 downstream signaling

**Figure 6:**
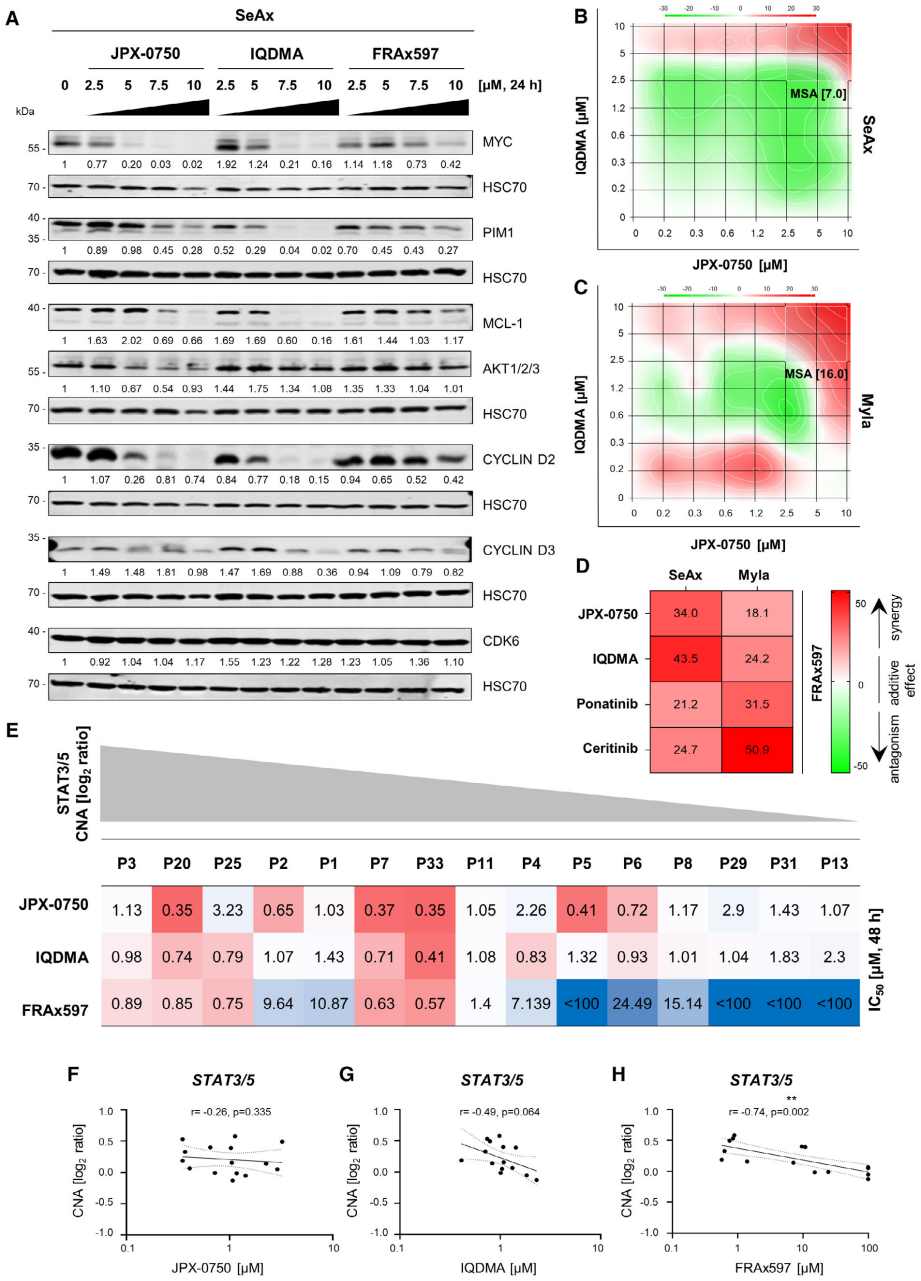
The PAK kinase inhibitor impacts downstream STAT3/5 signaling can be used in drug combinations and is highly specific for L‐CTCL cell carrying *STAT3/5* gains AInhibitor treatment was carried out with increased dose escalation for 24 h. SeAx cells were collected 2 h after 7,5 ng/ml IL‐2 and IL‐4 cytokine addition. STAT3/5 target gene products MYC, PIM1, MCL‐1, and AKT1/2/3, as well as proteins regulating the cell cycle, such as CYCLIN D2 and D3, and CDK6, were probed by western blotting. HSC70 served as loading control. The normalized protein levels, quantified by densitometry, are shown below the respective blots. One representative out of three independent experiments is shown.B–DSynergy analysis of the indicated two‐drug combinations in the SeAx and Myla cells after 48 h treatment. In each graph, the most synergistic area (MSA) is highlighted, which represents the most synergistic three‐by‐three dose window with the respective MSA score. The zero‐interaction potency model was applied to quantify the degree of synergy, according to which an MSA score below −10 indicates that drugs are antagonistic (green), a score between −10 and 10 indicates that two drugs are additive (white), while a score above 10 indicates a synergistic effect (red). (D) Heatmap showing MSA scores of the combination of FRAx597 with the listed drugs. One representative out of two independent experiments is shown. Synergy matrices for each drug combination are represented in Appendix Fig S5.EHeatmap showing the IC_50_ values upon treatment of primary PBMCs isolated from L‐CTCL patients with JPX‐0750, IQDMA, and FRAx597. One experiment performed in triplicates using CellTiter‐Glo viability assays upon 48 h drug treatment is shown. The relative STAT3/5 log_2_ ratio value is depicted in gray.F–HSpearman correlation analysis using CNA log_2_ ratios and the IC_50_ values for the respective drug. Source data are available online for this figure.

We further investigated the mechanism of action of IQDMA on STAT3/5 signaling in comparison to the direct STAT3/5 degrader JPX‐0750 and the PAK kinase inhibitor FRAx597. We performed immunoblotting analysis of proteins directly regulated as STAT3/5 target gene products in SeAx cells, which included oncoproteins involved in cell proliferation and survival, such as MYC, PIM1, MCL‐1, AKT1/2/3, and proteins regulating the cell cycle, such as CYCLIN D2 and D3, and CDK6. We observed a similar effect of JPX‐0750 and IQDMA on STAT3/5 pathway targets, as both downregulated MYC, MCL‐1, and CYCLIN D2 at 7.5 μM after 24 h of treatment (Fig 6A). However, IQDMA was more effective in downregulating PIM1, MCL‐1, and CYCLIN D3 (Fig 6A). No effect was observed on CDK6, and only JPX‐0750 had an effect on AKT1/2/3 (Fig 6A). In contrast, the PAK inhibitor FRAx597 exhibited downregulating effects on MYC, PIM1, and CYCLIN D2 at 10 μM after 24 h treatment (Fig 6A). Assessing the effect of FRAx597 on cytoplasmic and nuclear STAT3/5 levels revealed that it reduces both activated and total STAT3/5 in the cytoplasm and nucleus at 5 μM (Fig EV3F). These data suggest that both IQDMA and FRAx597 suppress STAT3/5 nuclear translocation and downstream signaling.

Additionally, given that effective therapeutic strategies often require combinatorial drug administration, we tested the synergistic effects between these drugs (Fig 6B–D). While the combination of JPX‐0750 and IQDMA only had additive effects in SeAx cells, it was strongly synergistic in Myla cells (Fig 6B and C). Notably, the combination of FRAx597 with JPX‐0750, IQDMA, ponatinib, or ceritinib was synergistic in both cell lines, indicating that drug combinations targeting both STAT3/5 signaling and broad‐spectrum kinases, particularly PAK, appear to be effective in killing L‐CTCL cells (Fig 6D and Appendix Fig S4).

### PAK kinase inhibition is specific for L‐CTCL patient cells carrying *STAT3/5* gains *ex vivo*

**Figure EV4:**
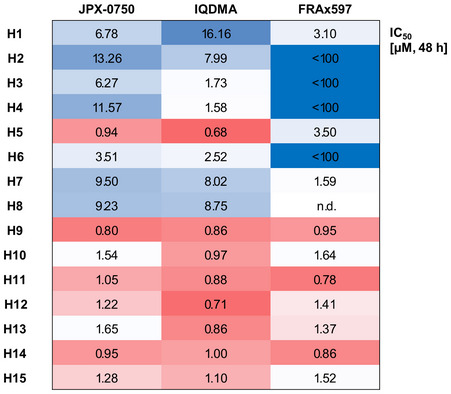
JPX‐0750, IQDMA, and FRAx597 show varying toxicity to healthy cells Heatmap showing IC_50_ values upon treatment of primary PBMCs isolated from healthy controls with JPX‐0750, IQDMA, and FRAx597. One experiment performed in triplicates using CellTiter‐Glo viability assays upon 48 h drug treatment is shown. The relative STAT3/5 log_2_ ratio value is depicted in gray. Source data are available online for this figure.

We next compared the effect of the three different drugs on primary patient samples (*n* = 15). JPX‐0750 and IQDMA were potent in killing primary patient cells with varying IC_50_ values (JPX‐0750: IC_50_ range 0.35–2.9 μM; IQDMA: IC_50_ range 0.41–2.3 μM; Fig 6E). However, in contrast to JPX‐0750 and IQDMA, the PAK inhibitor, FRAx597, showed remarkable specificity for cells with *STAT3/5* copy number gains (Fig 6E–H). For FRAx597, the responses correlated inversely with the respective *STAT3/5* CNA log_2_ ratios (Spearman *r* = −0.74, *P* = 0.002; Fig 6H). Furthermore, we tested the toxicity of the compounds on healthy donor PBMCs isolated from either younger (H1–H8) or elderly individuals (H9–H15; Fig EV4). While the PBMCs from younger individuals were generally less responsive to all three drugs (JPX‐0750: IC_50_ range 0.94–13.26 μM, IQDMA: IC_50_ range 0.68–16.16 μM, FRAx597: IC_50_ range 1.59 to < 100 μM), the PBMCs from elderly individuals displayed low IC_50_ values (JPX‐0750: IC_50_ range 0.80–1.65 μM, IQDMA: IC_50_ range 0.71–1.10 μM, FRAx597: IC_50_ range 0.78–1.64; Fig EV4).

Overall, we conclude that targeting L‐CTCL cells carrying *STAT3/5* copy number gains can be achieved by blocking the STAT3/5 pathway either by direct dual‐covalent inhibitor‐mediated degradation or targeting upstream kinases such as PAK, which shows specificity toward cells with *STAT3/5* gains, but with an unknown mechanism of action potentially involving STAT3/5 nuclear shuttling.

### Upstream (multi)kinase inhibition attenuates L‐CTCL growth in a xenograft mouse model

**Figure 7:**
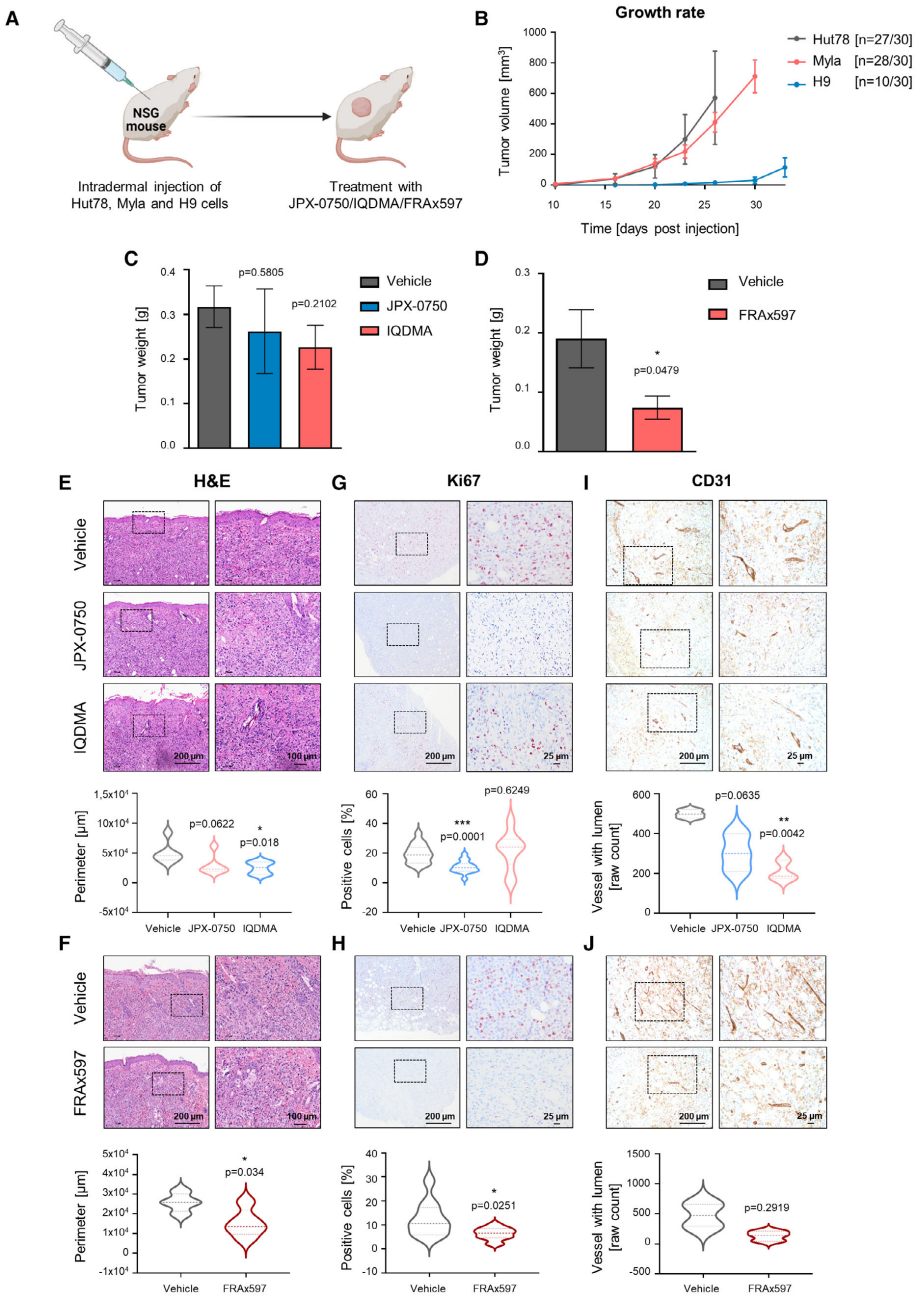
IQDMA and PAK kinase inhibitor are highly effective in reducing tumor growth in an intradermal xenograft mouse model AGraphical scheme depicting the experiment.BGrowth rate of Hut78, Myla, and H9‐derived tumors measured on the respective day post‐injection. The mean of the respective tumor volumes for the H9 group and vehicle‐treated groups for Hut78 and Myla are shown. Tumor volume = (length × width^2^)/2. Error bars represent mean ± SEM. *n* represents the total number of tumors that engrafted per group.C, DMyla‐derived tumor weight upon experiment termination, after 20 days of treatment with 5 mg/kg of (C) JPX‐0750 (*n* = 5) and IQDMA (*n* = 6), or vehicle (*n* = 7), and (D) FRAx597 (*n* = 5) or vehicle (*n* = 4), with *n* representing the number of analyzed tumors per group. Statistical significance was calculated using a two‐tailed paired *t*‐test. *P*‐value: < 0.05 (*). Error bars represent mean ± SEM.E–JH&E and IHC analyses of Myla‐derived tumors treated with JPX‐0750 (*n* = 6), IQDMA (*n* = 6) or vehicle (*n* = 6), and FRAx597 (*n* = 5) or vehicle (*n* = 4), stained with (E, F) H&E, (G, H) Ki67, as a marker for proliferation, and (I, J) CD31, as a tumor vessel marker, with *n* representing the number of analyzed tumors per group. The pictures shown are from contiguous sections. Dotted rectangles indicate magnified areas. Scale bars, 200, 100, and 25 μm. Violin plots show the perimeter of the annotated tumor cell infiltration/expansion region into the intradermal and subcutaneous region of the skin, as well as quantification of the percentage of Ki67^+^ cells in the tissue. CD31 staining was quantified as raw counts of vessels with lumen. Statistical significance was calculated using a two‐tailed paired *t*‐test with Welch's correction. *P*‐value: < 0.05 (*), < 0.01 (**), and < 0.001 (***). The bold dashed line in the middle of the violin plot denotes the median value, while the thin dotted lines denote the interquartile range. Source data are available online for this figure.

Finally, we evaluated our pharmacologic strategy *in vivo* using a xenograft mouse model in which human CTCL cell lines with *STAT3/5* gains, namely Hut78, Myla, and H9, were injected intradermally into NSG mice, as previously described (Thaler *et al*, 2004; Doebbeling, 2010; Nicolay *et al*, 2016; Froehlich *et al*, 2019; Fig 7A). We administered 1 × 10^6^ Hut78 or Myla cells, or 3 × 10^6^ H9 cells per injection (*n* = 30 injections/cell line). The cell lines showed varying growth rates and success rates of tumor formation, which was 90% for Hut78 cells (*n* = 27/30), 93% for Myla cells (*n* = 28/30), and 40% for H9 cells (10/30), resulting in the exclusion of H9 from further analyses (Fig 7B).

**Figure EV5:**
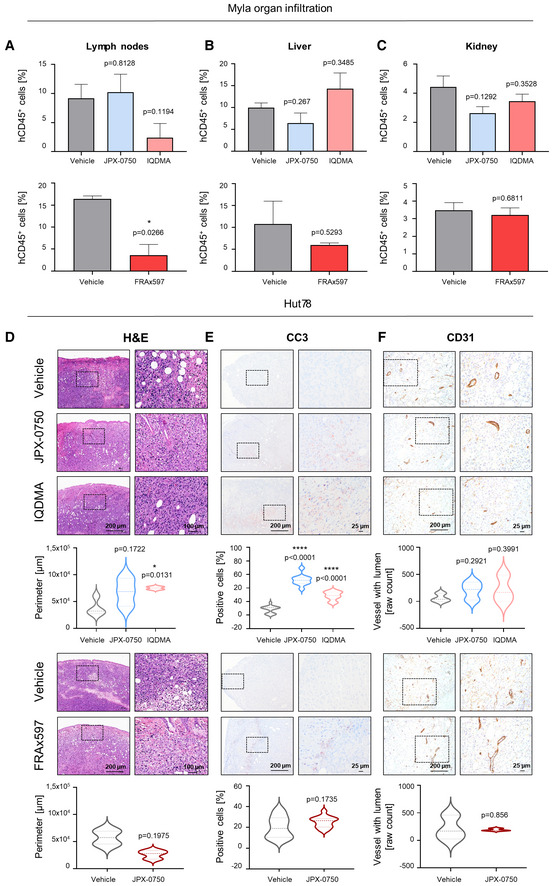
The PAK kinase inhibitor inhibits malignant cell dissemination into the lymph nodes *in vivo* A–CFlow cytometric analysis of malignant cell dissemination into (A) lymph nodes, (B) liver, and (C) kidney (Vehicle: *n* = 3, JPX‐0750: *n* = 3, IQDMA: *n* = 3; Vehicle: *n* = 2, FRAx597: *n* = 3, with *n* representing the number of analyzed tumors per group) as measured by the percentage of human CD45^+^ cells in the respective organ. Error bars represent mean ± SEM. Statistical significance was calculated using a two‐tailed paired *t*‐test with Welch's correction. *P*‐value: < 0.05 (*).D–FH&E and IHC analyses of Hut78‐derived tumors treated with JPX‐0750 (*n* = 3), IQDMA (*n* = 2) or vehicle (*n* = 4), and FRAx597 (*n* = 3) or vehicle (*n* = 2), and stained with (D) H&E and (E) cleaved caspase 3 (CC3) to detect cell death and (F) CD31 vessel marker, with n representing the number of analyzed tumors per group. Pictures shown are from contiguous sections. Dotted rectangles indicate magnified areas. Scale bars, 200, 100, and 25 μm. Violin plots show the perimeter of the annotated tumor cell infiltration/expansion region into the intradermal and subcutaneous region of the skin, as well as quantification of the percentage of CC3^+^ cells in the tissue. CD31 staining was quantified as raw counts of vessels with lumen. Statistical significance was calculated using a two‐tailed paired *t*‐test with Welch's correction. *P*‐value: < 0.05 (*), 0.0001 (****). The bold dashed line in the middle of the violin plot denotes the median value, while the thin dotted lines denote the interquartile range. Source data are available online for this figure.

Hut78‐ and Myla‐injected mice were treated intraperitoneally (i.p.) with 5 mg/kg of JPX‐0750, IQDMA, and FRAx597 once per day (Appendix Fig S6). No significant loss of body weight or defects in hematopoiesis (white blood cell count and hematocrit) were observed (Appendix Fig S6). Thus, we conclude that the drugs were well tolerated in mice. FRAx597 treatment significantly reduced tumor weights (*P*‐value: 0.0479), whereas both IQDMA and FRAx597 treatment resulted in a significant reduction in tumor size in Myla‐injected mice, as measured by perimeter values of dissected tumors after 20 days of treatment (IQDMA: *P*‐value: 0.018, FRAx597: *P*‐value: 0.034; Fig 7C–F). This was accompanied by a significant reduction in Ki67^+^ cells of the FRAx597‐treated tumors (*P*‐value: 0.0251), indicating decreased proliferation within the tumors (Fig 7H). IQDMA treatment caused a significant reduction in CD31^+^ blood vessels (*P*‐value: 0.0042) indicating decreased angiogenesis within the tumors (Fig 7I and J). JPX‐0750 treatment induced a significant reduction in Ki67^+^ cells in the Myla‐derived tumors (*P*‐value: < 0.0001) alongside a trend toward tumor perimeter/weight reduction (perimeter *P*‐value: 0.0622, weight *P*‐value: 0.5805; Fig 7C, E and G). In line with the disseminating nature of the disease, we also observed infiltration of human CD45^+^ cells into the lymph nodes, liver, and kidney, where FRAx597 treatment significantly reduced dissemination into the lymph nodes (*P*‐value: 0.0266; Fig EV5A–C). Even though not significant, IQDMA also reduced dissemination to lymph nodes (*P*‐value: 0.1194; Fig EV5A).

The Hut78 cell line showed exponential growth in NSG mice and treatment had to be terminated after 10 days due to severe disease progression (Fig 7B and Appendix Fig S6). There was no significant impact on tumor size upon drug treatment, even though FRAx597 showed a trend toward reducing the tumor perimeter (*P*‐value: 0.1975; Fig EV5D). Regardless, we conducted a thorough histopathological analysis revealing that JPX‐0750 and IQDMA significantly increased the amount of cleaved caspase‐3^+^ cells in comparison to vehicle‐treated tumors (JPX‐0750: *P*‐value < 0.0001 and IQDMA: *P*‐value: < 0.0001), indicating that they induced death of tumor cells.

In summary, we were able to evaluate the effect of the drugs on tumor growth and disease progression in an intradermal xenograft CTCL mouse model. We conclude that IQDMA and FRAx597 significantly reduced the growth of Myla‐derived tumors, where especially FRAx597 reduced the dissemination of malignant cells, highlighting that compounds targeting kinases such as PAK could be promising candidates for further lead structure optimization toward finding a curative treatment for L‐CTCL.

## Discussion

Here, we integrated genomic analyses and pharmacologic interventions to investigate key dependencies in L‐CTCL. We found increased expression of STAT3/5 at the mRNA and protein level as a direct consequence of *STAT3/5* copy number gains observed at high frequency (74%) in L‐CTCL patient samples and contributing to increased clonal proliferation. Importantly, loss of the tumor suppressors *STAT1* and *SOCS1* appoint to loss of inhibitory functions and loss of negative feedback control. We show that dual STAT3/5 degradation through small‐molecular covalent inhibitors is potent *in vitro* and *ex vivo*, but blocking of upstream kinase action, such as PAK kinase, is necessary *in vivo* and could represent a promising therapeutic strategy to eradicate malignant cells in L‐CTCL. Overall, our findings reveal a key role of PAK kinase in STAT3/5‐mediated oncogene induction, which should be further exploited for the treatment of L‐CTCL.

Our data suggest that enhanced STAT3/5 activity contributes to clonal selection that enables L‐CTCL progression. This is difficult to prove without a time‐dependent analysis of malignant T‐cell clones and tracking of patients from the time point of diagnosis to the time‐ point of progression detection, bringing also into light the difficult diagnosis of CTCL, where patients are misclassified as benign inflammatory dermatoses (BID; Bagherani & Smoller, 2016). For the majority of cases, it takes an average of 6 years from disease onset until correct diagnosis (Diamandidou *et al*, 1996; Bagherani & Smoller, 2016) due to a lack of proper diagnostic methods/tools. Incorporating additional diagnosis criteria, such as *STAT3/5* copy number gain and STAT3/5 nuclear expression status by immunohistochemistry, could assist in a more accurate classification and subsequent therapeutic intervention of advanced CTCL subtypes, as this was the case with prognostic markers in other mature T‐cell leukemias, such as T‐PLL, PTCL‐NOS, or T‐LGLL (Herling *et al*, 2004).

Even though gain‐of‐function (GOF) mutations of *STAT5B* were reported in a minor fraction (3.6%) of CTCL patients (Park *et al*, 2017), our WES data were negative for GOF *JAK–STAT* mutations, but we consistently found copy number gains of *STAT3/*5 representing a cancer genome hallmark for the majority of L‐CTCL patients. Indeed *STAT5A/B* locus focal amplification was shown to be sufficient to elicit a growth advantage and disease progression in prostate cancer (Haddad *et al*, 2013), and similarly here, the *STAT3/5* genetic locus was amplified to enhance STAT3/5 expression. STAT5 is crucial for T‐cell development, expansion, survival, proliferation, and memory effector function, in contrast to STAT3, which has a more restricted role in T‐cell survival and metabolism (Leone & Powell, 2020). STAT3 is essential for the development and function of Th17 cells controlling inflammation or steering autoimmunity (O'Shea & Plenge, 2012). Importantly, both STAT3 and STAT5 can be cell type‐ or mutational context‐dependent oncoproteins or tumor suppressors, but we conclude that all three gene products, STAT3, STAT5A, and STAT5B, are oncogenes in L‐CTCL. In blood cancer, they usually fulfill cancer driver roles when hyperactivated or when their expression is enhanced, where they can also function redundantly as oncoproteins requiring dual STAT3/5 targeting modes, as for the first time explored here with direct covalent STAT3/5 degraders. STAT3/5 proteins display augmented chromatin remodeling capacity when localized in the nucleus, possibly also independent of tyrosine phosphorylation in the case of STAT5, through interactions with chromatin remodeling machinery, or upon high pY‐STAT5 level via induced DNA looping and higher‐order enhancer complex formation driving oncogene transcription (Orlova *et al*, 2019).

We identified copy number loss of *STAT1* and particularly also of the E3 ubiquitin‐conjugating enzyme *SOCS1* co‐occurring with *STAT3/5* copy number gains. Genetic deletion of *SOCS1*, as a negative regulator of JAK–STAT signaling, has recently been detected in skin biopsies of patients with tumor‐stage mycosis fungoides (Bastidas Torres *et al*, 2018). However, we could only detect very low SOCS1 protein levels via immunoblotting even in cell lines with normal *SOCS1* copy number status, suggesting that its downregulation may be important in L‐CTCL and may also be regulated by other mechanisms. This might be particularly relevant as reduced SOCS1 E3 ligase function was associated with increased FAK1 action in immune cells, and increased FAK1 can also increase PAK kinase signaling (Chatterjee *et al*, 2014; Körholz *et al*, 2021). Importantly, loss of *STAT1*, as a negative growth regulator, co‐occurring with *STAT3/5* gain in L‐CTCL is a novel concept for disease pathogenesis and supports the generation of extremely aggressive CTCL cells resistant to therapy, as previously shown with the generation of IFNα‐resistant Hut78 cells that lacked *STAT1* expression (Sun *et al*, 1998). Therefore, reduced expression of tumor suppressor proteins is a consequence of chromosomal instability of L‐CTCL cells, which could also be relevant in explaining enhanced STAT3/5 oncoprotein action. Furthermore, it is also of interest to investigate *MYC* amplification, even though MYC upregulation was cytokine sensitive in SeAx cells. This could be significant to explain the steering of L‐CTCL cancer cell metabolism and proliferation via cytokine‐triggered STAT3/5‐MYC axis (Leone & Powell, 2020). Thus, high MYC activation might be cytokine dependent at early L‐CTCL progression and MYC pathway interference could be an alternative strategy for targeting. In contrast, reactivation of TP53 might be more challenging due to suppressed TP53 function either through genetic deletion or premature STOP codon mutation, such as in Myla cells.

Another provocative finding was that the majority of L‐CTCL cell lines were unresponsive to JAK kinase blockers, despite the presence of *JAK*–*STAT* mutations/translocations and enhanced *STAT3/5* expression. In contrast, the Mac1 cell line carrying a PCM1–JAK2 kinase driver fusion was almost equally responsive to the tested drugs, including IQDMA and JAK inhibitors, confirming the importance of the driver JAK kinase fusion, compared to other cell lines that were unresponsive to JAK inhibition. However, Mac1, as a CD30‐expressing cell line isolated from a primary cutaneous T‐cell lymphoma patient (Ehrentraut *et al*, 2013), is less representative of aggressive L‐CTCL disease, and it might represent a “rare case.” Other cell lines, such as Hut78 or Myla, even though they carry activating *JAK* mutations, were resistant to JAK blockers but still responsive to IQDMA, emphasizing the importance of upregulated *STAT3/5* gene dosage and the action of upstream kinases culminating in enhanced nuclear STAT3/5 protein levels.

IQDMA enabled us to select potential kinase drugs acting in CTCL cells with *STAT3/5* gains, out of which the most promising one seems to be the PAK inhibitor, even though the mechanism of action remains to be further elucidated potentially by genetic experiments knocking down or genetically deleting PAK kinase(s). We found that both IQDMA and FRAx597 suppress STAT3/5 nuclear levels and downstream signaling. Furthermore, they were both effective in primary patient cells *in vitro*, as well as in reducing tumor growth in a mouse xenograft model *in vivo*. Even though the observation that FRAx597 is effective *in vivo* has previously been shown in Schwannomas (Licciulli *et al*, 2013) and prostate cancer (Wang *et al*, 2020), the effectiveness of PAK inhibition in CTCL has only been moderately investigated (Wang *et al*, 2018, 2019). Confirmatory of our premises, PAK‐silenced CTCL cells exhibited spontaneous apoptosis *in vitro* and a decreased rate of tumor growth in comparison to control cells *in vivo* (Wang *et al*, 2018).

In contrast, JPX‐0750, a small‐molecule degrader was highly potent *in vitro*, but was not effective *in vivo*, highlighting the need to target upstream kinases, but also to conduct medicinal chemistry optimization to reach sufficient *in vivo* pharmacokinetic and pharmacodynamics profiles. IQDMA's planarity and hydrophobicity are structurally suboptimal for clinical use, with limited absorption and potential plasma protein binding. Nevertheless, IQDMA has a unique kinase selectivity profile and the ability to downregulate key kinase targets without inducing off‐target toxicity, which might be exploited through structure‐based design approaches. Thus, although it is a modest lead molecule, it possesses unique structural properties and promising biochemical features that render it a unique starting point for further exploration, especially since the clinical development of PAK kinase inhibitors has been hindered by challenges associated with the identification of potent and biologically functional ligands (Liu *et al*, 2021). Moreover, the fact that Hut78 and Myla cells behaved differently upon intradermal xenografting in NSG mice and also responded differently to drug treatment indicates that indeed, for some L‐CTCL patients, a combination kinase therapy might be necessary, as we also briefly explored here.

We conclude that small‐molecule STAT3/5 degraders and IQDMA, as a broad‐range kinase inhibitor, or the more selective PAK inhibitor, represent effective drugs to battle L‐CTCL. Taken together, direct targeting of STAT3/5 through dual STAT3/5 degraders and/or broad‐range kinase inhibition, by indirect targeting of STAT3/5 nuclear shuttling, could pave the road for novel therapeutic options for L‐CTCL.

## Materials and Methods

### Human patients and healthy samples

Samples from SS patients and advanced leukemic MF patients were collected for this study from the following institutes/university hospitals: (i) Department of Dermatology and Venereology, Medical University of Graz, Graz, Austria, (ii) University Clinic for Dermatology, Venereology and Allergology Innsbruck, Medical University of Innsbruck, Innsbruck, Austria, (iii) University Clinic for Dermatology, Venereology and Allergology, University Hospital Wuerzburg, Wuerzburg, Germany, (iv) Department of Dermatology, University Hospital Mannheim, Mannheim, Germany, and (v) Department of Medicine I, CECAD and CMMC Cologne University, Cologne, Germany. Patient characteristics are shown in Table EV1. All patients, as well as healthy donors, gave their written informed consent before they participated in the study. Experimental laboratory studies were performed on human blood and skin punch biopsy samples. Sections from bio‐banked healthy skin tissue samples were obtained for control purposes from the Department of Pathology, Medical University of Graz. The study was conducted in accordance with the Declaration of Helsinki. The protocol was approved by the Ethics committee of the Medical University of Graz (identification code: 29‐609 ex 16/17) and University Hospital Wuerzburg (identification code: 115/15).

### Immunohistochemistry of patient skin biopsies

Immunohistochemical (IHC) studies were performed on 2‐μm‐thick paraffin‐embedded skin sections, which were rehydrated in xylene and increasing dilutions of ethanol. Antigen retrieval was performed using a TRIS‐EDTA buffer solution (pH 9.0). The following antibodies were used: CD3 (#RM9107‐S0, Thermo Fisher Scientific, USA), STAT3 (9D8; #MA1‐13042, Thermo Fisher Scientific), STAT5A (E289; #ab32043, Abcam, UK), STA5B (#ab235934, Abcam), and pY‐STAT5 (Tyr694/699; #9359, Cell Signaling Technologies/CST, USA). The stained slides were imaged/scanned by the Panoramic Digital Slide Scanner (3DHistech. Ltd, Hungary) and Aperio Digital Pathology Slide Scanners (Leica Biosystems, Germany). IHC images were analyzed with the CaseViewer software (3DHistech. Ltd) and QuPath (Bankhead *et al*, 2017).

### Whole peripheral blood mononuclear cell isolation

Whole peripheral blood mononuclear cells (PBMCs) were isolated from the blood of L‐CTCL patients and healthy donors using Ficoll density gradient separation.

### Identification of malignant clones by TCR Vβ repertoire and sorting

Malignant T‐cell clones were identified from L‐CTCL patients' PBMCs using the TCR Vβ Repertoire kit (Beckman Coulter #PN IM3497, USA). The TCR kit is a multiparametric analysis tool designed for the quantitative determination of the TCRVβ repertoire of human T lymphocytes by flow cytometry. The kit is composed of TCRVβ antibodies corresponding to 24 different specificities and allows about 70% coverage of the normal human TCR Vβ repertoire. Upon identification of the malignant clone, the respective CD3^+^TCRVβ^+^ malignant clones were sorted from patient PBMCs using fluorescence‐activated cell sorting. For P1‐P6, CD19^+^ B‐cells were sorted from the same blood samples for control purposes.

### Nucleic acid extraction

For gene expression analysis with quantitative real‐time polymerase chain reaction (RT–qPCR), RNA was extracted from whole PBMCs from patients and healthy donors using the miRNeasy Mini kit (Qiagen, Germany) or the AllPrep DNA/RNA/Protein Mini kit (Qiagen), following manufacturer's instructions. For WES, DNA was isolated from purified CD3^+^/Vβ^+^ T‐cells and corresponding CD19^+^ non‐malignant B‐cells with the QIAamp DNA Mini kit (Qiagen), as per the manufacturer's instructions. DNA and RNA amounts were measured using a NanoDrop spectrophotometer (Thermo Fisher Scientific).

### RT–qPCR

Total RNA was isolated using the RNeasy® Mini kit (Qiagen), and RNA yield was measured with NanoDrop 2000 Spectrophotometer (Thermo Fisher Scientific). Reverse transcription was performed using the RevertAid First Strand cDNA Synthesis kit (Thermo Fisher Scientific) according to the provided protocol. RT–qPCR was carried out in triplicates using the GoTaq^®^ qPCR Master Mix (Promega, USA) on BioRad CFX 96 and 96 or 384 real‐time PCR system (Bio‐Rad Laboratories, Inc., USA). Results were normalized to GAPDH expression and quantified using the ΔΔC(T) method. Primers are listed in Dataset EV6. Drugs, primers and antibodies used.

### Molecular profiling using WES (P1–P6)

WES was performed using the Nextera DNA Flex Enrichment protocol, according to the manufacturer's recommendation. To analyze genome‐wide somatic copy number alterations (CNAs), shallow WGS (sWGS) of pre‐enriched libraries from the WES was performed on an Illumina MiSeq in a single‐read 150 bp mode. CNAs were called using the ichorCNA algorithm (Adalsteinsson *et al*, 2017), a probabilistic model, implemented as a hidden Markov model (HMM), to simultaneously segment the genome, predict large‐scale CNA and estimate the tumor purity and ploidy of an sWGS sample with a bin size of 1 Mb.

Whole‐exome sequencing (WES) was performed on DNA isolated from sorted malignant CD3^+^/Vβ^+^ T‐cells and corresponding CD19^+^ non‐malignant B‐cells. Hybrid capture was done using the Nextera DNA Flex Enrichment protocol according to the manufacturer's recommendation (mean input 80.8, range 26.3–100 ng). For samples with input lower than 100 ng, 12 instead of 9 PCR cycles were performed during library preparation. Libraries were sequenced on an Illumina NextSeq 550 in a paired‐end sequencing mode (2 × 150 bp), producing a mean of 66 million (range 20–113) and 64 million (range 6–110) paired‐end reads for the neoplastic and benign samples, respectively. Reads were mapped to the reference genome hg19 using the Burrows–Wheeler Aligner (BWA) alignment tool version 0.7.17. The mean depth (defined as the mean number of reads covering the captured coding sequence of a haploid reference) was 123×, with 72% of the target region covering more than 30×. It is of note, though, that due to the low input amount, the CD19^+^ sample of P4 had a significantly lower average exon coverage of 8.7×, while P1 had an exceptionally high number of variants that did not pass the initial QC. To identify somatic mutations, the GATK MuTect2 version 4.1 was used (Blokzijl *et al*, 2018). A stepwise filtering approach was used to identify high‐confidence stem somatic mutations. First, all variants that did not pass the initial quality filter of MuTect2 (PASS) were removed. Second, only variants that were covered at least with 10 alternate reads and a total depth of 50× were kept. Finally, filtering for SNVs with a variant allele fraction (VAF) of ≥ 20% and indels ≥ 5% was applied.

### Molecular profiling using shallow WGS (P1–P6)

To analyze genome‐wide somatic copy number alterations (CNAs), shallow WGS (sWGS) of pre‐enriched libraries from the WES were performed on an Illumina MiSeq in a single‐read 150 bp mode. CNAs were called using the ichorCNA algorithm (Adalsteinsson *et al*, 2017), a probabilistic model, implemented as a hidden Markov model (HMM), to simultaneously segment the genome, predict large‐scale CNA, and estimate the tumor purity and ploidy of an sWGS sample with a bin size of 1 Mb.

### Low‐pass whole‐genome sequencing and mapping sequence reads to the reference genome

The patient samples were sequenced in NovaSeq 2 × 150. Raw BCL files generated by the sequencer were converted to fastq files for each sample using bcl2fastq v.2.20. Raw fastq files were uploaded to the Gencove cloud service for mapping to the Human low‐pass GRCh38 v1.2 [0.1×–6×] genome. After mapping, an imputated variant calling analysis was performed on the Gencove analysis platform to produce SNPs and CNVs using the configuration “Human low‐pass GRCh38 v1.2 [0.1×–6×]” (Gencove capability id: dc5fc85b‐1a9a‐4,198‐bb5f‐7b61fc171e33). This imputation reference panel is the lifted‐over panel from the GRCh37 release and is comprised of the phased release of genotype calls from the New York Genome Center's resequencing efforts of individuals from the 1000 Genomes Project which is comprised of 3,202 individuals, including the original 2,504 from Phase 3 and an additional 798 relatives. The balance threshold was set to ±0.1. CD3^+^ healthy cells were used as a negative control, and the Myla cell line was used as a positive control for WGS. The threshold for CNA evaluation was set to ±0.1 log_2_ ratio, where +0.1 represents gain and −0.1 loss.

### Oncomine data extraction and linear regression analysis

Expression data were extracted from the Oncomine™ Platform (Thermo Fisher Scientific) Caprini Lymphoma dataset, which includes 32 Sezary syndrome patients (Caprini *et al*, 2009; www.oncomine.org). In the study, the CD3^+^ mononuclear cells from the blood of patients were subjected to the Affymetrix HG‐U133A assay. We extracted CD3^+^ clonality and genetic data, namely information on 17q status, from the original study. Only patients with 17q gains were included in the analysis. Spearman correlation analysis (non‐parametric) was used to correlate the percentage of clonal CD3^+^ cells and the respective *STAT3/5A/5B* expression values to obtain the *r* and *P*‐values. Simple linear regression was used to plot the graph using GraphPad Prism 8.4.2 software.

### Cell culture of established and primary cells

Hut78, Myla, SeAx, H9, Mac1, and HH established CTCL cell lines were a kind gift from the Center for Molecular Medicine (CMMC) Cologne, Germany. Primary human fibroblasts, derived from explants cultures of pieces of the juvenile foreskin, were provided by the Medical University of Graz, with the approval of the Ethics committee of the Medical University of Graz, dated 04.09.2019 (under the identification code: 31‐528 ex 18/19). HaCat, an established keratinocyte cell line, was purchased from the American Type Culture Collection (ATCC, USA). The authenticity of the CTCL cell lines and HaCaT cell line was confirmed by short tandem repeat (STR) profiling (Microsynth GmbH, Austria). All established cell lines were immediately expanded after receiving, and stocks of each cell line were prepared within 3–5 passages and stored in liquid nitrogen. Cell lines were regularly tested for *Mycoplasma* using the MycoAlertTM mycoplasma detection kit (Lonza Group AG, Switzerland), and only negative cells were used. Cell lines were used for fewer than 2 months after resuscitation. Primary fibroblast cells and keratinocytes were only used for one experiment repeated in parallel in triplicates since they lose their inherent characteristics upon longer cultivation. All cell lines were cultivated at 37°C in a humidified atmosphere containing 5% CO_2_. Hut78, Myla, SeAx, H9, Mac1, and HH cells were grown in RPMI1640 (Sigma‐Aldrich, USA) supplemented with 10% fetal bovine serum (FBS; Thermo Fisher Scientific), 2 mM L‐glutamine (Thermo Fisher Scientific), and 1% penicillin/streptomycin (Thermo Fisher Scientific). Media of SeAx cells received 5 ng/ml of interleukin (IL)‐2 and IL‐4 (ImmunoTools GmbH, Germany). Primary fibroblast and HaCat cells were grown in Dulbecco's modified Eagle's medium (DMEM; Life Technologies, USA) supplemented with 10% FBS, 2 mM L‐glutamine (Thermo Fisher Scientific), and 1% penicillin/streptomycin (Thermo Fisher Scientific).

### Oligonucleotide array comparative genomic hybridization (aCGH)

DNA was isolated from the CTCL cell lines using the QiaAmp DNA Blood Mini kit (Qiagen). Normal human reference DNA from multiple anonymous male donors was used as the reference DNA (Promega). Labeling and hybridization procedures were performed using the SureTag DNA Labelling kit, according to instructions (Agilent Technologies, USA). Equal quantities of fluorescently labeled DNA were mixed and co‐hybridized to a 44 K DNA microarray (G4426B‐014950, Agilent Technologies). After hybridization and washing, according to the manufacturer's protocol, scanning was performed on a G2505B Micro Array Scanner (Agilent Technologies). Feature extraction and data analysis were carried out using Feature Extraction (version 10.7.3.1) and Agilent Genomic Workbench version 7, respectively (Agilent Technologies). The ADM‐1 algorithm and a threshold of 6 were applied, and borders for aberrations were set to ±0.25 (log_2_ ratio) with a minimum number of three probes per region (default settings recommended by Agilent Technologies).

### Cell viability assays

Approximately, 10,000 cells/well were seeded in triplicates on flat‐bottom 96‐well plates (Greiner AG, Austria). The following day, cells were treated with serial twofold dilutions of the drug of interest, or DMSO, used as a negative control. Bortezomib, a proteasome blocker, was used as a positive control. Treated cells were incubated at 37°C and 5% CO_2_ for 48 h. Cell viability was measured using the CellTiter‐Glo Luminescent Cell Viability Assay (Promega) or the CellTiter‐Blue® Cell Viability Assay (Promega) on the GloMax® Discover Microplate Reader (Promega) or the LUMIstar Galaxy (BMG Technologies, USA). IC_50_ values were calculated using non‐linear regression of log‐transformed, normalized data. Due to a lack of material, the viability experiment with primary cells was only done once in technical triplicates.

### Immunoblot analysis

Sample preparation and Western blotting were performed using standard techniques. Nitrocellulose membranes (0.45 μm Amersham Protran 10600002, GE Healthcare, UK) were blocked in Odyssey Blocking Buffer (LI‐COR Biosciences, USA) and incubated with the respective antibody diluted in the same buffer. Information about the antibodies and their dilutions used for Western blotting are available in Dataset EV6. Drugs, primers, and antibodies used. Images were obtained with Odyssey Licor (LI‐COR Biosciences).

### Real‐time apoptosis/necrosis assay

Detection reagents were prepared according to the manufacturer's instructions (Promega) and added to the different culture media after adding the required cytokines and drugs. Luminescence and fluorescence were measured every 3–6 h after adding the substrates using a GloMax plate reader (Promega). Wells with only media served as background control, DMSO was used as negative control, while a proteasome blocker, bortezomib, was used as positive control. The relative luminescence units (RLU) reflect the binding of Annexin V to phosphatidylserine expressed on the cell surface in early apoptosis, while relative fluorescence units (RFU) represent the binding of a fluorescent dye to DNA upon membrane dissociation in secondary necrosis.

### Thermal shift assay

Thermal shift assays were performed using a C1000 Touch ThermoCycler equipped with a CFX96 Real‐Time optical unit (Bio‐Rad, USA). Final concentrations of the sample components included 2 μM STAT5B, 5× SYPRO Orange protein gel stain (Sigma‐Aldrich), in the presence or absence of 100 μM ligand (peptide [Ac‐QDTpYLVLDKWL] or IQDMA) in 100 mM HEPES, pH 7.4, and 5% (v/v) DMSO. Samples were incubated for 10 min before the melting curve experiments, which involved incrementally increasing the temperature from 25 to 75°C in 0.5°C steps, with 30 s equilibration time at each temperature. The fluorescence intensity at each temperature was recorded at 510–530 nm, following excitation at 450–490 nm. The emission intensity was utilized to generate a negative first derivative plot. The temperature for the maxima was determined, which corresponds to the melting temperature of the protein. All experiments were repeated in technical triplicates in two independent trials.

### Fluorescence polarization assay

Fluorescence polarization assays were performed with 180 nM STAT5B, 20 nM fluorescent peptide (FAM‐GpYLVLDKW), and increasing concentrations of ligand (unlabeled peptide [Ac‐QDTpYLVLDKWL] or IQDMA) in 100 mM HEPES pH 7.4, 1 mM EDTA, 1 mM DTT, and 0.01% DMSO. STAT5B was pre‐incubated with each ligand for 1 h, followed by the addition of the fluorescent peptide probe and an additional 10 min incubation prior to FP measurement. Polarization measurements were collected using an Infinite M1000‐Tecan (excitation/emission = 470 nm/530 nm), and the data were fitted using Prism GraphPad 6 and the built‐in function, log(inhibitor) vs. response–variable slope (four parameters). The experiments were performed in biological triplicates.

### Kinome screen

IQDMA was assessed at 10 μM through kinome profiling using the scanEDGE screening platform of 97 kinases (Eurofins Scientific/DiscoverX, Luxembourg). The kinome trees were generated through the online visualization tool Coral.

### Subcellular fractionation

After 16 h incubation with the drug, cells were washed once with PBS, and seeded in media with the fresh drug without additional IL‐2/‐4. Cells were stimulated for 30 min with 1 ng/ml IL‐2 and harvested after 8 h (total drug treatment: 24 h) and washed twice with PBS. To separate the nucleus from the cytoplasmic fraction, cells were resuspended in cytoplasmic buffer [10 mM HEPES (pH 7.9), 10 mM KCl, 0.1 mM EDTA, 0.1 mM EGTA, 2 mM DTT, 0.4 mM sodium orthovanadate, 25 mM sodium fluoride, and 1 mM PMSF] and incubated on ice for 15 min. NP‐40 was added to a final concentration of 0.6%, and the cell suspension was gently vortexed. The cytoplasmic supernatant was collected by centrifugation at 13,000 *g* for 60 s at 4°C. Nuclear pellets were washed three times with PBS, vortexed for 5 min at 4°C, and resuspended in nuclear buffer [20 mM HEPES (pH 7.9), 25% glycerol, 400 mM NaCl, 1 mM EDTA, 1 mM EGTA, 2 mM DTT, 0.4 mM sodium orthovanadate, 25 mM sodium fluoride, and 1 mM PMSF] and vigorously shaken at 4°C for 30 min. The nuclear fraction was lysed with a probe sonicator, incubated on ice, and vortexed on the highest setting for 10 s every 5 min for a total time of 30 min. The nuclear fraction was collected by centrifugation (13,000 *g*) for 5 min. Western blotting was performed according to described procedure herein.

### Global proteomics

SeAx cells were treated with cytokines (5 ng/ml IL‐2/4) and with DMSO, 1 μM IQDMA, 2.5 μM IQDMA, or 10 μM IQDMA in biological triplicates (DMSO, 1 μM IQDMA, and 2.5 μM IQDMA) or biological duplicate (2.5 μM IQDMA) for 24 h, and cells were harvested by centrifugation. Additionally, 4 h before collection, 7.5 ng/ml of IL‐2/4 was added. Lysis buffer (8 M urea, 1× cOmplete™, Mini, EDTA‐free protease inhibitor (Roche Holding AG, Switzerland), in MilliQ water (MQ)) was added to the cell pellets and samples were homogenized using sonication with a probe sonicator (15 × 0.5 s pulses) at 4°C. The homogenized sample was clarified by centrifugation at 20,000 × *g* for 10 min at 4°C. A BCA assay (Thermo Fisher Scientific) was used to determine the final protein concentration in the cell lysate. Two hundred microgram protein for each sample was transferred to new tubes. The proteins were reduced by DTT (5 mM DTT, 30 min, and 37°C), then alkylated by iodoacetamide (15 mM iodoacetamide, 30 min, room temperature). Thereafter, proteins were precipitated by acetone precipitation. Briefly, four volumes of cold acetone were added to samples, and the tubes were incubated for 90 min at −20°C. The mixture was centrifuged at 15,000 × *g* for 7 min to precipitate the proteins. The protein pellets were washed twice with 1 ml of acetone, centrifuged at 15,000 × *g* for 5 min, and the resulting washed pellets were allowed to air dry. The pellets were resuspended in 8 M urea in MQ, followed by a second BCA assay to determine the final protein concentration in the samples. Ten microgram protein for each sample was transferred to new tubes. Urea concentration was diluted to 1 M with the addition of 100 mM triethylammonium bicarbonate (TEAB) pH 8.5 for digestion with trypsin/LysC mixture (1:50; enzyme:protein) for 12 h at 37°C. Tandem mass tag (TMT) reagents (Thermo Fisher Scientific) dissolved in anhydrous acetonitrile (ACN) were added to samples according to the manufacturer's instructions. The 11‐plex labeling reactions were performed for 1 h at room temperature and the reaction was quenched by the addition of 0.5% hydroxylamine for 15 min at room temperature. Samples were then combined and dried down in a speed vacuum and were fractionated into eight fractions using a high pH reversed‐phase peptide fractionation kit (Pierce, Thermo Fisher Scientific). The resulting fractions were dried down by speed vacuum, resuspended in 1% formic acid solution, and analyzed by mass spectrometry.

Data were collected using an Orbitrap Fusion Lumos mass spectrometer (Thermo Fisher Scientific) coupled with a Eksigent ekspert™ nanoLC 425 (Sciex, USA) using a SPS‐MS3 protocol. Peptides were eluted by a gradient at 200 nl/min from 2.5% acetonitrile with 0.1% formic acid to 35% acetonitrile with 0.1% formic acid using a linear gradient of 120 min. This was followed by an 8 min gradient from 35% acetonitrile with 0.1% formic acid to 80% acetonitrile with 0.1% formic acid. Next, there was an 8 min wash with 80% acetonitrile with 0.1% formic acid, and equilibration for another 23 min to 2.5% acetonitrile with 0.1% formic acid. The MS1 scan was within a mass range 400–1,600 Da, using orbitrap resolution of 120,000, 30% RF lens, and 2,400 volts. This was followed by MS/MS scans with a total cycle time of 2.5 s and the first mass of 120 m/z. An accumulation time of 40 ms and 32% HCD collision energy was used for each MS/MS scan. Each candidate ion was required to have a charge state from 2 to 6, fragmented with 35% CID collision energy and an accumulation time of 10 ms, using orbitrap resolution of 15,000. Previously analyzed candidate ions were dynamically excluded for 15 s. Isobaric Tag loss exclusion used was TMT. This was followed by six MS3 events. The scan range was from 100 to 500 m/z, using an orbitrap resolution of 50,000 and HCD collision energy of 55%. All ions were required to be at 300% AGC target.

PEAKS Scientific XPro (Bioinformatics Solutions, Canada) was used for data processing. MS/MS spectra were searched against a UniProt human database with both the forward and reverse sequences. Database search criteria are as follows: tryptic with two missed cleavages, a precursor mass tolerance of 20 ppm, and fragment ion mass tolerance of 0.2 Da. TMT labeling of lysine residues and N‐termini of peptides (229.16293 Da), and alkylation of cysteine (57.02146 Da) were selected as fixed modifications, and oxidation of methionine (15.99491 Da) and deamidation (+0.9840) were selected as variable modifications. Only unique peptides with an identification false discovery rate of 2% were selected for TMT reporter ion‐based quantification. Statistical analysis was carried out using the limma package within the R framework (Ritchie *et al*, 2015).

### Drug synergy screen

The analysis was performed using the SynergyFinder 2.0 web application (Ianevski *et al*, 2020). The degree of synergy was quantified using the zero‐interaction potency (ZIP) model that captures the drug interaction relationships by comparing the change in the potency of the dose–response curves between individual drugs and their combinations (Yadav *et al*, 2015). The ZIP model enabled the calculation of the most synergistic (MSA) area score, which represents the most synergistic three‐by‐three dose window. The zero‐interaction potency model was applied to quantify the degree of synergy, according to which an MSA score below −10 indicates that drugs are antagonistic, a score between −10 and 10 indicates that two drugs are additive, while a score above 10 indicates a synergistic effect. The four‐parameter logistic regression (LL4) was used as the curve‐fitting algorithm (Ianevski *et al*, 2020). All assays were repeated in two biological replicates.

### *In vivo* intradermal xenograft model

Animal work was done in accordance with institutional guidelines on animal welfare, with the approval of the Federal Ministry of Science, Research and Economy of Austria (BMBWF‐68.205/0130). A total of 17 male and 27 female NOD scid gamma (NSG‐SGM3) mice (RRID: IMSR_JAX:013062) were ordered from the Charles River Laboratories, Germany GmbH. Mice were maintained under pathogen‐free conditions in individually ventilated cages at the University of Veterinary Medicine Vienna. Mice were kept at 12/12 h light cycle and received standard food and water *ad libitum*. Mice were shaved on their back before the tumor cell injection and randomized into three different grafting groups based on sex and age (*n* = 15 mice per group). Under isoflurane inhalation anesthesia (1–1.5% in O_2_, 0.5 l/min), 1 × 10^6^ Hut78 and Myla cells, and 3 × 10^6^ H9 cells in 100 ml of PBS, were intradermally injected into the back skin of the mouse. A total of 30 injections were performed per cell line. The H9 group was not included further due to the low tumor engraftment rate. Hut78 and Myla post‐injection mice were randomized into five treatment groups based on tumor cell engraftment, once every mouse in the respective cell group showed at least one visible tumor. Tumor growth was assessed every 2–3 days using Vernier calipers and tumor volumes were calculated with the following formula: tumor volume = (length × width^2^)/2. Mice were intraperitoneally treated with 5 mg/kg of JPX‐0750 and IQDMA or vehicle (5% DMSO, 50% PEG‐400, 5% TWEEN®80, 40% H_2_O), and with 5 mg/kg FRAx597 or vehicle (10% DMSO, 5% Kolliphor EL, 30% Propylene glycol, 55% H_2_O), until the control group tumors reached approximately 1 cm^3^. The animals were euthanized by cervical dislocation and the tumors and organs were resected and used for further analysis.

### Hematocytometry and flow cytometry

Blood from mice was obtained by heart puncture and collected in EDTA tubes (Mini‐Collect K3EDTA tubes, Greiner AG). WBC count and hematocrit were measured using an animal blood counter (sciI Vet abc, Germany). For flow cytometry, erythrocytes were lysed using Gay's solution (10 mM KHCO_3_ and 75 mM NH_4_Cl, pH 7.4). Flow cytometry was used to determine the percentage of human CD45^+^ cells in the blood, lymph nodes, liver, and kidney (CD45 Monoclonal Antibody (HI30), APC, #17‐0459‐42, Thermo Fisher Scientific). Single‐cell suspensions were prepared by mincing organs through a 70 μm cell strainer (BD Biosciences, USA). All analyses were performed on a BD FACS Canto II™ instrument and calculated with FACSDiva software (v8.0.1, BD Biosciences).

### Histopathology and immunohistochemistry

Intradermal tumors were carefully harvested and dissected lengthwise into two equal halves using a surgical scalpel. Tumors were fixed overnight in 4% phosphate‐buffered formaldehyde solution (Roti® Histofix; Carl Roth, Germany), dehydrated, paraffin embedded, and cut. Consecutive mouse organs or tumor 3‐μm‐FFPE sections were stained with Hematoxylin (Merck, Germany) and Eosin G (Carl Roth). For immunohistochemical stainings, heat‐mediated antigen retrieval was performed in citrate buffer at pH 6.0 (S1699; Dako, Agilent Technologies) and sections were stained with antibodies against Ki67 (#KI67‐MM1‐L‐CE, Leica Biosystems), and cleaved caspase 3 (#9664, CST) and CD31 (#77699, CST) using standard protocols. The H&E sections were scanned using ScanScope®XT system (Leica Biosystems). The H&E staining was analyzed and annotated using QuPath software (Version 0.3.2). Infiltration/expansion of tumor cells in the intradermal and subcutaneous regions of the skin was annotated. The area and perimeter of the annotated infiltration region per tumor were measured and plotted as violin plots using GraphPad prism 8.0. Statistical significance was calculated using an unpaired *t*‐test with Welch's correction due to unequal SD. The Ki67 and CC3 staining images were taken with a Zeiss AxioImager Z1 microscope and five randomly selected areas per slide were quantified using the QuPath software (Version 0.3.2). Tumor vessel formation was assessed by CD31 staining. The CD31 staining was scanned using the Panoramic MIDI slide scanner (3DHistech Ltd.) with 40× optics. Quantitation was performed using Definiens TM Tissue Studio histomorphometry software (Definiens AG, Germany).

### Statistical analysis

GraphPad Prism 7 and 8 were used to perform the statistical analyses. Statistical tests used are specified in Figure legends. The threshold for statistical significance was set to *P* < 0.05, unless otherwise specified. *P*‐value: < 0.05 (*), < 0.01 (**), < 0.001 (***), and < 0.0001 (****).

### Graphical licenses

The graphical abstract and the mouse experiment schematics are created using BioRender.com, under the agreement number: OQ24G9CYP2 for the graphical abstract and LO2455YNMK for the mouse experiment scheme.

## Author contributions

**Helena Sorger:** Conceptualization; data curation; software; formal analysis; validation; investigation; visualization; methodology; writing – original draft; project administration. **Saptaswa Dey:** Conceptualization; data curation; software; formal analysis; validation; investigation; visualization; methodology; writing – original draft; project administration. **Pablo Augusto Vieyra‐Garcia:** Formal analysis; methodology; writing – review and editing. **Daniel Pölöske:** Formal analysis; methodology. **Andrea R Teufelberger:** Software; formal analysis; methodology. **Elvin D de Araujo:** Software; formal analysis; methodology; writing – original draft. **Abootaleb Sedighi:** Software; formal analysis; methodology. **Ricarda Graf:** Software; formal analysis; methodology. **Benjamin Spiegl:** Software; formal analysis; methodology. **Isaac Lazzeri:** Software; formal analysis; methodology. **Till Braun:** Patient sample collection and characterization. **Ines Garces de los Fayos Alonso:** Formal analysis; methodology. **Michaela Schlederer:** Methodology. **Gerald Timelthaler:** Methodology. **Petra Kodajova:** Methodology. **Christine Pirker:** Software; formal analysis; methodology. **Marta Surbek:** Formal analysis; methodology. **Michael Machtinger:** Methodology. **Thomas Graier:** Methodology. **Isabella Perchthaler:** Methodology. **Yi Pan:** Methodology. **Regina Fink‐Puches:** Patient sample collection and characterization. **Lorenzo Cerroni:** Patient sample collection and characterization. **Jennifer Ober:** Methodology. **Moritz Otte:** Patient sample collection and characterization. **Jana D Albrecht:** Patient sample collection and characterization. **Gary Tin:** Methodology. **Ayah Abdeldayem:** Methodology. **Pimyupa Manaswiyoungkul:** Methodology. **Olasunkanmi O Olaoye:** Methodology. **Martin L Metzelder:** Methodology. **Anna Orlova:** Formal analysis; methodology. **Walter Berger:** Writing – review and editing. **Marion Wobser:** Patient sample collection and characterization. **Jan P Nicolay:** Patient sample collection and characterization; Writing – review and editing. **Fiona Andre:** Patient sample collection and characterization. **Van Anh Nguyen:** Patient sample collection and characterization; Writing – review and editing. **Heidi A Neubauer:** Conceptualization; validation; writing – review and editing. **Roman Fleck:** Methodology. **Olaf Merkel:** Writing – review and editing. **Marco Herling:** Patient sample collection and characterization; conceptualization; validation; writing – review and editing. **Ellen Heitzer:** Resources; software; validation; writing – review and editing. **Patrick T Gunning:** Conceptualization; resources; supervision; funding acquisition; project administration. **Lukas Kenner:** Conceptualization; resources; supervision; funding acquisition; validation; project administration. **Richard Moriggl:** Conceptualization; resources; supervision; funding acquisition; validation; writing – original draft; project administration; writing – review and editing. **Peter Wolf:** Conceptualization; resources; supervision; funding acquisition; validation; project administration; writing – review and editing.

## Disclosure and competing interests statement

The authors declare that they have no conflict of interest.

## Supporting information



Appendix

Expanded View Figures PDF

Table EV1

Table EV2

Table EV3

Table EV4

Table EV5

Table EV6

Dataset EV1

Dataset EV2

Dataset EV3

Dataset EV4

Dataset EV5

Dataset EV6

Source Data for Expanded View and Appendix

Source Data for Figure 2

Source Data for Figure 3

Source Data for Figure 4

Source Data for Figure 5

Source Data for Figure 6

Source Data for Figure 7

PDF+

## Data Availability

The raw sequencing data (WES and sWGS) are available from the European Genome‐Phenome Archive (EGA) database under the accession code EGAS00001004719 (https://ega-archive.org/studies/EGAS00001004719). Proteomics data were deposited to the PRIDE database under the accession number: PXD035626 (https://www.ebi.ac.uk/pride/archive/projects/PXD035699).
