# Supplementary material for: Blocking STAT3/5 through direct or upstream kinase targeting in leukemic cutaneous T‐cell lymphoma
Source: EMBO Mol Med. 2022 Nov 7;14(12):e15200. doi: 10.15252/emmm.202115200 (PMC9727928; doi:10.15252/emmm.202115200)
Supplement: Supplementary file 1 — Appendix [file EMMM-14-e15200-s005.pdf]

APPENDIX

Blocking STAT3/5 through direct or upstream kinase targeting in leukemic cutaneous T-cell lymphoma

Table of contents

Appendix Figure S1. Identification of the TCRVβ+ malignant clonal population in L-CTCL patients. 2

Appendix Figure S2. L-CTCL patients present with 17q gains 4

Appendix Figure S3. Malignant T-cells infiltrating the skin of L-CTCL patients are CD3+ STAT3+ STAT5A+ STAT5B+ pY-STAT5+ 6

Appendix Figure S4. Established cell lines can be employed as in vitro models for L-CTCL. 8

Appendix Figure S5. The PAK kinase inhibitor strongly synergizes with JPX-0750 and IQDMA, as well as ponatinib and ceritinib 10

Appendix Figure S6. The selected drugs, JPX-0750, IQDMA and FRAx597 are safe to use in vivo..12

Appendix Table S1. P-value summary from Figure EV2E and Figure 4H-I. 13

Appendix Figure S1

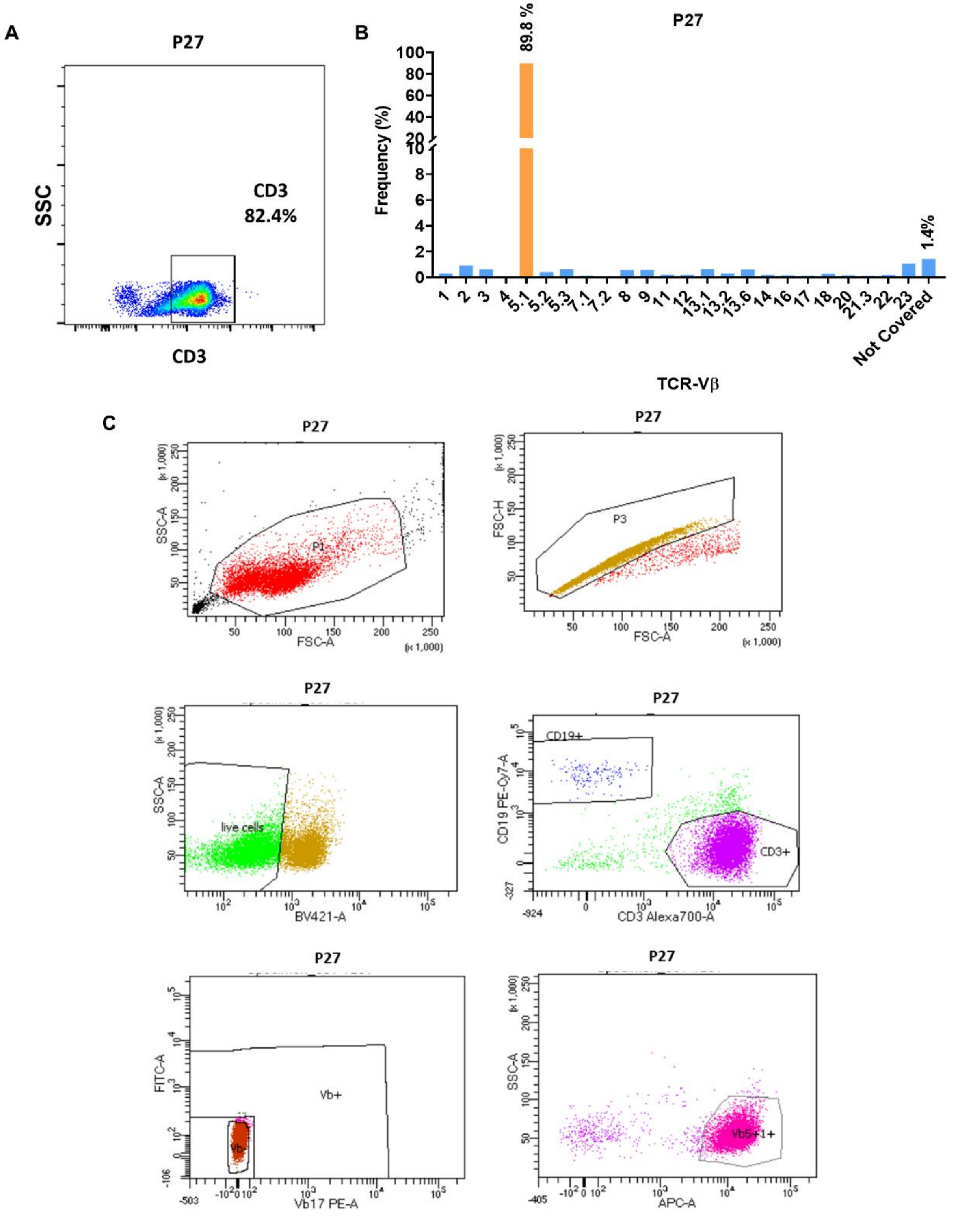

Appendix Figure S1. Identification of the TCRVβ+ malignant clonal population in L-CTCL patients.

A-C Patient PBMCs are subjected to live/dead staining, CD3 staining and TCRVβ staining, a multi-parametric analysis tool designed for quantitative determination of the TCRVβ repertoire of human T lymphocytes by flow cytometry. (A) representative image of the CD3 gating strategy. (B) TCRVβ clonal frequency analysis plot. A dominant clonal population (TCRVβ 5.1) is identified from P27, shown as representative. (C) A representative figure from P27 showing the flow cytometric gating strategy for sorting/enrichment of the CD3+ TCRVβ+ clonal population.

Appendix Figure S2

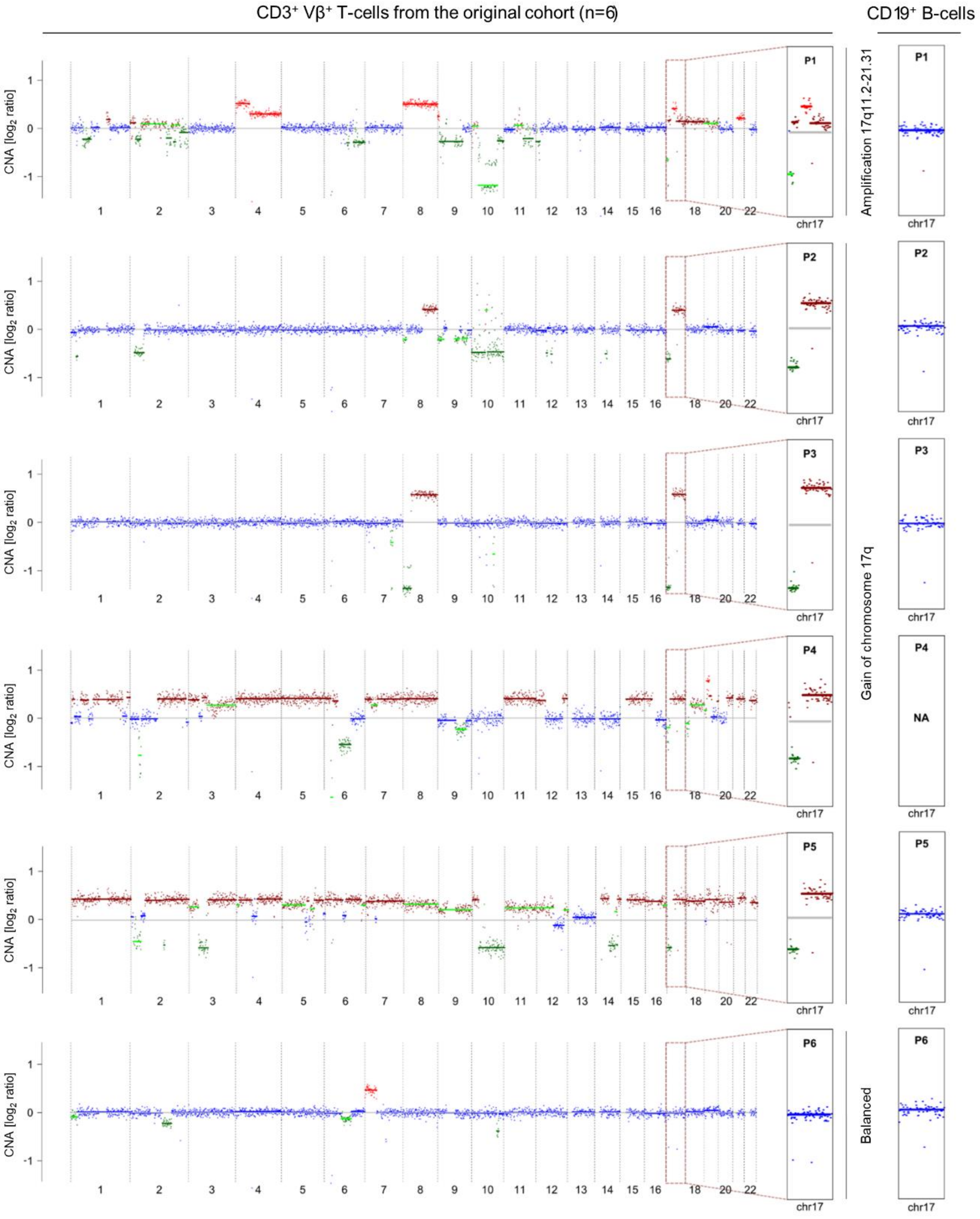

Appendix Figure S2. L-CTCL patients present with 17q gains

sWGS was performed on sorted CD3+/Vβ+ malignant T-cells isolated from the blood of the patients. Copy number profiles and ploidy estimates were calculated using ichorCNA. Blue represents balanced genomic regions, green indicates loss/deletion, and red indicates gain/amplification of regions on chromosome 17. Darker color shades indicate higher log2 ratios. The copy number profile of P1 was re-used in **Figure 1A**.

Appendix Figure S3

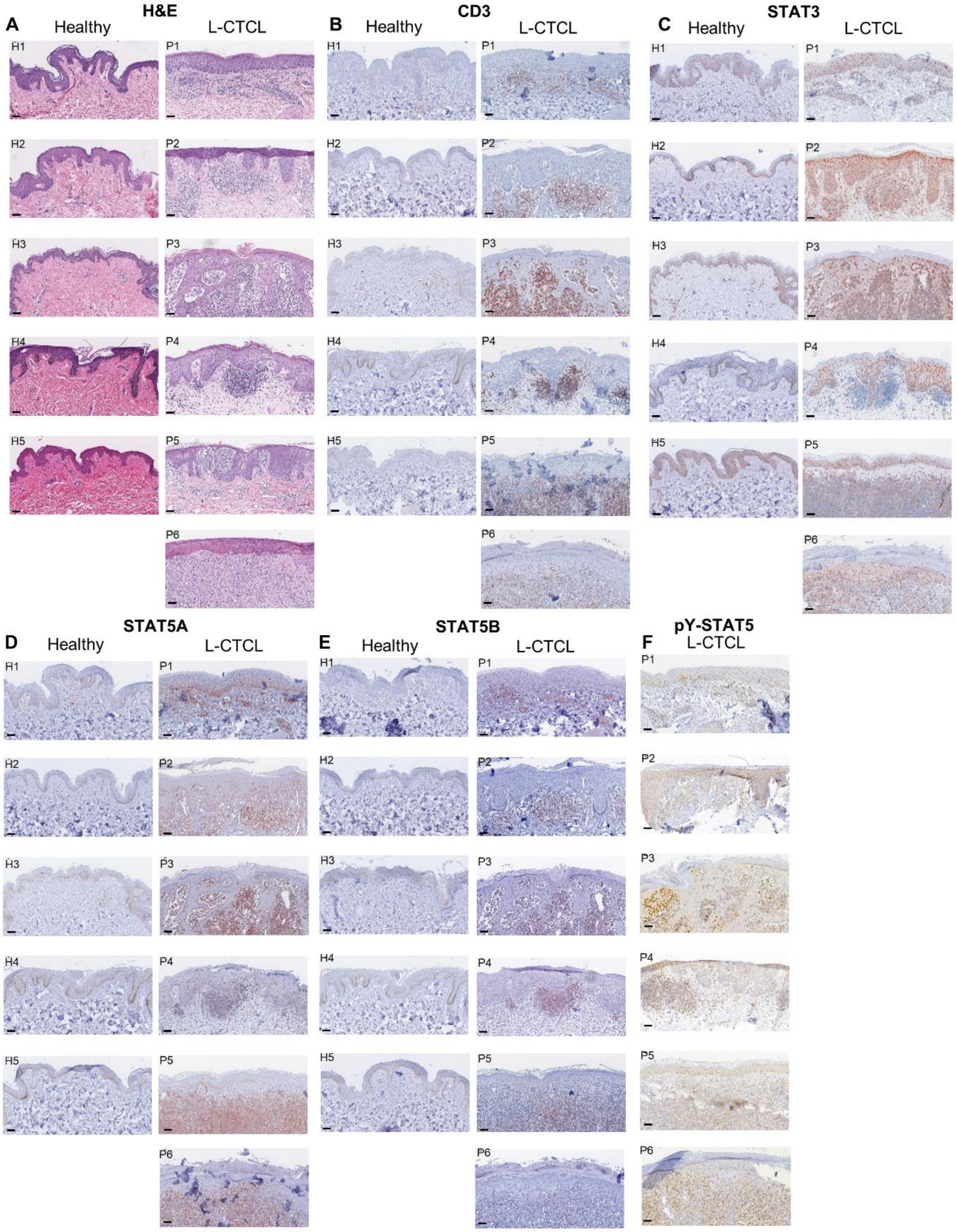

Appendix Figure S3. Malignant T-cells infiltrating the skin of L-CTCL patients are CD3+ STAT3+ STAT5A+ STAT5B+ pY-STAT5+

A-F H&E and immunohistochemical staining of skin-biopsy sections from L-CTCL patients (n=6) and healthy individuals (n=5). (A) H&E, (B) CD3, (C) STAT3, (D) STAT5A, (E) STAT5B, (F) pY-STAT5. The STAT3+/STAT5A+/STAT5B+/pY-STAT5+ cell infiltration overlays with the CD3+ cell infiltration region in the dermal and subcutaneous layer of the skin compared to healthy skin. As the number of STAT5A/B+ cells were negligible (less than 0.03/per mm2 on average) (C, D) in healthy skin biopsy sections, we did not perform pY-STAT5 staining for the healthy skin. Scale bar 50 μm. Quantification of the IHC staining is depicted in **Figure 2I-M**.

Appendix Figure S4

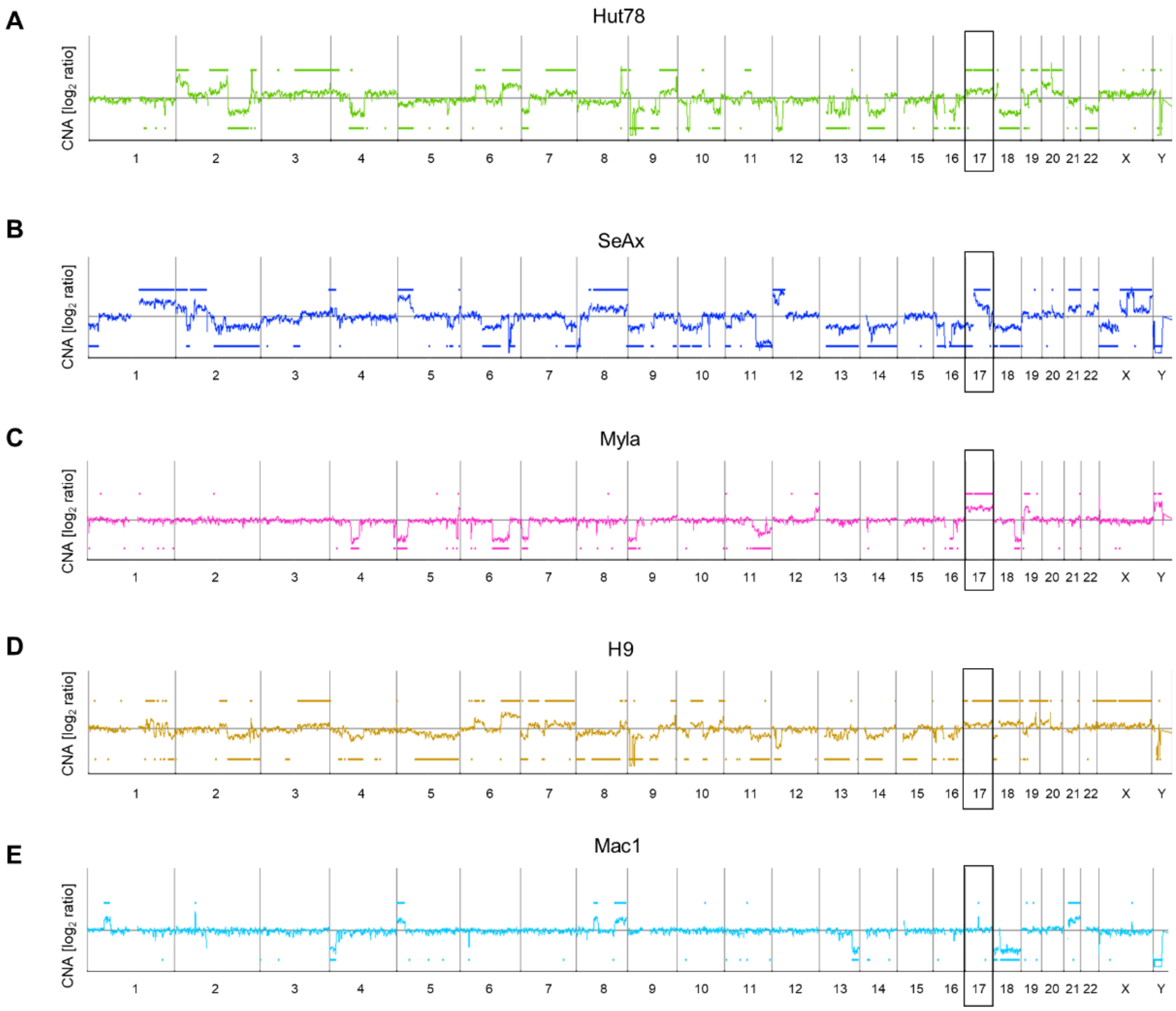

Appendix Figure S4. Established cell lines can be employed as in vitro models for L-CTCL.

A-E Chromosomal aberrations detected via aCGH plotted across each chromosome for each individual cell line. Aberration patterns are represented as the log2 ratio of the fluorescence intensity of the tumor DNA vs. reference DNA. Graphical depiction of the aberration patterns on chromosome 17 (*STAT3/5* region), chromosome 2 (*STAT1* region) and chromosome 16 (*SOCS1* region) were re-used in **Figure 3**.

Appendix Figure S5

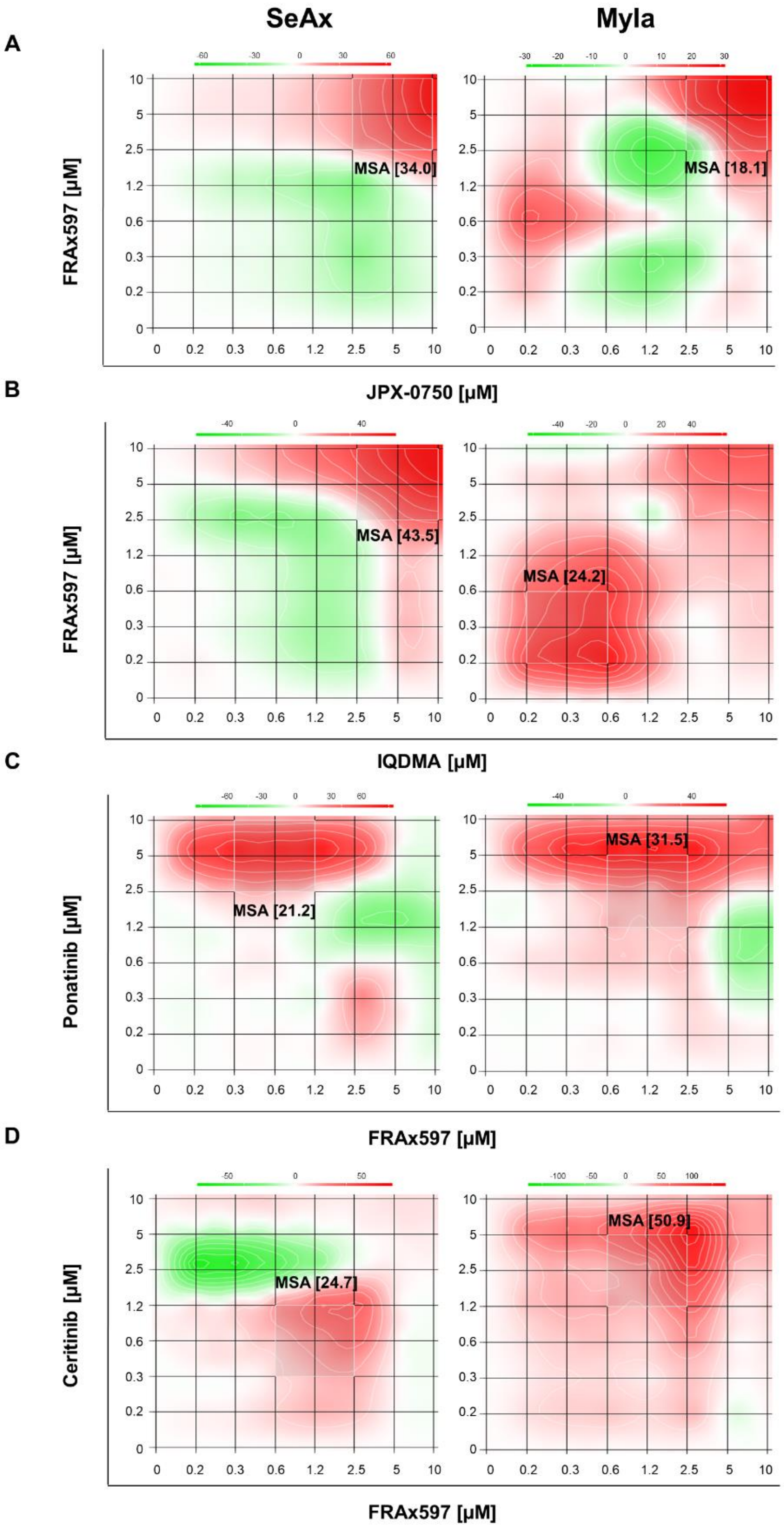

Appendix Figure S5. The PAK kinase inhibitor strongly synergizes with JPX-0750 and IQDMA, as well as ponatinib and ceritinib

A-D Synergy analysis of the indicated two-drug combinations in the SeAx and Myla cells after 48 h treatment. In each graph the most synergistic area (MSA) is highlighted, which represents the most synergistic 3-by-3 dose-window with the respective MSA score. The Zero interaction potency model was applied to quantify the degree of synergy, according to which an MSA score below -10 indicates that drugs are antagonistic (green), a score between -10 and 10 indicates that 2 drugs are additive (white), while a score above 10 indicates a synergistic effect (red). Heatmap showing MSA scores of the combination of FRAx597 with the listed drugs is represented in **Figure 6D**.

Appendix Figure S6

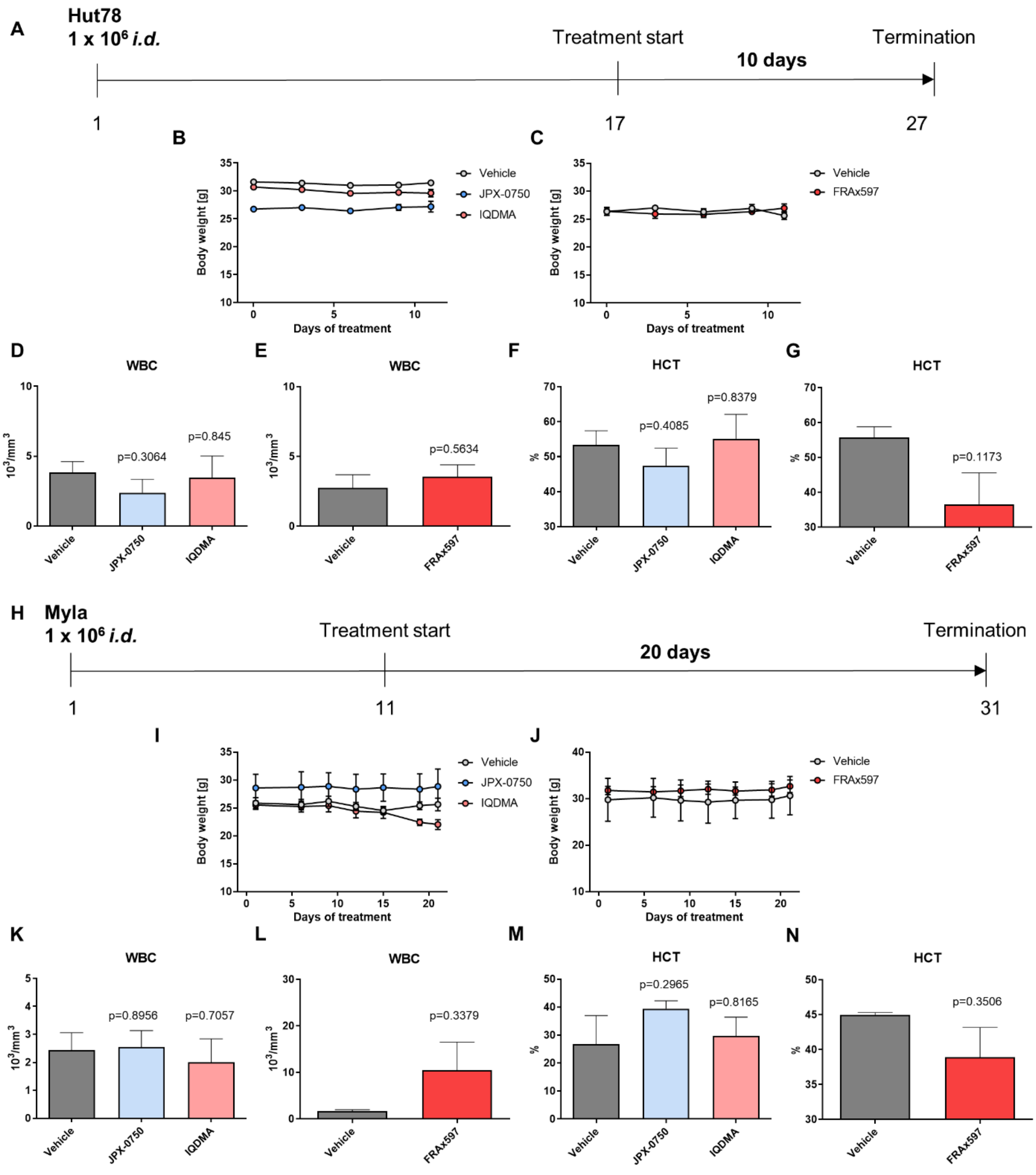

Appendix Figure S6. The selected drugs, JPX-0750, IQDMA and FRAx597 are safe to use in vivo

A In vivo experiment duration for the Hut78 cell line.

B-C Body weight changes at the respective days of treatment. Error bars represent +/-SEM.

D-E White blood cell (WBC) count upon termination. Statistical significance was calculated using two-tailed paired t-test with Welch´s correction. Error bars represent +/-SEM.

F-G Hematocrit (HCT) upon termination. Statistical significance was calculated using two-tailed paired t-test with Welch´s correction. Error bars represent +/-SEM.

H In vivo experiment duration for the Myla cell line.

I-J Body weight changes at the respective days of treatment. Error bars represent +/-SEM.

K-L White blood cell (WBC) count upon termination. Statistical significance was calculated using two-tailed paired t-test with Welch´s correction. Error bars represent +/-SEM.

M-N Hematocrit (HCT) upon termination. Statistical significance was calculated using two-tailed paired t-test with Welch´s correction. Error bars represent +/-SEM.

Appendix Table S1. P-value summary from Figure EV2E and Figure 4H-I. Statistical significance was calculated using two-way ANOVA with multiple comparisons. P-value: <0.05 (*), <0.01 (**), <0.001 (***), 0.0001 (****). *JF = juvenile foreskin.*

	Gene		Concentration [JPX-0750, µM]	p-value [in comparison to DMSO]
**Figure EV2E**	**PIM1**		1	<0.0001
			2.5	<0.0001
			5	<0.0001
	**MYC**		1	0.0008
			2.5	<0.0001
			5	<0.0001
	**CCND1**		1	0.0179
			2.5	<0.0001
			5	<0.0001
	**CCND2**		1	0.0010
			2.5	<0.0001
			5	<0.0001
	**MCL1**		1	<0.0001
			2.5	0.0007
			5	<0.0001
	**Drug**	**Concentration [µM]**	**Comparison**	**p-value**
**Figure 4H-I**	**JPX-0750**	1.563	Hut78 vs. HaCat	0.0021
			Hut78 vs. JF	0.0032
			SeAx vs. HaCat	0.0071
			SeAx vs. JF	0.0107
			Myla vs. HaCat	0.0038
			Myla vs. JF	0.0059
		3.125	Hut78 vs. HaCat	0.0204
			Hut78 vs. JF	<0.0001
			SeAx vs. JF	0.0002
			Myla vs. HaCat	0.0155
			Myla vs. JF	<0.0001
	**IQDMA**	1.563	Myla vs. HaCat	0.0041
			Myla vs. JF	0.0021
		3.125	Hut78 vs. JF	0.0026
			SeAx vs. JF	0.0063
			Myla vs. HaCat	0.0194
			Myla vs. JF	0.0006
