# Supplementary material for: Blocking STAT3/5 through direct or upstream kinase targeting in leukemic cutaneous T‐cell lymphoma
Source: EMBO Mol Med. 2022 Nov 7;14(12):e15200. doi: 10.15252/emmm.202115200 (PMC9727928; doi:10.15252/emmm.202115200)
Supplement: Supplementary file 2 — Expanded View Figures PDF [file EMMM-14-e15200-s009.pdf]

Expanded View Figures

Figure EV1. *STAT3/5* expression correlates with disease clonality.

A Graphical summary of the literature analysis on genomic CTCL studies performed in the last 10 years including larger cohorts of patients, as described in Table EV3. The number of patients carrying 17q gain, specifically of the region containing the *STAT3/5* genes (17q11.2-17q21.31) is written and depicted in black, in comparison to the total number of patients included and depicted in blue or orange. Blue color depicts Sezary syndrome patients, whereas orange depicts mycosis fungoides patients.

B–E Spearman correlation analysis using CNA $\log_2$ ratios and the percentage of clonal $CD3^+$ cells detected in patients with (B) *TP53*, (C) *MYC*, (D) *STAT1*, and (E) *SOCS1*.

F–H Spearman correlation analysis on expression data extracted from the Oncomine™ Platform as published in Caprini *et al* (2009). Patients with 17q (*STAT3/5*) gains were selected and the percentage of clonal $CD3^+$ cells was correlated with the respective *STAT3/5* gene expression for the (F) *STAT3*_208991 reporter, (G) *STAT5A*_203010 reporter, and (H) *STAT5B*_212550 reporter.

Source data are available online for this figure.

© 2022 The Authors

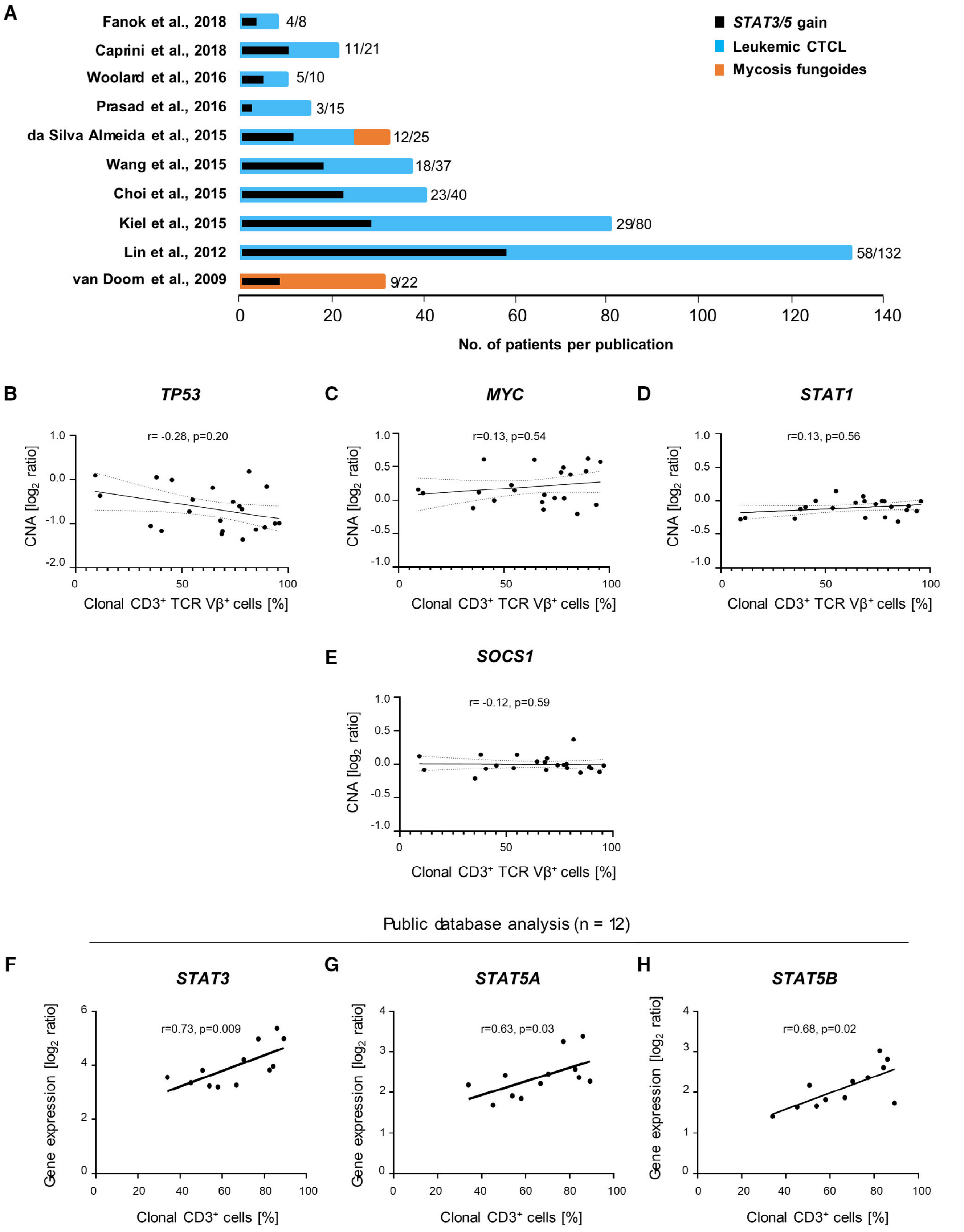
A
Fanok et al., 2018
Caprini et al., 2018
Woolard et al., 2016
Prasad et al., 2016
da Silva Almeida et al., 2015
Wang et al., 2015
Choi et al., 2015
Kiel et al., 2015
Lin et al., 2012
van Doorn et al., 2009
4/8
11/21
5/10
3/15
12/25
18/37
23/40
29/80
58/132
9/22
STAT3/5 gain
Leukemic CTCL
Mycosis fungoides
No. of patients per publication
B TP53
r= -0.28, p=0.20
C MYC
r=0.13, p=0.54
D STAT1
r=0.13, p=0.56
E SOCS1
r= -0.12, p=0.59
CNA [log2 ratio]
Clonal CD3+ TCR Vβ+ cells [%]
Public database analysis (n = 12)
F STAT3
r=0.73, p=0.009
G STAT5A
r=0.63, p=0.03
H STAT5B
r=0.68, p=0.02
Gene expression [log2 ratio]
Clonal CD3+ cells [%]

Figure EV1.

Figure EV2. JPX-0750 and IQDMA have distinct mechanisms of action.

A–D Immunoblot showing total and pY-STAT3/pY-STAT5 levels (i.e., phospho-Tyr (705)-STAT3 and phospho-Tyr (694/699)-STAT5A/B) in (A) Myla cells upon 6 h treatment with JPX-0750 and (B) Hut78 cells upon 24 h treatment with JPX-0750. (C) Myla and (D) Hut78 cells were treated with 24 h IQDMA. HSC70 was used as loading control. The normalized phospho- and total protein levels, quantified by densitometry, are shown below the respective blots. One representative out of three independent experiments is shown.

E RT–qPCR for STAT5 target genes in the SeAx cell line after 24 h treatment with JPX-0750. Gene expression was normalized to *GAPDH*. Statistical significance was calculated using two-way ANOVA with multiple comparisons. *P*-value: < 0.05 (*), < 0.01 (**), < 0.001 (***), and 0.0001 (****). *P*-value summaries are provided in Appendix Table S1. Error bars represent mean ± SD. One experiment performed in triplicates is shown.

F, G Cleaved and total PARP protein levels in Myla cells treated with (F) JPX-0750 for 6 h and (G) IQDMA for 24 h. HSC70 was used as loading control. The normalized total protein levels, quantified by densitometry, are shown below the respective blots. One representative out of two independent experiments is shown.

H, I Immunoblotting for cleaved caspase 3 levels in SeAx cells after 24 h treatment with (H) JPX-0750 and (I) IQDMA. β-Actin or HSC70 were used as loading controls. The normalized total protein levels, quantified by densitometry, are shown below the respective blots. One representative out of two independent experiments is shown.

Source data are available online for this figure.

 © 2022 The Authors

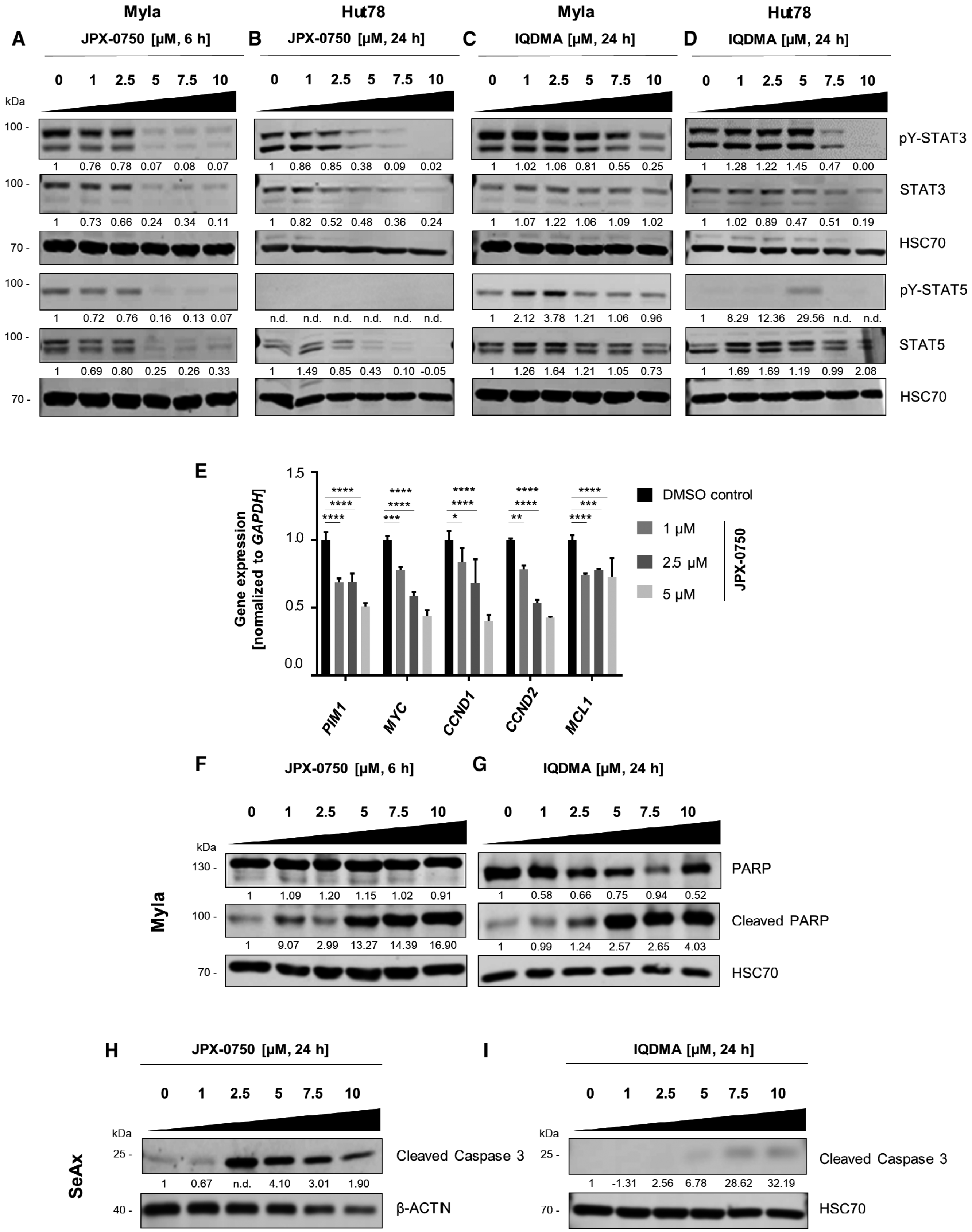

Myla
A JPX-0750 [μM, 6 h]
Hut78
B JPX-0750 [μM, 24 h]
Myla
C IQDMA [μM, 24 h]
Hut78
D IQDMA [μM, 24 h]
0 1 2.5 5 7.5 10
kDa
pY-STAT3
1 0.76 0.78 0.07 0.08 0.07
1 0.86 0.85 0.38 0.09 0.02
1 1.02 1.06 0.81 0.55 0.25
1 1.28 1.22 1.45 0.47 0.00
STAT3
1 0.73 0.66 0.24 0.34 0.11
1 0.82 0.52 0.48 0.36 0.24
1 1.07 1.22 1.06 1.09 1.02
1 1.02 0.89 0.47 0.51 0.19
HSC70
pY-STAT5
1 0.72 0.76 0.16 0.13 0.07
n.d. n.d. n.d. n.d. n.d. n.d.
1 2.12 3.78 1.21 1.06 0.96
1 8.29 12.36 29.56 n.d. n.d.
STAT5
1 0.69 0.80 0.25 0.26 0.33
1 1.49 0.85 0.43 0.10 -0.05
1 1.26 1.64 1.21 1.05 0.73
1 1.69 1.69 1.19 0.99 2.08
HSC70
E
Gene expression [normalized to GAPDH]
1.5 1.0 0.5 0.0
DMSO control
1 μM
2.5 μM
5 μM
JPX-0750
PIM1 MYC CCND1 CCND2 MCL1
F JPX-0750 [μM, 6 h]
G IQDMA [μM, 24 h]
Myla
PARP
1 1.09 1.20 1.15 1.02 0.91
1 0.58 0.66 0.75 0.94 0.52
Cleaved PARP
1 9.07 2.99 13.27 14.39 16.90
1 0.99 1.24 2.57 2.65 4.03
HSC70
H JPX-0750 [μM, 24 h]
I IQDMA [μM, 24 h]
SeAx
Cleaved Caspase 3
1 0.67 n.d. 4.10 3.01 1.90
1 -1.31 2.56 6.78 28.62 32.19
β-ACTIN
HSC70

Figure EV2.

Figure EV3. In-depth analysis of IQDMA shows its multi-kinase inhibitory function.

A Thermal shift profiles of 2 μM STAT5B with 100 μM IQDMA or 100 μM STAT5-binding peptide (positive control). The local maxima indicate the melt temperature of the protein ($T_m \pm 0.3°C$). One representative out of two independent experiments performed in technical triplicates is shown.
B Fluorescence polarization assay using 180 nM STAT5B with 100 μM IQDMA or 100 μM peptide (positive control). The positive control exhibits a single-site displacement profile. Error bars represent mean ± SD. Three independent experiments were performed.
C IQDMA structure.
D Subcellular fractions of SeAx cells treated with IQDMA for 18 h and immunoblotted for pY-STAT3 (phospho-Tyr (705)-STAT3) and total STAT3. The cells were collected 30 min after 5 ng/ml IL-2 cytokine addition. α-Tubulin and histone H3 were used as loading controls for cytoplasmic and nuclear fractions, respectively. The normalized levels of phospho- and total STAT3 in the nucleus and cytoplasm, quantified by densitometry, are shown below the respective blots. One experiment was performed.
E Bar graph showing the cellular pathways that are most affected by IQDMA treatment as determined by network enrichment analysis.
F Subcellular fractions of SeAx cells treated with FRAx597 for 18 h and immunoblotted for pY-STAT3/pY-STAT5 levels (i.e., phospho-Tyr (705)-STAT3 and phospho-Tyr (694/699)-STAT5A/B) and total STAT3/5 levels. The cells were collected 30 min after 5 ng/ml IL-2 cytokine addition. α-Tubulin and histone H3 were used as loading controls for cytoplasmic and nuclear fractions, respectively. The normalized levels of phospho- and total STAT3/5 in the nucleus and cytoplasm, quantified by densitometry, are shown below the respective blots. One experiment was performed.

Source data are available online for this figure.

 © 2022 The Authors

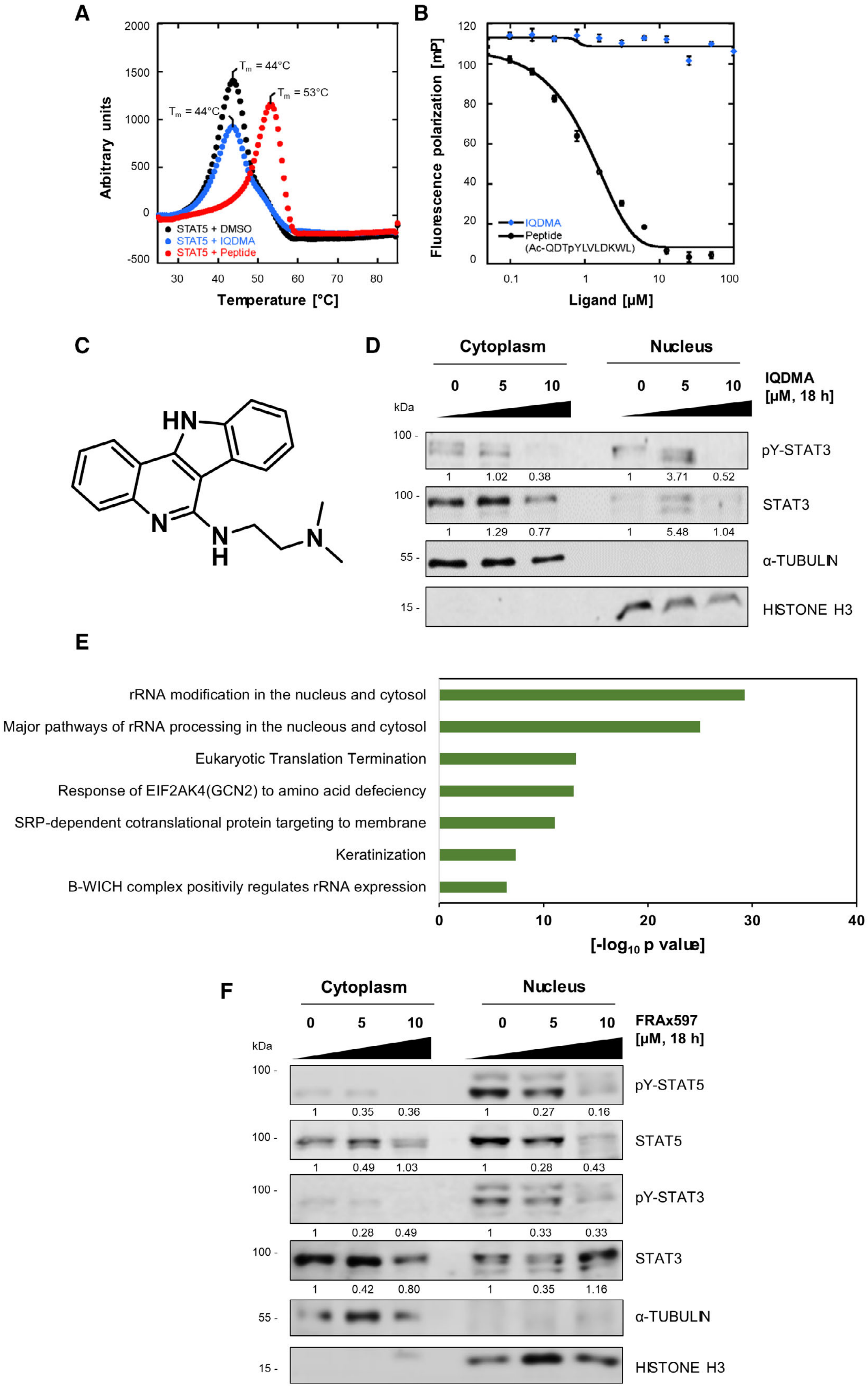
A
Arbitrary units
Temperature [°C]
T_m = 44°C
T_m = 44°C
T_m = 53°C
STAT5 + DMSO
STAT5 + IQDMA
STAT5 + Peptide
B
Fluorescence polarization [mP]
Ligand [µM]
IQDMA
Peptide
(Ac-QDTpYLVLDKWL)
C
HN
N
N
H
N
D
Cytoplasm
Nucleus
IQDMA
[µM, 18 h]
kDa
pY-STAT3
STAT3
α-TUBULIN
HISTONE H3
1 1.02 0.38 1 3.71 0.52
1 1.29 0.77 1 5.48 1.04
E
rRNA modification in the nucleus and cytosol
Major pathways of rRNA processing in the nucleolus and cytosol
Eukaryotic Translation Termination
Response of EIF2AK4(GCN2) to amino acid defeciency
SRP-dependent cotranslational protein targeting to membrane
Keratinization
B-WICH complex positivily regulates rRNA expression
[-log10 p value]
F
Cytoplasm
Nucleus
FRAx597
[µM, 18 h]
kDa
pY-STAT5
STAT5
pY-STAT3
STAT3
α-TUBULIN
HISTONE H3
1 0.35 0.36 1 0.27 0.16
1 0.49 1.03 1 0.28 0.43
1 0.28 0.49 1 0.33 0.33
1 0.42 0.80 1 0.35 1.16

Figure EV3.

	JPX-0750	IQDMA	FRAx597
H1	6.78	16.16	3.10
H2	13.26	7.99	<100
H3	6.27	1.73	<100
H4	11.57	1.58	<100
H5	0.94	0.68	3.50
H6	3.51	2.52	<100
H7	9.50	8.02	1.59
H8	9.23	8.75	n.d.
H9	0.80	0.86	0.95
H10	1.54	0.97	1.64
H11	1.05	0.88	0.78
H12	1.22	0.71	1.41
H13	1.65	0.86	1.37
H14	0.95	1.00	0.86
H15	1.28	1.10	1.52

IC_{50} [μM, 48 h]

Figure EV4. JPX-0750, IQDMA, and FRAx597 show varying toxicity to healthy cells.

Heatmap showing IC_{50} values upon treatment of primary PBMCs isolated from healthy controls with JPX-0750, IQDMA, and FRAx597. One experiment performed in triplicates using CellTiter-Glo viability assays upon 48 h drug treatment is shown. The relative STAT3/5 log_2 ratio value is depicted in gray.

Source data are available online for this figure.

Figure EV5. The PAK kinase inhibitor inhibits malignant cell dissemination into the lymph nodes *in vivo*.

A–C Flow cytometric analysis of malignant cell dissemination into (A) lymph nodes, (B) liver, and (C) kidney (Vehicle: $n = 3$, JPX-0750: $n = 3$, IQDMA: $n = 3$; Vehicle: $n = 2$, FRAx597: $n = 3$, with n representing the number of analyzed tumors per group) as measured by the percentage of human $CD45^{+}$ cells in the respective organ. Error bars represent mean ± SEM. Statistical significance was calculated using a two-tailed paired t-test with Welch's correction. P-value: < 0.05 (*).

D–F H&E and IHC analyses of Hut78-derived tumors treated with JPX-0750 ($n = 3$), IQDMA ($n = 2$) or vehicle ($n = 4$), and FRAx597 ($n = 3$) or vehicle ($n = 2$), and stained with (D) H&E and (E) cleaved caspase 3 (CC3) to detect cell death and (F) CD31 vessel marker, with n representing the number of analyzed tumors per group. Pictures shown are from contiguous sections. Dotted rectangles indicate magnified areas. Scale bars, 200, 100, and 25 μm. Violin plots show the perimeter of the annotated tumor cell infiltration/expansion region into the intradermal and subcutaneous region of the skin, as well as quantification of the percentage of $CC3^{+}$ cells in the tissue. CD31 staining was quantified as raw counts of vessels with lumen. Statistical significance was calculated using a two-tailed paired t-test with Welch's correction. P-value: < 0.05 (*), 0.0001 (****). The bold dashed line in the middle of the violin plot denotes the median value, while the thin dotted lines denote the interquartile range.

Source data are available online for this figure.

© 2022 The Authors

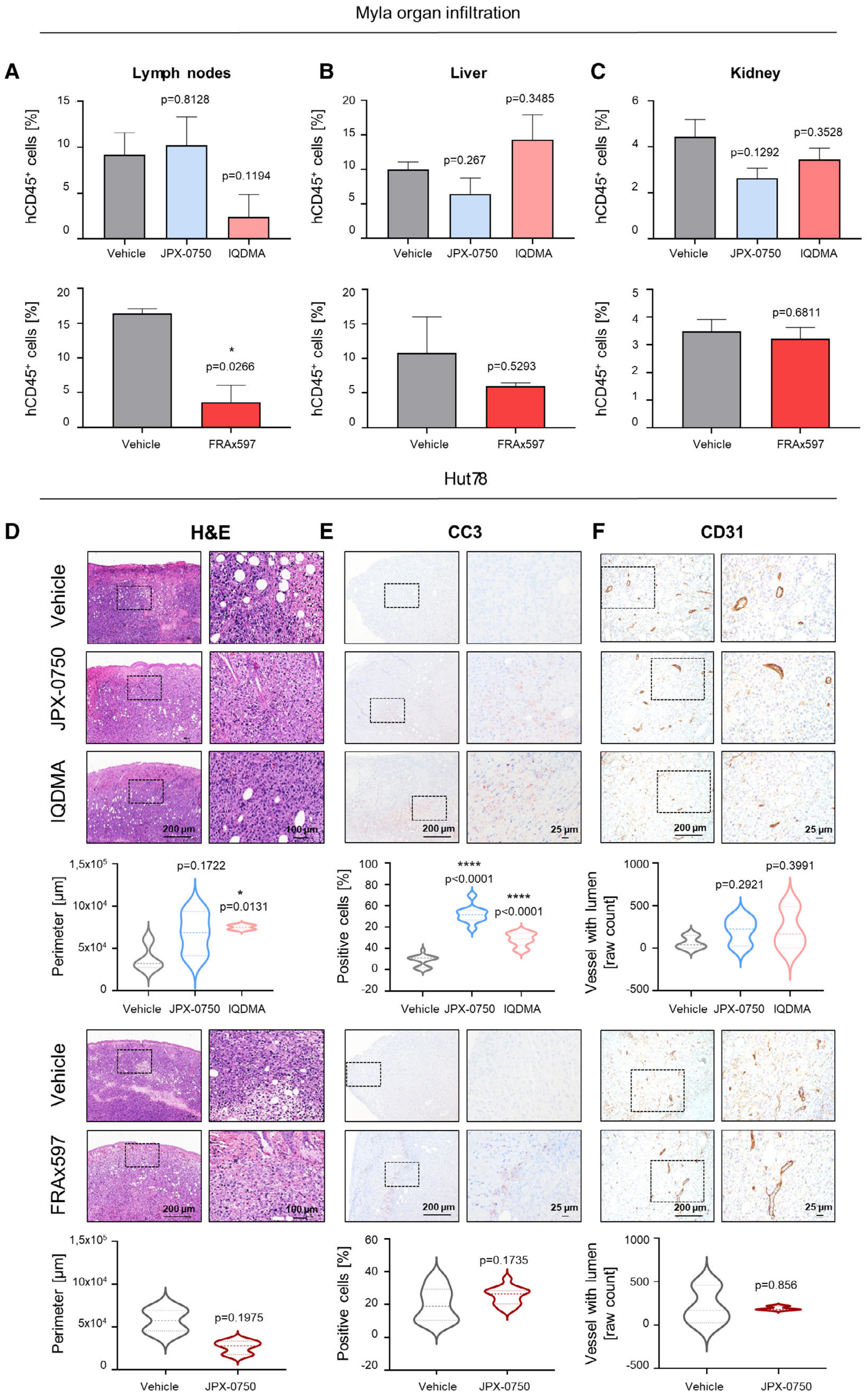

Myla organ infiltration
A
Lymph nodes
B
Liver
C
Kidney
hCD45+ cells [%]
Vehicle
JPX-0750
IQDMA
FRAx597
p=0.8128
p=0.1194
p=0.267
p=0.3485
p=0.1292
p=0.3528
p=0.0266
p=0.5293
p=0.6811
Hut78
D
H&E
E
CC3
F
CD31
200 µm
100 µm
25 µm
Perimeter [µm]
Positive cells [%]
Vessel with lumen [raw count]
p=0.1722
p=0.0131
p<0.0001
p=0.2921
p=0.3991
p=0.1975
p=0.1735
p=0.856

Figure EV5.
