# Supplementary figures and images for: Blocking STAT3/5 through direct or upstream kinase targeting in leukemic cutaneous T‐cell lymphoma
Source: EMBO Mol Med. 2022 Nov 7;14(12):e15200. doi: 10.15252/emmm.202115200 (PMC9727928; doi:10.15252/emmm.202115200)

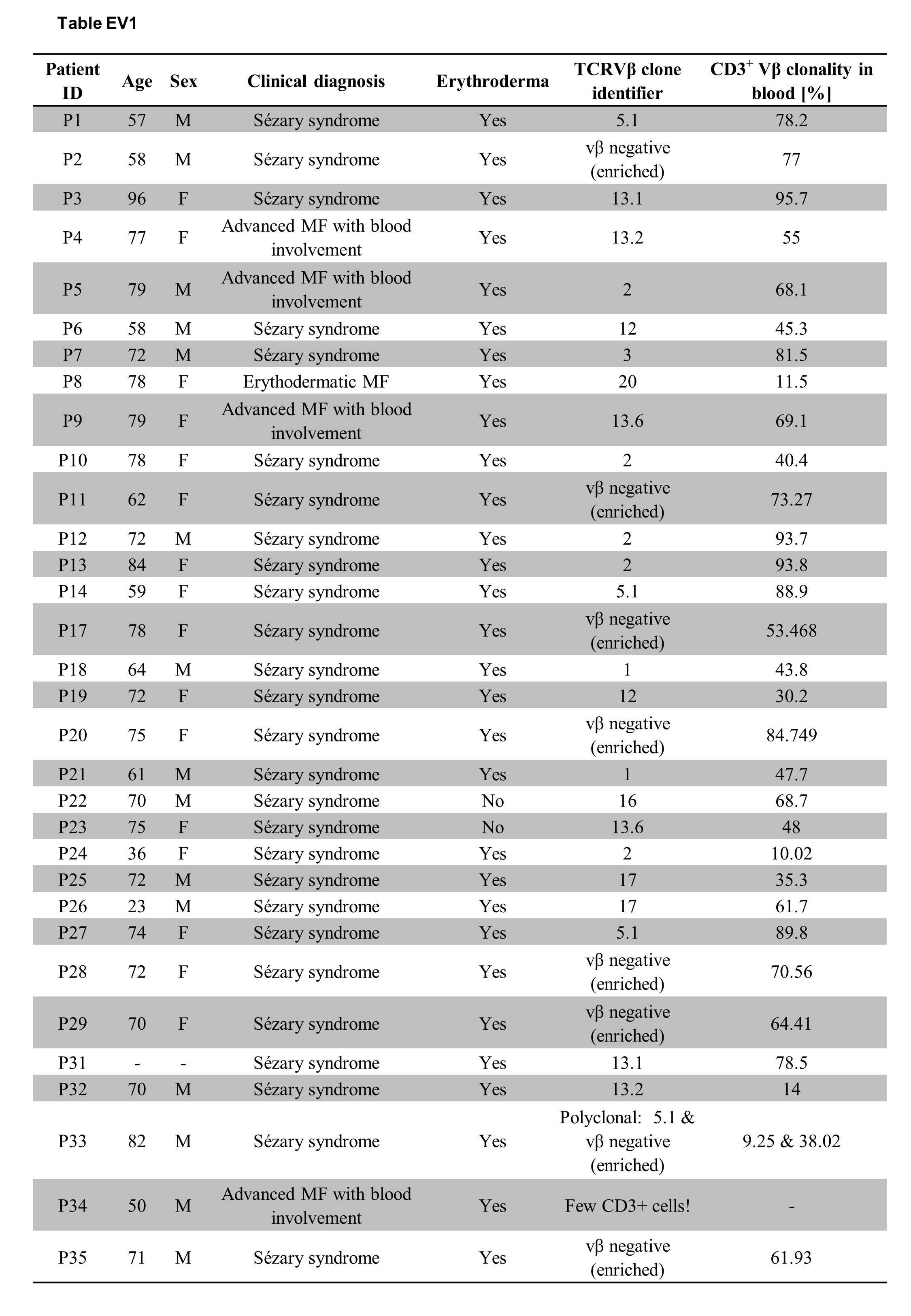

Supplement: Supplementary file 3 — Table EV1 [file EMMM-14-e15200-s017.tif]

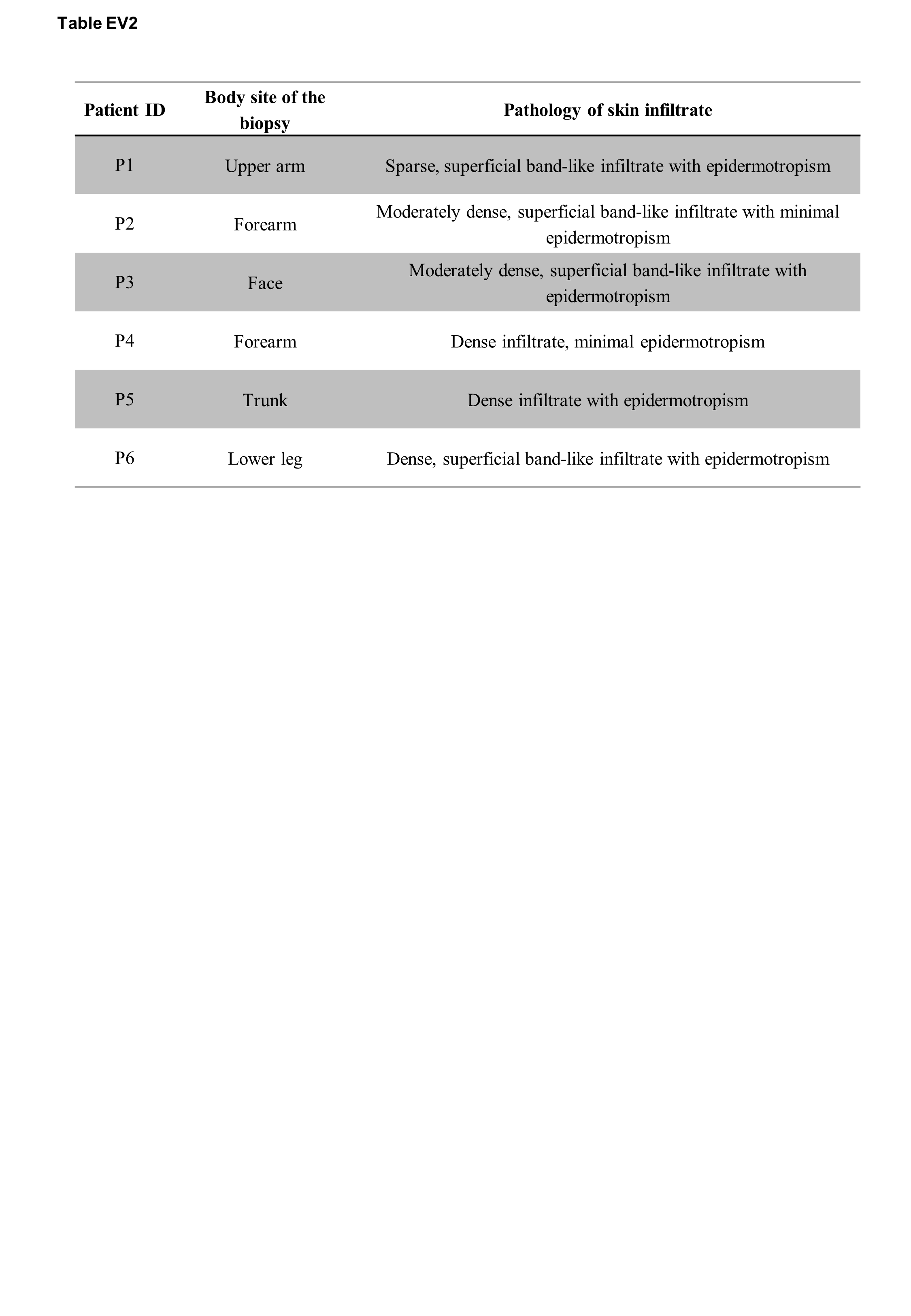

Supplement: Supplementary file 4 — Table EV2 [file EMMM-14-e15200-s007.tif]

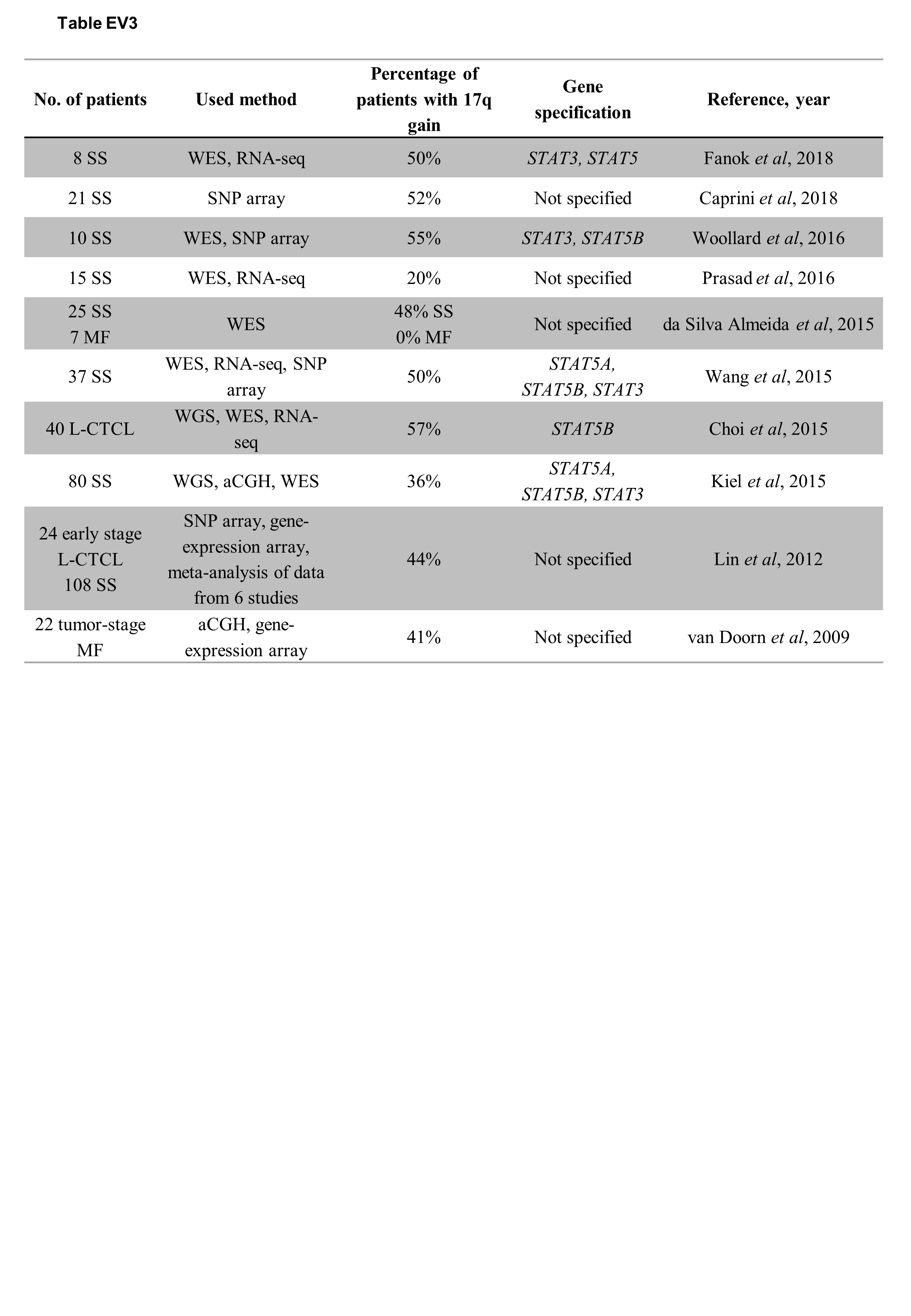

Supplement: Supplementary file 5 — Table EV3 [file EMMM-14-e15200-s023.tif]

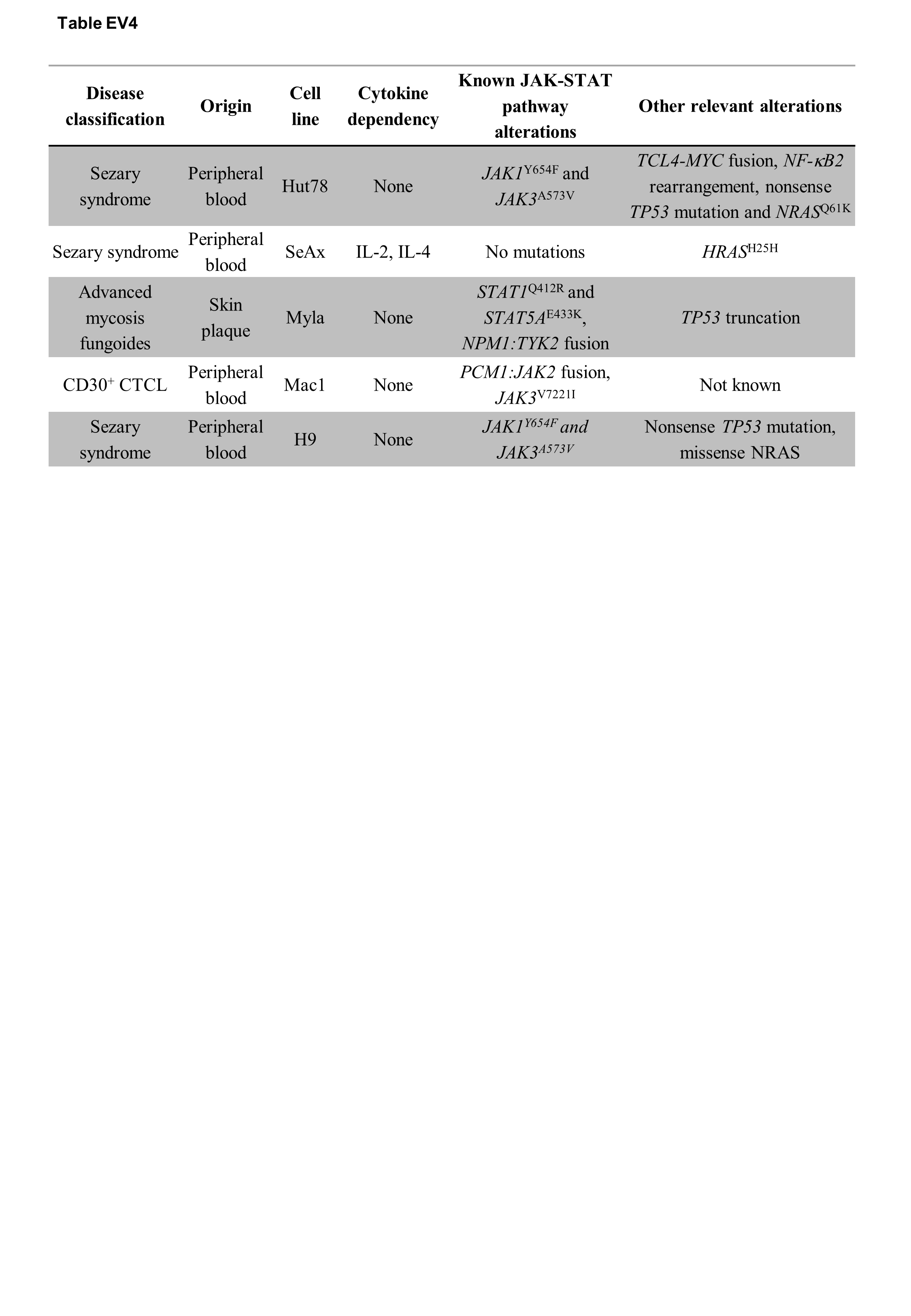

Supplement: Supplementary file 6 — Table EV4 [file EMMM-14-e15200-s018.tif]

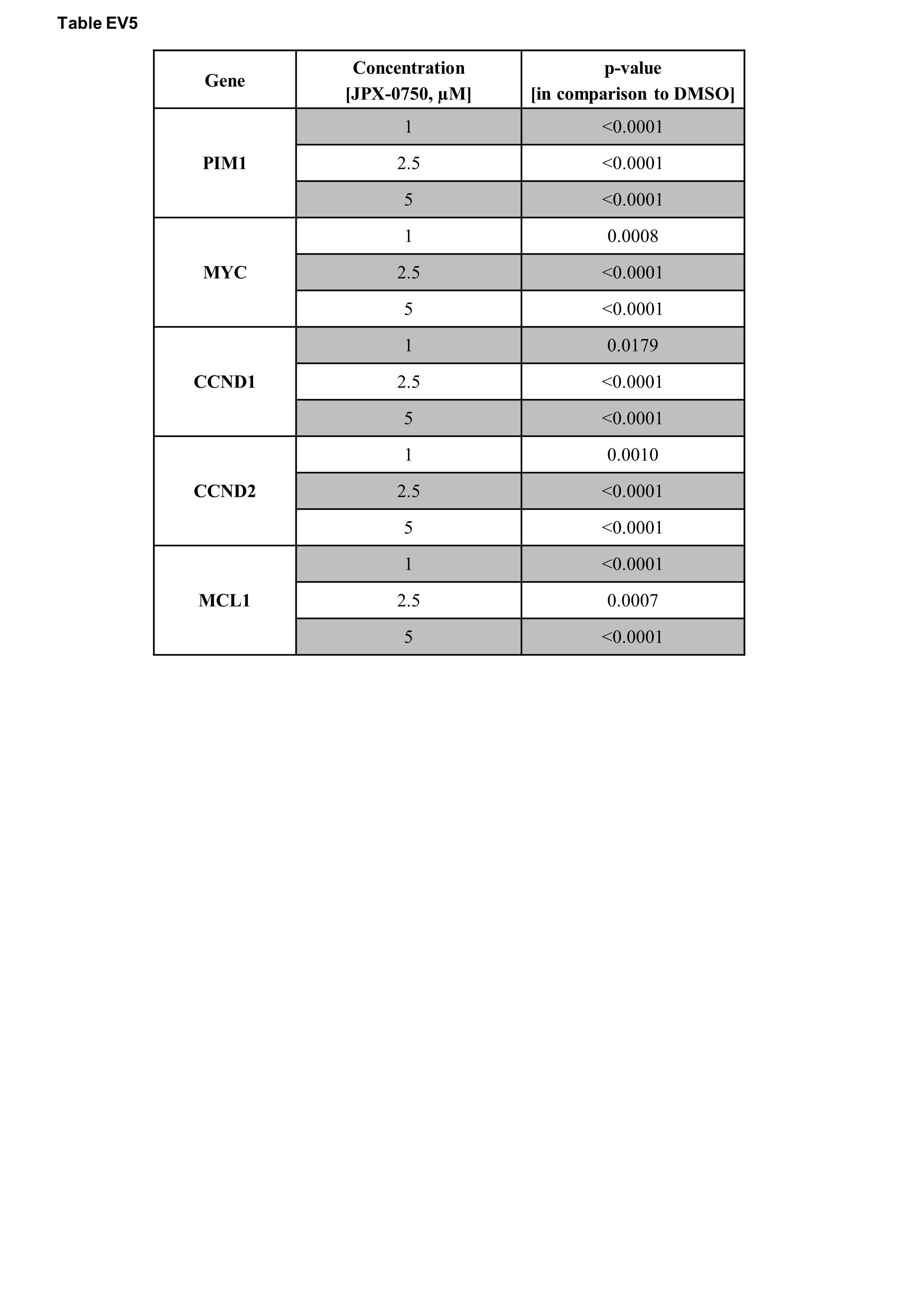

Supplement: Supplementary file 7 — Table EV5 [file EMMM-14-e15200-s013.tif]

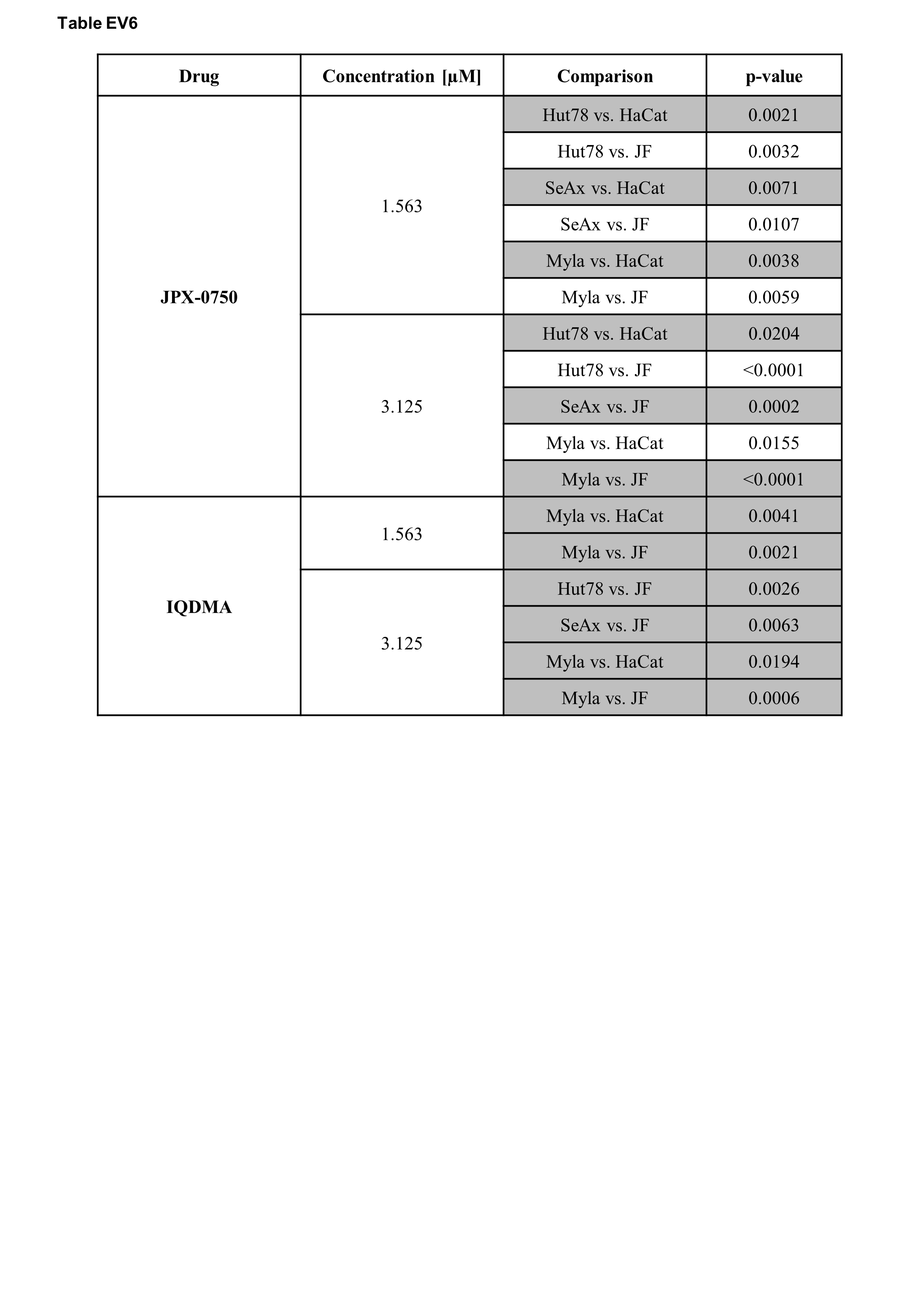

Supplement: Supplementary file 8 — Table EV6 [file EMMM-14-e15200-s015.tif]
