# Supplementary material for: Blocking STAT3/5 through direct or upstream kinase targeting in leukemic cutaneous T‐cell lymphoma
Source: EMBO Mol Med. 2022 Nov 7;14(12):e15200. doi: 10.15252/emmm.202115200 (PMC9727928; doi:10.15252/emmm.202115200)
Supplement: Supplementary file 23 [file EMMM-14-e15200-s021.pdf]

EMBO Molecular Medicine

Blocking STAT3/5 through direct or upstream kinase targeting in leukemic cutaneous T-cell lymphoma

Helena Sorger, Saptaswa Dey, Pablo Vieyra-Garcia, Daniel Pölöske, Andrea Teufelberger, Elvin Araujo, Abootaleb Sedighi, Ricarda Graf, Benjamin Spiegl, Isaac Lazzeri, Till Braun, Ines Garces de los Fayos Alonso, Michaela Schlederer, Gerald Timelthaler, Petra Kodajova, Christine Pirker, Marta Surbek, Michael Machtinger, Thomas Graier, Isabella Perchthaler, Yi Pan, Regina Fink-Puches, Lorenzo Cerroni, Jennifer Ober, Moritz Otte, Jana Albrecht, Gary Tin, Ayah Abdeldayem, Pimyupa Manaswiyoungkul, Olasunkanmi Olaoye, Martin Metzelder, Anna Orlova, Walter Berger, Marion Wobser, Jan Nicolay, Fiona Andre, Van Nguyen, Heidi Neubauer, Roman Fleck, Olaf Merkel, Marco Herling, Ellen Heitzer, Patrick Gunning, Lukas Kenner, Richard Moriggl, and Peter Wolf
DOI: 10.15252/emmm.202115200

Corresponding authors: Richard Moriggl (Richard.Moriggl@vetmeduni.ac.at) , Patrick Gunning (patrick.gunning@utoronto.ca), Richard Moriggl (Richard.Moriggl@vetmeduni.ac.at), Lukas Kenner (lukas.kenner@meduniwien.ac.at), Peter Wolf (peter.wolf@medunigraz.at)

Review Timeline:

Submission Date:	28th Sep 21
Editorial Decision:	27th Oct 21
Revision Received:	1st Aug 22
Editorial Decision:	12th Sep 22
Revision Received:	26th Sep 22
Accepted:	2nd Oct 22

Editor: Lise Roth

Transaction Report:

(Note: With the exception of the correction of typographical or spelling errors that could be a source of ambiguity, letters and reports are not edited. Depending on transfer agreements, referee reports obtained elsewhere may or may not be included in this compilation. Referee reports are anonymous unless the Referee chooses to sign their reports.)

27th Oct 2021

Dear Prof. Moriggl,

Thank you for the submission of your manuscript to EMBO Molecular Medicine, and please accept my apologies for the delay in getting back to you, which is due to the fact that we were waiting for the report from referee #2.
In order not to delay the process further, and after additional consultation with the referees, we prefer to make a decision without the evaluation from referee #2 at this point. Should this referee provide a report shortly, we will send it to you, with the understanding that we would not ask you for extensive experiments in addition to the ones required in the enclosed reports.

As you will see below, the reviewers raise substantial concerns on your work, which unfortunately preclude its publication in EMBO Molecular Medicine in its current form.
Indeed, the reviewers find that the question addressed by the study is of potential interest, however they remain unconvinced that some of the major conclusions are sufficiently supported by the data. They raise the following major issues:

- overstatements in the conclusions
- lack of rigor in some experiments (Western blots quantification, controls, statistics)
- limited translational advance (small sample size regarding the patients' cells and no in vivo validation).

If you feel you can satisfactorily address these points and those listed by the referees, you may wish to submit a revised version of your manuscript. In particular, we would like you to adequately qualify the study (no overstatements and mention of the caveats), strengthen the conclusiveness of the experiments (controls, statistics, etc), and improve the translational advance by increasing the patients' cells sample size and/or performing in vivo experiments.
Please attach a covering letter giving details of the way in which you have handled each of the points raised by the referees. A revised manuscript will once again be subject to review, and we cannot guarantee at this stage that the eventual outcome will be favorable.
EMBO Molecular Medicine encourages a single round of revision only and therefore, acceptance or rejection of the manuscript will depend on the completeness of your responses included in the next, final version of the manuscript. For this reason, and to save you from any frustrations in the end, I would strongly advise against returning an incomplete revision.

When submitting your revised manuscript, please carefully review the instructions that follow below. We perform an initial quality control of all revised manuscripts before re-review; failure to include requested items will delay the evaluation of your revision.

We require:

1) A .docx formatted version of the manuscript text (including legends for main figures, EV figures and tables). Please make sure that the changes are highlighted to be clearly visible.

2) Individual production quality figure files as .eps, .tif, .jpg (one file per figure). For guidance, download the 'Figure Guide PDF' (https://www.embopress.org/page/journal/17574684/authorguide#figureformat).

3) A .docx formatted letter INCLUDING the reviewers' reports and your detailed point-by-point responses to their comments. As part of the EMBO Press transparent editorial process, the point-by-point response is part of the Review Process File (RPF), which will be published alongside your paper.

4) A complete author checklist, which you can download from our author guidelines (https://www.embopress.org/page/journal/17574684/authorguide#submissionofrevisions). Please insert information in the checklist that is also reflected in the manuscript. The completed author checklist will also be part of the RPF.

5) Please note that all corresponding authors are required to supply an ORCID ID for their name upon submission of a revised manuscript. We note that you currently have together with you, a total of 4 co-corresponding authors. Is that correct? Do you confirm equal contribution of these 4 people, able to take full responsibility for the paper and its content? While there is no limit per se to the number of co-corresponding authors, 3 is rare, 4 even more so, and may not reflect as intended to the community.

6) It is mandatory to include a 'Data Availability' section after the Materials and Methods. Before submitting your revision, primary datasets produced in this study need to be deposited in an appropriate public database, and the accession numbers and database listed under 'Data Availability'. Please remember to provide a reviewer password if the datasets are not yet public (see https://www.embopress.org/page/journal/17574684/authorguide#dataavailability).

In case you have no data that requires deposition in a public database, please state so in this section. Note that the Data Availability Section is restricted to new primary data that are part of this study.

7) For data quantification: please specify the name of the statistical test used to generate error bars and P values, the number (n) of independent experiments (specify technical or biological replicates) underlying each data point and the test used to calculate p-values in each figure legend. The figure legends should contain a basic description of n, P and the test applied. Graphs must include a description of the bars and the error bars (s.d., s.e.m.).

8) We would also encourage you to include the source data for figure panels that show essential data. Numerical data should be provided as individual .xls or .csv files (including a tab describing the data). For blots or microscopy, uncropped images should be submitted (using a zip archive if multiple images need to be supplied for one panel). Additional information on source data and instruction on how to label the files are available at
.

9) Our journal encourages inclusion of *data citations in the reference list* to directly cite datasets that were re-used and obtained from public databases. Data citations in the article text are distinct from normal bibliographical citations and should directly link to the database records from which the data can be accessed. In the main text, data citations are formatted as follows: "Data ref: Smith et al, 2001" or "Data ref: NCBI Sequence Read Archive PRJNA342805, 2017". In the Reference list, data citations must be labeled with "[DATASET]". A data reference must provide the database name, accession number/identifiers and a resolvable link to the landing page from which the data can be accessed at the end of the reference. Further instructions are available at .

10) We replaced Supplementary Information with Expanded View (EV) Figures and Tables that are collapsible/expandable online. A maximum of 5 EV Figures can be typeset. EV Figures should be cited as 'Figure EV1, Figure EV2" etc... in the text and their respective legends should be included in the main text after the legends of regular figures.
- For the figures that you do NOT wish to display as Expanded View figures, they should be bundled together with their legends in a single PDF file called *Appendix*, which should start with a short Table of Content. Appendix figures should be referred to in the main text as: "Appendix Figure S1, Appendix Figure S2" etc.
- Additional Tables/Datasets should be labeled and referred to as Table EV1, Dataset EV1, etc. Legends have to be provided in a separate tab in case of .xls files. Alternatively, the legend can be supplied as a separate text file (README) and zipped together with the Table/Dataset file.
See detailed instructions here:
.

11) The paper explained: EMBO Molecular Medicine articles are accompanied by a summary of the articles to emphasize the major findings in the paper and their medical implications for the non-specialist reader. Please provide a draft summary of your article highlighting
- the medical issue you are addressing,
- the results obtained and
- their clinical impact.
This may be edited to ensure that readers understand the significance and context of the research. Please refer to any of our published articles for an example.

12) For more information: There is space at the end of each article to list relevant web links for further consultation by our readers. Could you identify some relevant ones and provide such information as well? Some examples are patient associations, relevant databases, OMIM/proteins/genes links, author's websites, etc...

13) Every published paper now includes a 'Synopsis' to further enhance discoverability. Synopses are displayed on the journal webpage and are freely accessible to all readers. They include a short stand first (maximum of 300 characters, including space) as well as 2-5 one-sentences bullet points that summarizes the paper. Please write the bullet points to summarize the key NEW findings. They should be designed to be complementary to the abstract - i.e. not repeat the same text. We encourage inclusion of key acronyms and quantitative information (maximum of 30 words / bullet point). Please use the passive voice. Please attach these in a separate file or send them by email, we will incorporate them accordingly.

Please also suggest a striking image or visual abstract to illustrate your article as a PNG file 550 px wide x 300-600 px high.

14) As part of the EMBO Publications transparent editorial process initiative (see our Editorial at http://embomolmed.embopress.org/content/2/9/329), EMBO Molecular Medicine will publish online a Review Process File (RPF) to accompany accepted manuscripts.
In the event of acceptance, this file will be published in conjunction with your paper and will include the anonymous referee reports, your point-by-point response and all pertinent correspondence relating to the manuscript. Let us know whether you agree with the publication of the RPF and as here, if you want to remove or not any figures from it prior to publication.
Please note that the Authors checklist will be published at the end of the RPF.

EMBO Molecular Medicine has a "scooping protection" policy, whereby similar findings that are published by others during review or revision are not a criterion for rejection. Should you decide to submit a revised version, I do ask that you get in touch after three months if you have not completed it, to update us on the status.

I look forward to seeing a revised form of your manuscript as soon as possible. Use this link to login to the manuscript system and submit your revision: Link Not Available

With kind regards,

Lise Roth

Lise Roth, Ph.D
Scientific Editor
EMBO Molecular Medicine

***** Reviewer's comments *****

Referee #1 (Comments on Novelty/Model System for Author):

The observation of 17q gains and hyper-activation of STAT3/5 in CTCL being an important oncogenic driver of this disease is already well accepted and is supported by many publications. Indeed some of the current approved therapeutic strategies, eg HDACi were developed based on STAT3/5 hyper-activation, and so targeting these proteins is not a very novel concept for this disease. On the other hand there is definitely a need for new more effective targeting strategies to be developed. The model system used in this study are human CTCL cell lines. While the use of cell lines is perfectly acceptable for establishing effects of the drugs on specific tumor cells in the first instance, further experiments need to be performed to progress the in vitro data and show some translational capability of these drugs as therapeutic strategy in CTCL patients. Ideally, an animal model of this disease or NSG mice engrafted with leukemic cells from patients would be needed to understand the therapeutic usefulness of these drugs in CTCL and maybe other leukemias. Some attempt to evaluate the effects of the drugs on CTCL patient PBMC is made but only 3 of 6 patients are used in the analyses (the choice of selective patients is not made clear)and many more patient samples need to be evaluated to provide rigorous experimental significance to the findings.

Referee #1 (Remarks for Author):

This study explores the potential usefulness of the drug IQDMA in therapeutic targeting of STAT3/5 in CTCL patients with 17q gains. IQDMA has been reported as STAT5 inhibitor in the literature, but the authors would like to demonstrate that this is a multi-kinase inhibitor based on data provided in the study. Further, they propose that it's mode of action on CTCL cell lines is being mediated via direct inhibition of PAK1, leading to subsequent STAT3/5 inhibition by blocking nuclear translocation of these STAt proteins. The weakness of the study is that it is fully based on in-vitro analysis of the drugs on CTCL cell lines and a few CTCL patient PBMC, which does not provide any information about their usefulness for therapeutic translation. The data presented in this study do not fully support their claims that IQDMA inhibits STAT3/5 by inhibition of PAK1, and more robust experimentation is required before the study can be considered for publication in EMBO Molecular Medicine.

Specific Points

1. My main concerns are the patient sample sizes used in the study, which is very small and the lack of in-vivo data, given the premise of the study is to evaluate the usefulness of a drug for inhibiting CTCL growth and survival. Given the multiple genetic aberrations identified in CTCL patients, sampling just six patients to study the effects of specific drugs in-vitro does not provide robust data to make generalized conclusions about its potential efficacy as a targeting strategy for leukemic CTCL. There are six patients included in this study and yet the biochemical and viability assays are only performed on patients 4 and 5 primarily and P6 in one experiment. Why were the others not analyzed and on what criteria were only these two patients chosen for the majority of the experiments with patient samples? As the demographics of this disease is that it affects older individuals, were the healthy control group age matched also? Please provide some more details.

2. The western blot depicted in Figure 4B should be quantified and statistics performed so that subtle changes in STAT3/5 protein (especially with IQDMA treatment) can be clearly evaluated. This experiment should also be performed on all the cell lines use din the viability and apoptosis assays, because for instance Hut78 is later used in the viability assay, but is not characterized for its STAT3/5 dose response to IQDMA and JPX0750.

3.The viability and apoptosis experiments shown in Figure 4cd use different concentrations of drugs; apoptosis/necrosis at 2.5uM and viability 0.75-3.12 uM, with significant effects on viability observed at 1.56 uM and we are not shown a "no" treatment control. Why use different doses in the different experiments on the same cell lines? It's very confusing without explanation for

why different doses are used, and also why a dose below 2.5uM (for JPX-0750) or 5uM (for IQDMA), which doesn't seem to have any effect of STAT3/5 proteins, has such a significant effect on cell viability and apoptosis?

4.In Figure 5C, again a different dose of IQDMA is used in the kinome analysis. This time the experiment has been done with 10uM IQDMA, at which dose viability and apoptosis seems to be maximally inhibited and from the western blot (fig 4b)the control protein HSC70 also seems to be affected, suggesting that the drug is
acting by non-specifically inducing apoptosis at this dose. Therefore, the kinome screen should really be done at a much lower dose (<5uM) to be consistent with the earlier analyses and at where it might be functioning in a more STAT-specific manner. Does IQDMA target all these kinases (and specifically PAK1) at lower doses (<2.5uM), or only a subset?

5. The western blot in figure 5E should be repeated multiple times and quantified or use another technique such as Imagestream to get quantitative data, as the data shown here is not convincing in terms of an effect on disruption of STAT nuclear translocation, which only seems to happen at 10uM, whereas all the earlier data seem to show an effect on viability/apoptosis at lower does <2.5uM. As such, together the data in Fig 5E and F would suggest that IQDMA has a generalised inhibitory effect on CTCL viability and apoptosis and is not mediated via inhibition of STAT5/3 (even indirectly).

6. The experiments in Figure 5E and F should be performed with the PAK1 inhibitor to show its effect on STAT3/5, and to prove that IQDMA is working through PAK1, ideally PAK1 should be knocked out/down and then treated with IQDMA to support the claim that the inhibition of STAT3/5 by IQDMA is mediated through PAK1.

7. Figure 6A shows very varied effects on some STAT3/5 regulated target genes, with some being inhibited with increasing doses, while some not at all, and others in between these two scenarios.
Again, these blots should be quantified by densitometry to provide significance to the results.

Minor point:

Figure legend to Fig 3b, please describe what HH is.

Referee #3 (Comments on Novelty/Model System for Author):

L-CTCL patients with 17q copy number alterations show enhanced STAT3/5 expression which in turn correlates with disease severity. The authors hypothesize that STAT3/5 targeting could be beneficial in arresting L-CTCL development. They provide experimental support for this hypothesis using human cell models, and the analyses performed by the authors in this regard are adequate. The authors then continue to validate the action of IQDMA as a multi-kinase inhibitor and CTCL cell death inducer, followed by an exploration of the apoptosis mechanism.

PAK2 was determined to be one of the kinases whose activity was blocked by IQDMA. This was followed by the observation that the PAK2 inhibitor drug was the only one with activity specific to the STAT3/5 gain, as PAK2 regulates nuclear shuttling of STAT5. They conclude that L-CTCL cells can be targeted by blocking the STAT3/5 pathway directly or indirectly, through dual STAT inhibitors or PAK2 inhibitors respectively, to similar extents.

Referee #3 (Remarks for Author):

The manuscript is clear in analysis and content, but here are some minor comments to help improve the quality further.

1) More details need to be included in the Methods section to increase clarity for the readers. Certain areas include a) TCR-beta repertoire -- expand on technique, method, and identification of clones, b) CNA identification -- expand on ichorCNA and parameters using for identifying CNAs, p/q-values of alterations, etc. c) Synergy screen -- explain the purpose of ZIP model and cNMF algorithm, what 4 parameters in logistic regression, etc.. d) Expression analysis -- describe the Caprini lymphoma dataset, exactly what was extracted from Oncomine platform and the original paper, what was the Spearman correlation used for, and so on.

2) Include test identity, metrics, p-values in the main results section whenever a statistical comparison/evaluation is made. This improves clarity for the reader and provides context to how conclusions were drawn in the text.

Part 2

As indicated by the Scientific Editor Dr. Lise Roth, the following major points were raised:

● overstatements in the conclusions

● lack of rigor in some experiments (Western blots quantification, controls, statistics)

● limited translational advance (small sample size regarding patient cells and no *in vivo* validation).

We thank the Editor for pointing out these weaknesses, which we tried to eliminate in the revised version of this manuscript. We amended and marked text changes as indicated by yellow highlighting to remove overstatements in our conclusions at multiple points and along reviewer concerns, as specifically indicated below in the point-to-point reply. Moreover, we expanded experimentally on new insights in main **Figure 1, 3, 5, 6** and **Figure 7**, accompanied by additions in Expanded View/Supplementary Figures, also detailed below. Furthermore, we improved the scientific rigor of experiments (particularly Western blot analysis).

Most importantly, even though it is a very rare disease, by reaching out to international collaborators, we acquired 29 additional L-CTCL patient samples, out of which 17 had a good enough quality for sequencing. Moreover, we were able to perform drug testing on 15 primary patient-derived PBMCs, as well as evaluate toxicity on healthy PBMCs (n=15), collectively enabling us to draw better conclusions. As requested by the reviewers/editor, we also performed xenografting of three different CTCL patient-derived cell lines that display *STAT3/5* gains with enhanced STAT3/5 expression, where we included the three selected targeted drugs for *in vivo* effect evaluation. For these experiments, an animal license was filed to fulfill ethical requirements and new results are shown in **Figure 7 / Figure EV5 / Appendix Figure S6**. In addition, we conducted a more detailed whole-genome sequencing analysis, shedding light on tumor suppressors frequently genetically lost in several L-CTCL patient samples and negatively regulating STAT3/5 signaling, namely *STAT1* and *SOCS1* (shown in new **Figure 1**). These findings were also confirmed by aCGH genetic analysis of the now five CTCL cell lines used in the study (one more established patient-derived CTCL cell line was included also in light of conducting *in vivo* testing with drugs). The aCGH analysis is now included in the updated **Figure 3**. We are aware that this was not requested by reviewers, but our consortium agreed that it could complement the genetic analysis, where negative cytokine/growth factor signaling through SOCS1 ubiquitinylation

processes or oncogenic STAT3/5 family member antagonisation via STAT1 could counteract STAT3/5 hyperactivation. Thus, we believe that these chromosomal deletions in distinct CTCL model cell systems with similar genetic hallmarks could identify possible negative regulators of STAT3/5. We feel that we have sufficiently addressed all reviewer points and extended our analysis to gain sufficient molecular insight and novelty, where we also addressed editor concerns. We hope that we now provide a complete revision with more substantial insights into the role of *STAT3/5* gain for L-CTCL cancer cell biology. We should now better meet the standards and requirements for EMBO Molecular Medicine to achieve acceptance of the revised version, needed also for the graduation work of the two first authors that performed most of the experiments in joint efforts.

Part 3

Referee #1 (Comments on Novelty/Model System for Author):
The observation of 17q gains and hyper-activation of STAT3/5 in CTCL being an important oncogenic driver of this disease is already well accepted and is supported by many publications. Indeed some of the current approved therapeutic strategies, eg HDACi were developed based on STAT3/5 hyper-activation, and so targeting these proteins is not a very novel concept for this disease. On the other hand, there is definitely a need for new more effective targeting strategies to be developed. The model system used in this study are human CTCL cell lines. While the use of cell lines is perfectly acceptable for establishing effects of the drugs on specific tumor cells in the first instance, further experiments need to be performed to progress the *in vitro* data and show some translational capability of these drugs as therapeutic strategy in CTCL patients. Ideally, an animal model of this disease or NSG mice engrafted with leukemic cells from patients would be needed to understand the therapeutic usefulness of these drugs in CTCL and maybe other leukemias. Some attempt to evaluate the effects of the drugs on CTCL patient PBMC is made, but only 3 of 6 patients are used in the analyses (the choice of selective patients is not made clear) and many more patient samples need to be evaluated to provide rigorous experimental significance to the findings.

We thank the reviewer for recognizing merit in our study and according to the reviewer suggestion, we tried to reinforce experimental significance of the findings by firstly performing extensive xenograft experiments with three CTCL cell lines presenting with *STAT3/5* gains, namely Hut78, Myla and the newly added H9 (which is thought to be a subclone of Hut78, but we detected

genetic differences via aCGH analysis). Interestingly, the tumor growth rates in mice were different among the chosen models, possibly as a consequence of their different genetic make-up or microenvironment interplay, blood vessel formation, apoptotic, as well as proliferative indexes, that we all monitored in the consecutive sections using established biomarkers for histopathology evaluation (**Figure 7, Figure EV5**).

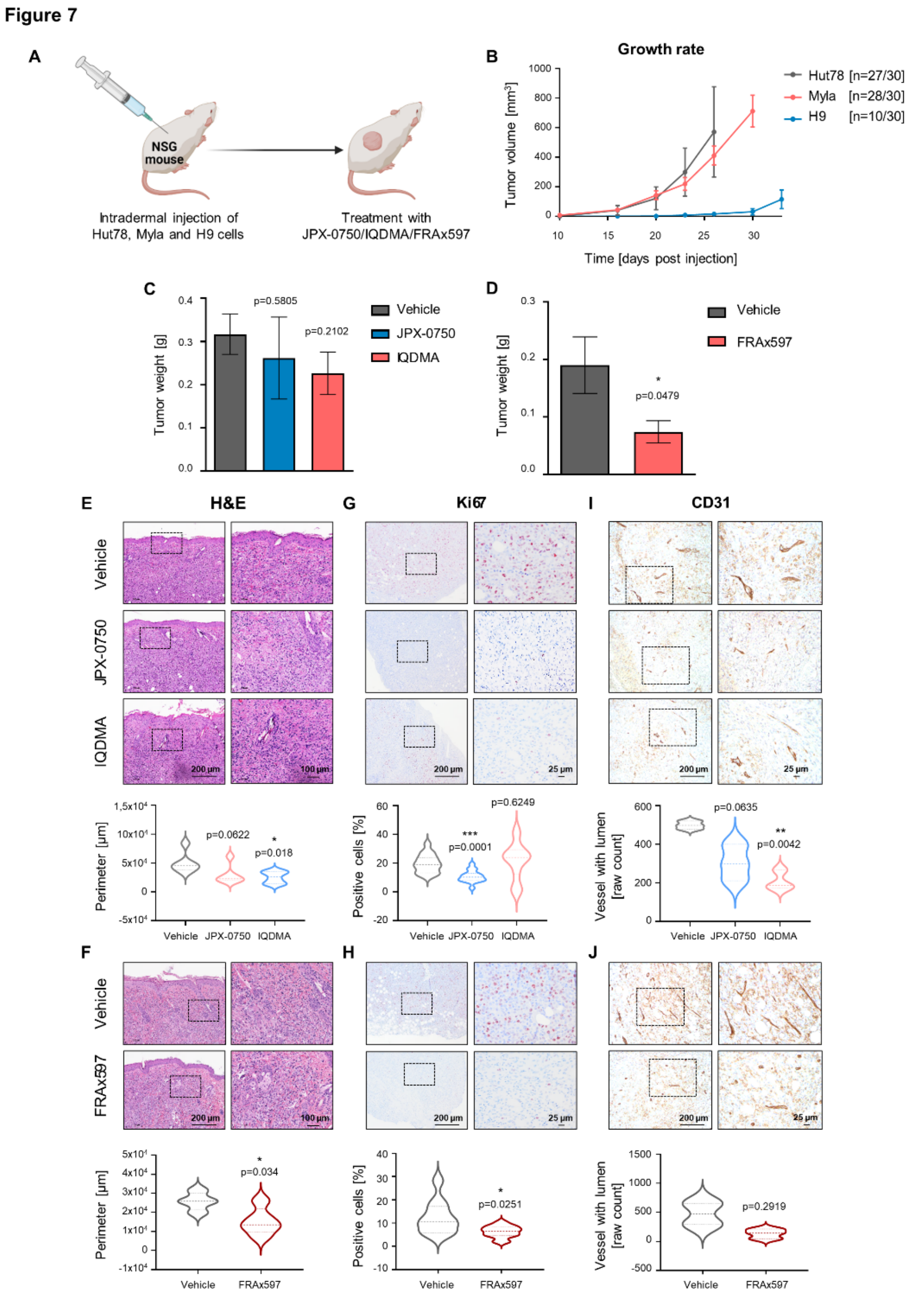

Figure 7 in the revised manuscript

Figure EV5

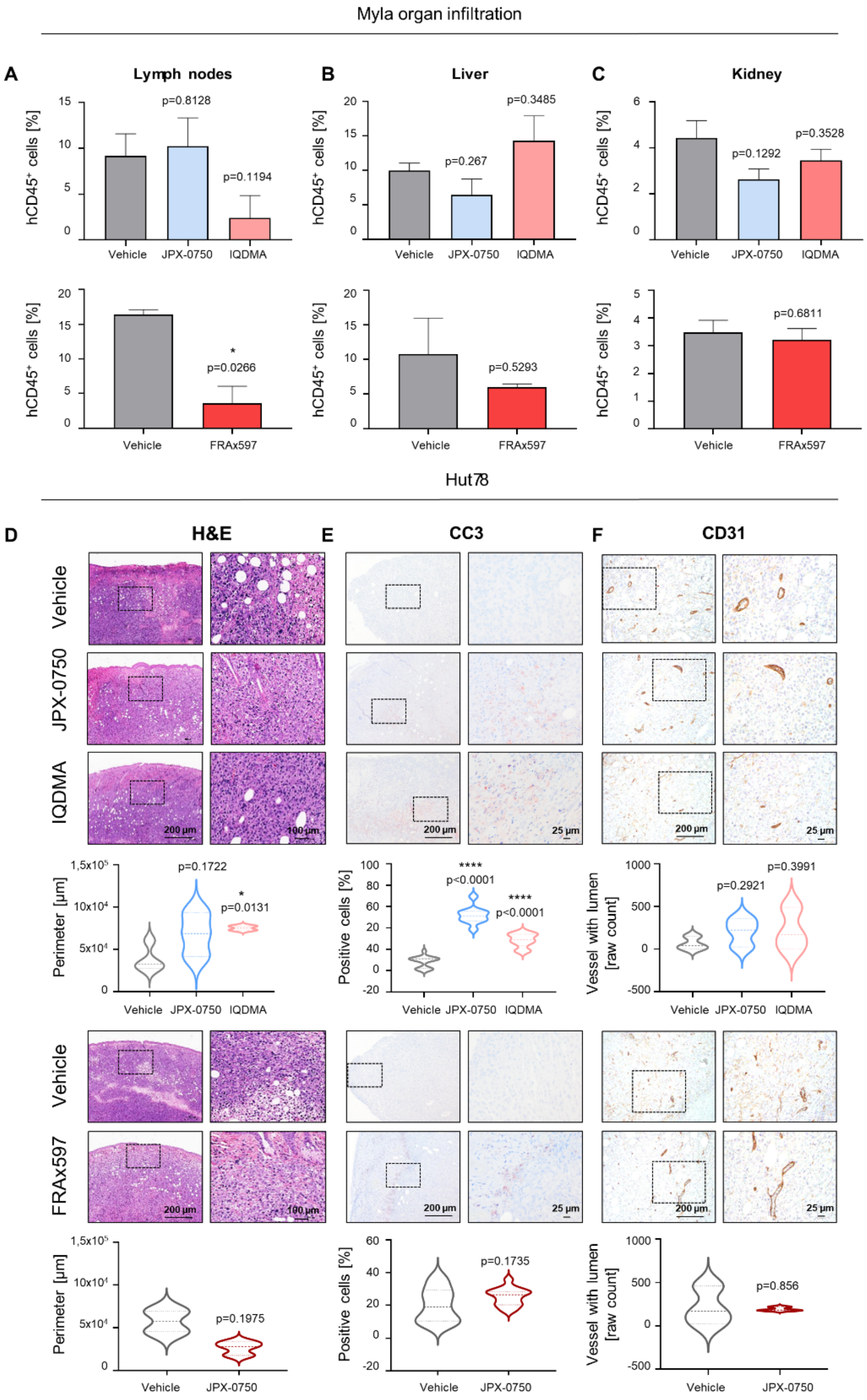

Figure EV5 in the revised manuscript

The most successful cell lines with 90% and 93% engraftment success were Hut78 and Myla, respectively. With Myla, we conducted a 20-day treatment regime using the three different targeted drugs identified *in vitro* as most promising in the original submission. We evaluated potential toxicity, as well as the impact of these drugs on L-CTCL disease progression in NSG mice. Corresponding to the aggressive nature of the disease in human patients, the intradermally injected malignant T-cells disseminated into the lymph nodes, liver and kidney and we could detect via flow cytometry human $CD45^+$ blast cells in these organs. Histopathology analysis of the respective xenograft tumors was carefully performed by two board-certified and haematology-trained cancer specialized pathologists. The PAK inhibitor, FRAx597, had the most significant impact on reducing tumor growth, where we would like to also conclude that lead structures such as JPX-0750 or IQDMA need to be developed further with medicinal chemistry lead structure development efforts to achieve better pharmacodynamics/pharmacokinetics for *in vivo* applications.

The Hut78 cell line showed an exponential growth rate, and the treatment had to be terminated after 10 days due to disease progression with skin ulceration of the tumor area. Still we conducted thorough histopathological analysis and included the data into **Figure EV5** (shown on page 9).

The H9 cell line, even though a subclone of Hut78, did not present a high enough engraftment success rate (only ~40%) to continue with treatment. However, it has significant genetic differences to Hut78, like chromosome 8, 15, or 18 differences affecting gene dosage, as highlighted in the aCGH analysis in **Appendix Figure S4**, suggesting a different clonal outgrowth.

Appendix Figure S4

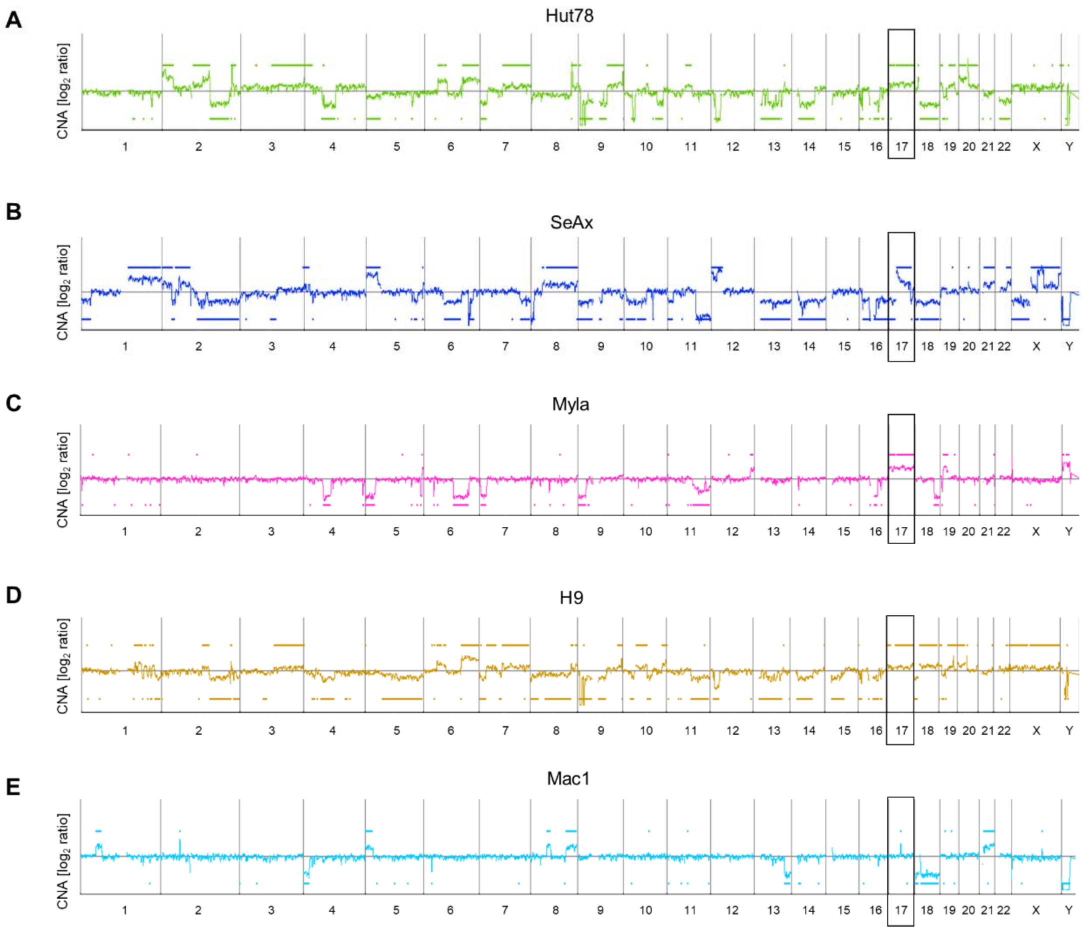

Appendix Figure S4 in the revised manuscript

Secondly, we reached out to several international collaborators to increase the sample size of the study cohort. We might emphasize that CTCL is a very rare malignancy affecting ~1/100 000 individuals, out of which 10% will progress into the highly aggressive leukemic L-CTCL phenotype, displaying peripheral organ and blood malignant cell dissemination. Our efforts resulted in acquiring 29 additional L-CTCL patient samples, out of which 17 passed the quality control check necessary for whole genome sequencing, resulting in a total of 23 patients (17 + 6 included in the original submission). From the sequenced patients, 74% presented with a *STAT3/5* copy number gain (**Figure 1**).

Figure 1

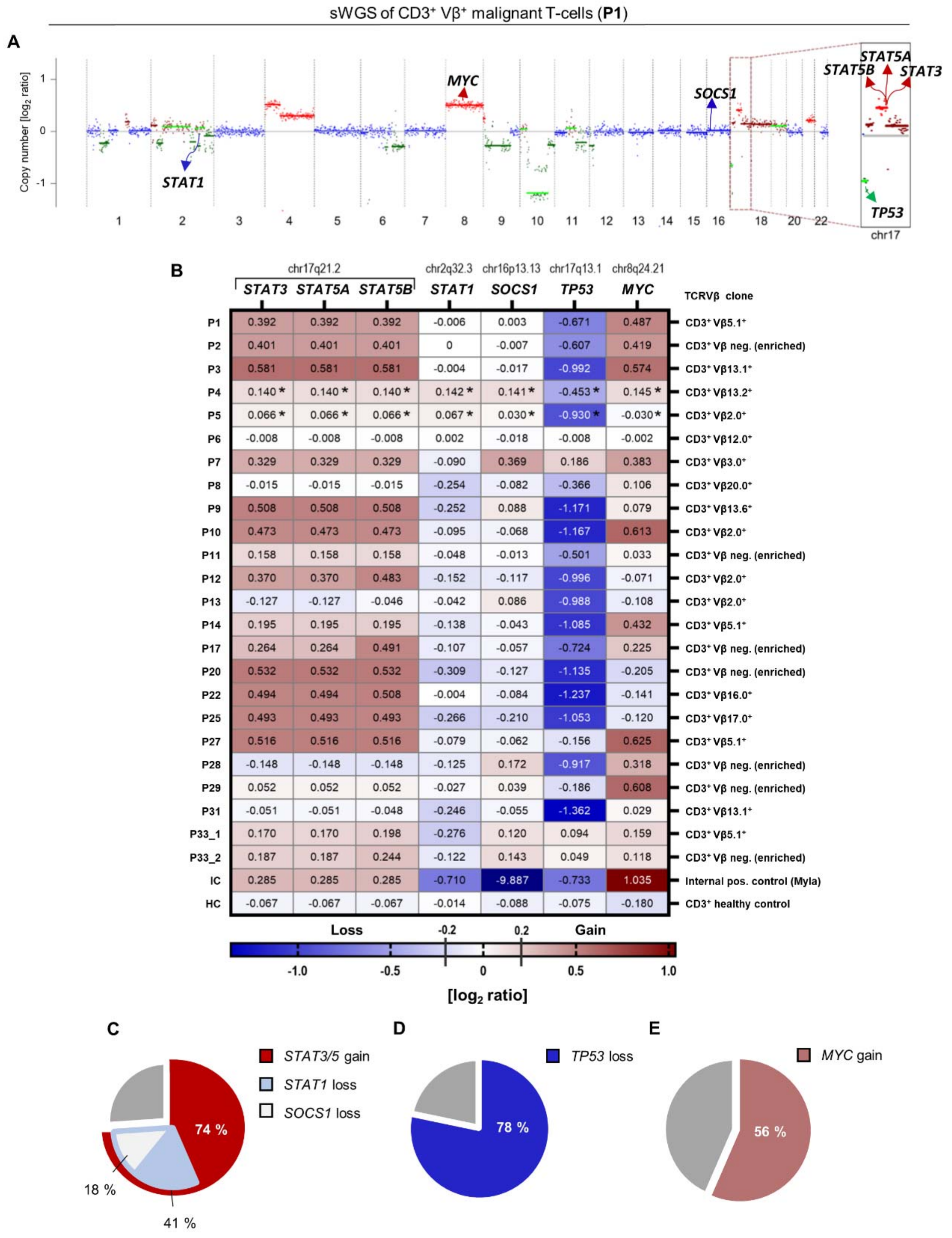

Figure 1 in the revised manuscript

Furthermore, the detected *STAT3/5* somatic copy number alteration (CNA) log_2 ratio positively correlated with the percentage of malignant T-cell clones detected in the blood of the

respective patients, highlighting the importance of *STAT3/5* gain for disease pathogenesis (**Figure 2A-C**). This finding re-confirmed our original hypothesis that targeting STAT3/5 will have merit for L-CTCL patients.

Figure 2

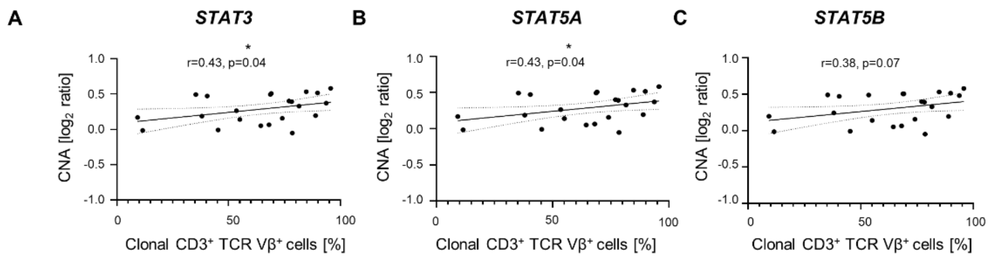

Figure 2, panel A-C in the revised manuscript

In addition, by conducting a more detailed whole-genome sequencing analysis, we also identified loss of tumor suppressors *STAT1* and *SOCS1* co-occurring with *STAT3/5* gains in patients and cell lines (**Figure 1** and **Figure 3**). We believe that loss of *STAT1* and *SOCS1* as negative regulators of STAT3/5 signaling, contributes to STAT3/5 hyperactivation, but further work is needed to investigate this.

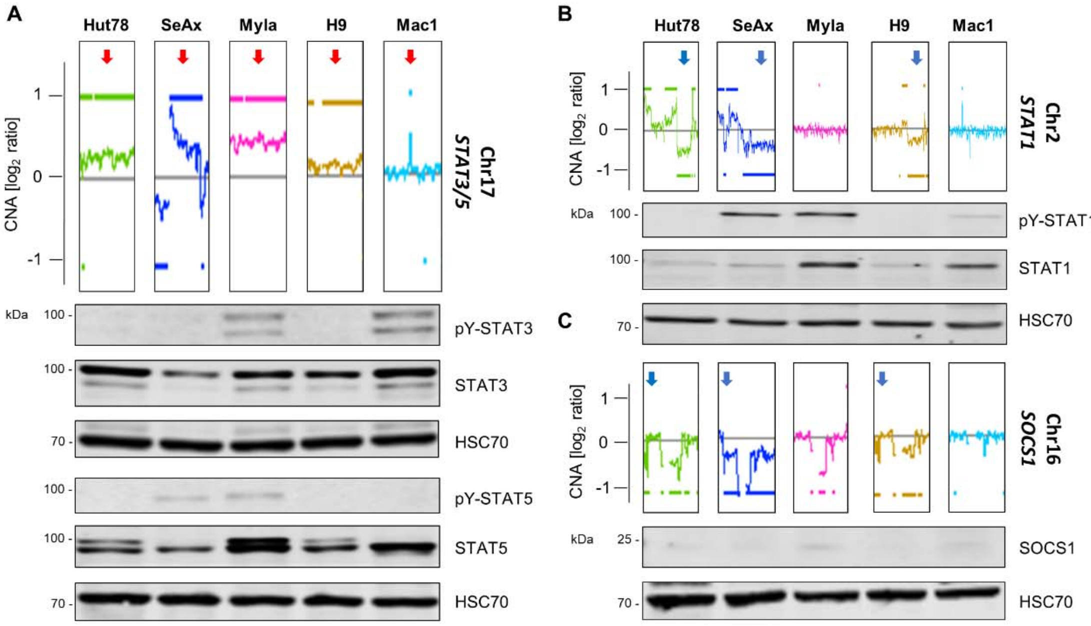

Figure 3 in the revised manucript

Finally, we performed drug testing with all three drugs with 15 out of 23 samples, which contained enough viable PBMCs to perform the experiment. Our drug screen revealed that indeed

the three selected drugs are efficient in killing the patient cells and revealed that the PAK inhibitor has better efficiency in cells with *STAT3/5* copy number gains (**Figure 6E**). Namely, the response inversely correlated with the respective CNA log_2 ratios (**Figure 6G-H**). The exact mechanism of action remains to be further elucidated, even though our data suggest that it suppresses STAT3/5 nuclear levels, similar to IQDMA. Nevertheless, lead structure development is required to obtain better inhibitors arresting aggressive L-CTCL malignant cell growth.

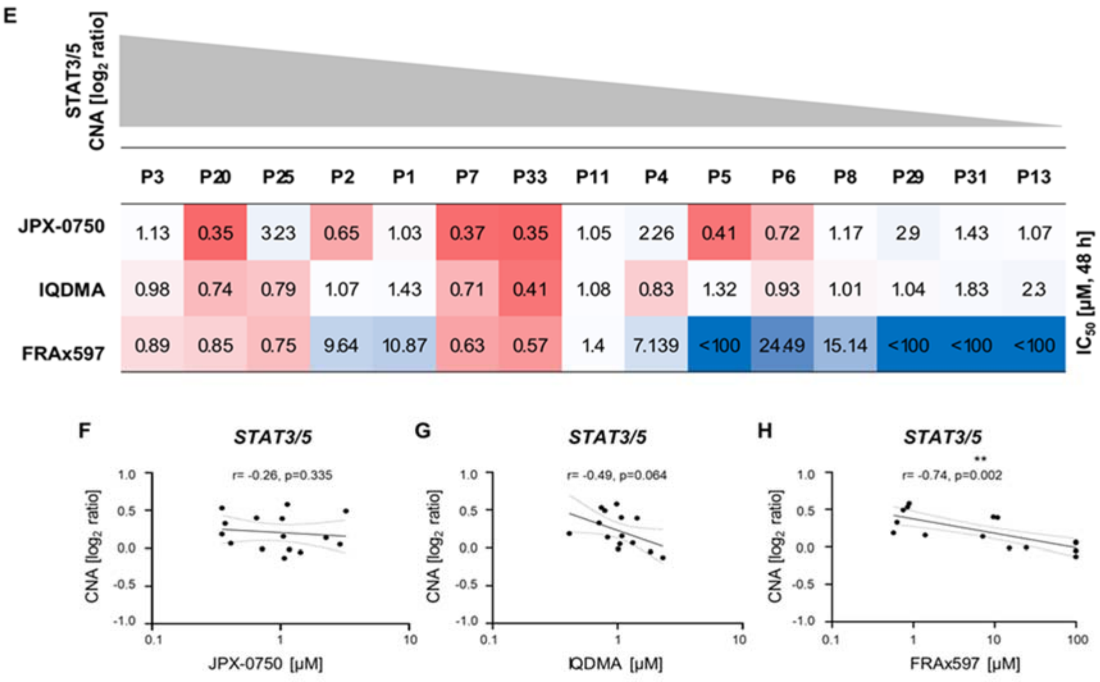

Figure 6E-F in the revised manuscript

Referee #1 (Remarks for Author):

This study explores the potential usefulness of the drug IQDMA in therapeutic targeting of STAT3/5 in CTCL patients with 17q gains. IQDMA has been reported as STAT5 inhibitor in the literature, but the authors would like to demonstrate that this is a multi-kinase inhibitor based on data provided in the study. Further, they propose that it's mode of action on CTCL cell lines is being mediated via direct inhibition of PAK1, leading to subsequent STAT3/5 inhibition by blocking nuclear translocation of these STAT proteins. The weakness of the study is that it is fully based on in-vitro analysis of the drugs on CTCL cell lines and a few CTCL patient PBMC, which does not provide any information about their usefulness for therapeutic translation. The data presented in this study do not fully support their claims that IQDMA inhibits STAT3/5 by inhibition of PAK1, and more robust

experimentation is required before the study can be considered for publication in EMBO Molecular Medicine.

We thank the reviewer for constructive criticism as well as attributing significance to the study, in particular in regards to IQDMA, where we tried to address the issues by re-stating our findings. We did originally propose that it is directly acting on the STAT3/5 axis through PAK2, but we agree with the reviewer that this is an overstatement and that we do not provide sufficient proof of this.

Investigation of the mechanism of action of IQDMA revealed that it is not a STAT5 inhibitor, as stated in the literature and on supplier websites:
https://www.abcam.com/iqdma-cell-permeable-stat5-inhibitor-ab141192.html,
https://www.sigmaaldrich.com/AT/en/product/mm/420294

By performing a kinome screen to broadly examine target engagement, we discovered that IQDMA is a multi-kinase inhibitor engaging with several tyrosine and serine/threonine kinases, one of which was also PAK2. Prompted by the already reported role of the PAK kinase on STAT5 signaling, we investigated the effect of IQDMA on STAT3/5 nuclear levels and observed that it downregulates activated and total STAT5 nuclear levels at 5µM and activated STAT3 levels at 10µM (**Figure 5B** and **Figure EV3D**) (1, 2). We therefore now describe IQDMA in the manuscript the following way:

Taken together, we conclude that IQDMA is a broad-range multi-kinase inhibitor that may indirectly block STAT3/5 nuclear translocation, but further investigation is required to explain the mechanism of action.

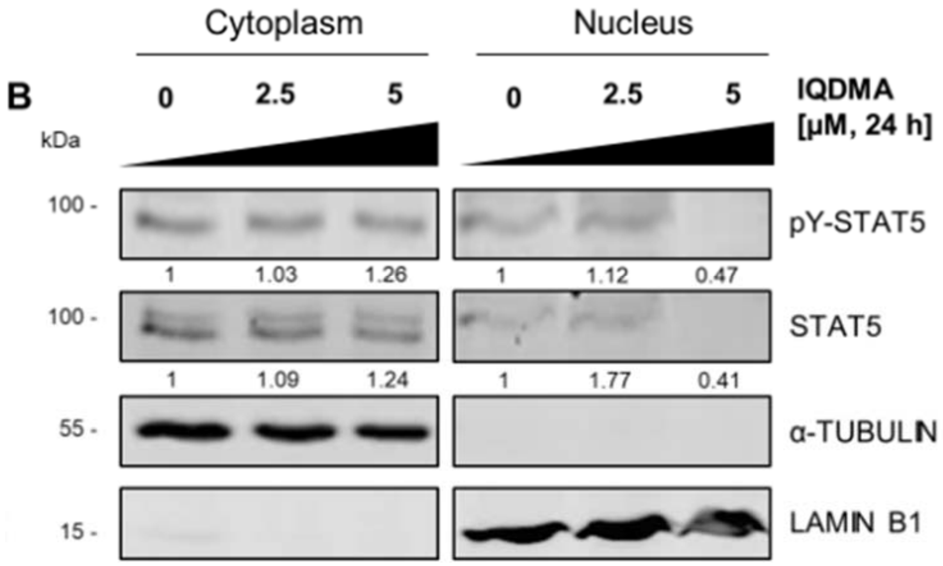

Figure 5B in the revised manuscript

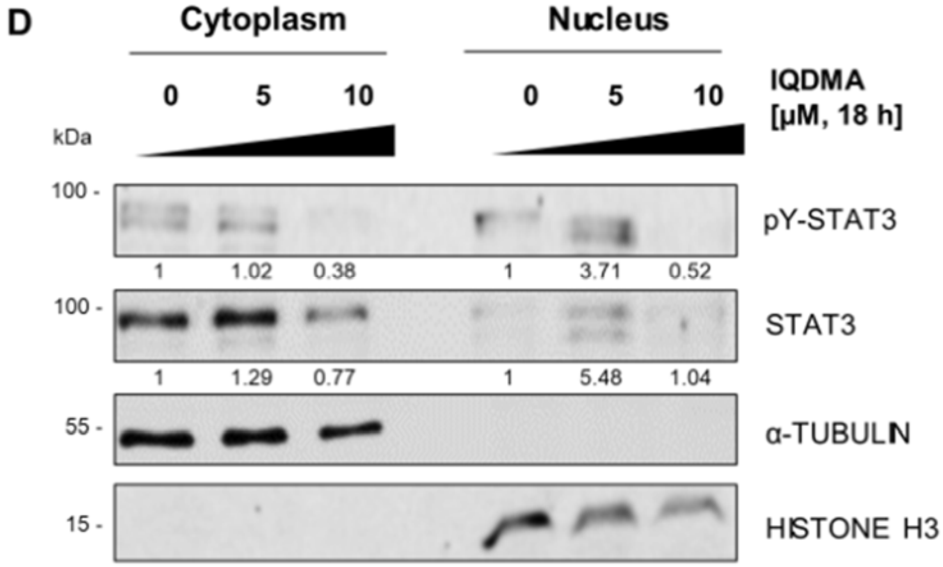

Figure EV3D in the revised manuscript

Furthermore, to examine IQDMA-specific effects in an unbiased and dose-dependent manner without limiting the analysis to only kinases, we carried out global proteomics profiling in SeAx cells after 24h treatment with 1, 2.5 and 10µM IQDMA. The differential proteins affected in response to increasing IQDMA concentrations were found to be related to multiple cellular processes in metabolism, ribosomal RNA processing/modification, protein translation, and surprisingly also keratinization (**Figure EV3E**). Insight into the most significant differentially

affected proteins at a low dose (1μM) revealed downregulation of key regulators of proliferation, which also regulate the immune response, such as K1C17, a type I intermediate filament chain Keratin 17 protein (**Figure 5D**). Importantly, the corresponding gene, *KRT17*, was previously shown to be upregulated in CTCL skin lesions. At higher doses (10μM), downregulation of a STAT3/5 downstream target involved in cell cycle progression, CCND2, was evident (**Figure 5F**).

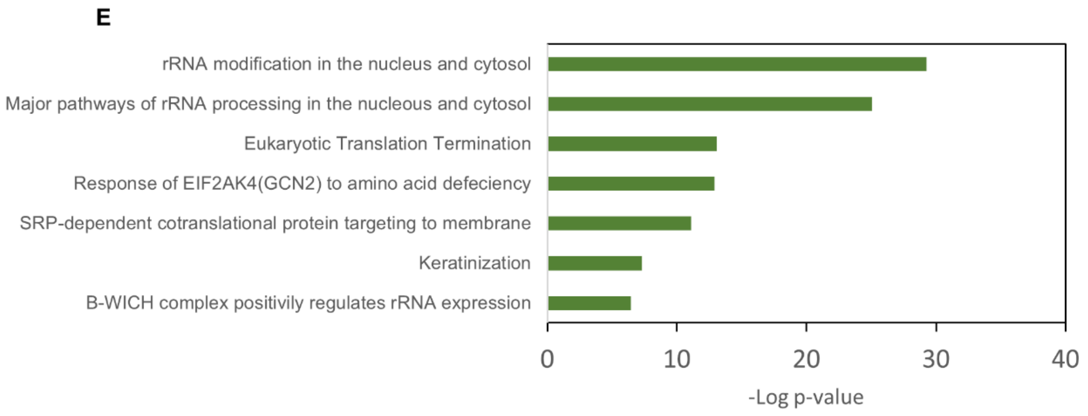

Figure EV3E in the revised manuscript

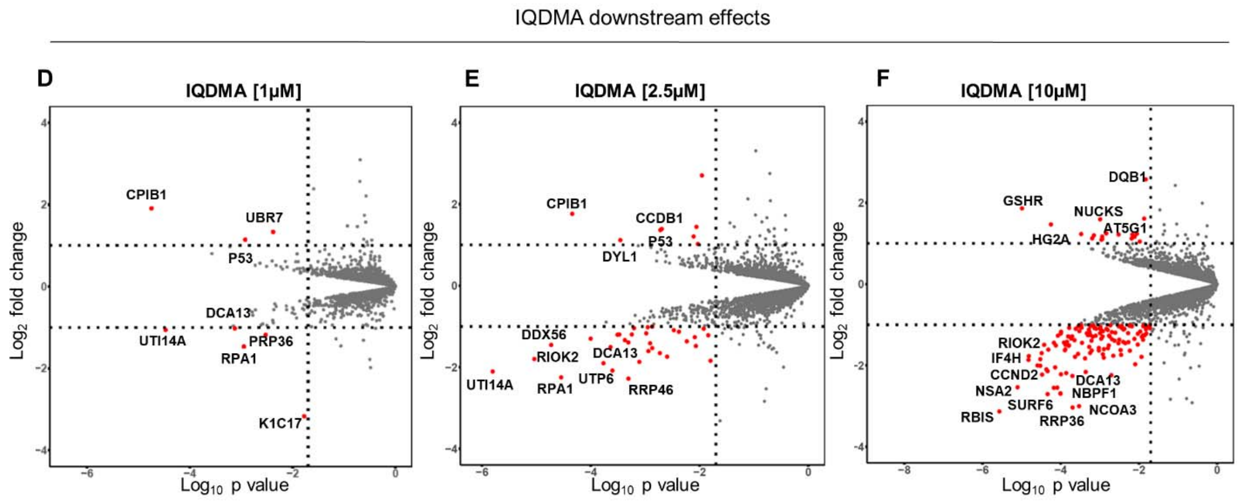

Figure 5D-F in the revised manuscript

The impact of IQDMA on key protein levels associated with hyperactive STAT3/5 action that we selected further for Western blot analysis illuminates the mode of action with downregulation of STAT3/5 downstream targets.

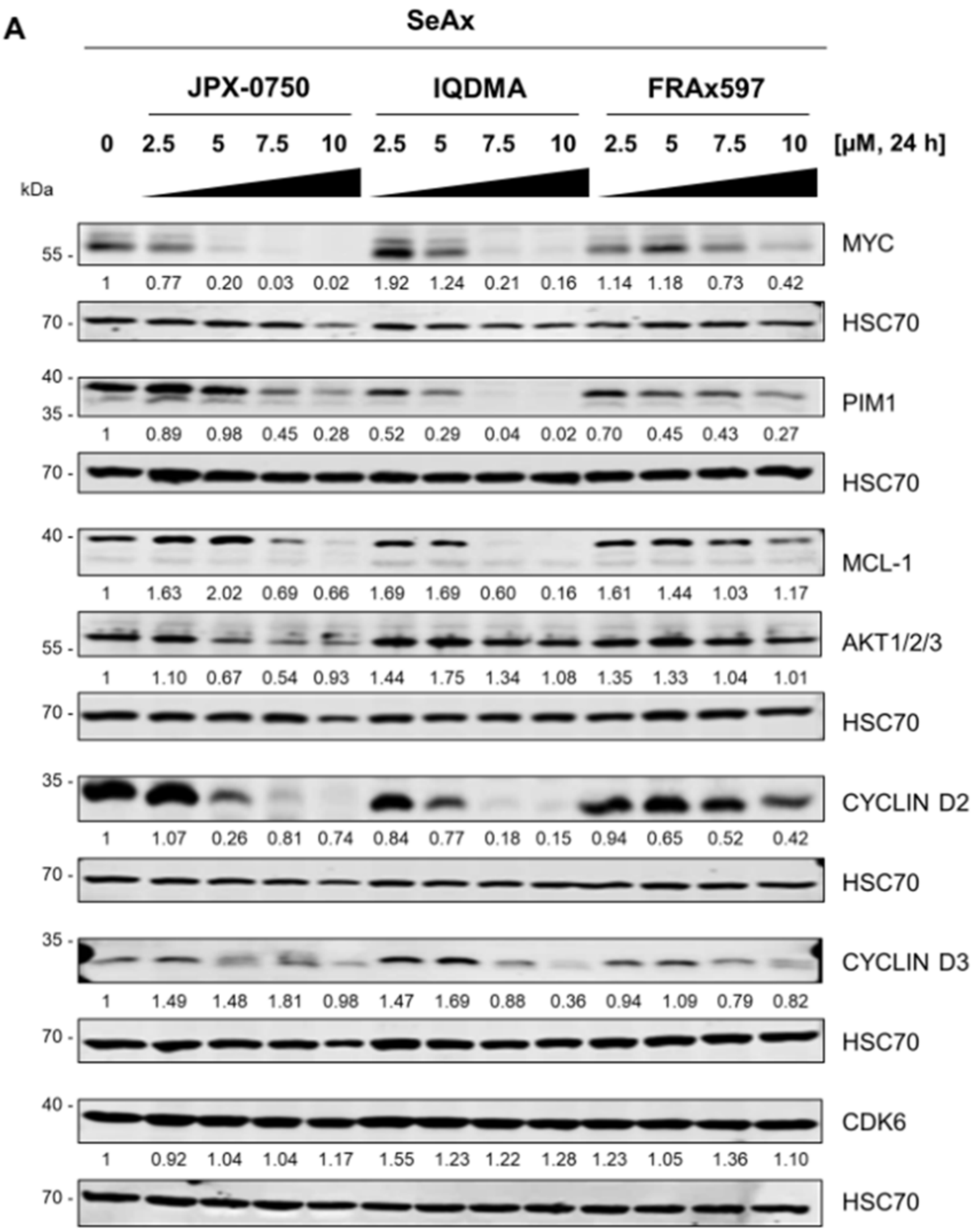

Figure 6A in the revised manuscript

Therefore, we conclude that IQDMA represents a broad-range kinase inhibitor that enabled us to select potential kinase drugs acting in CTCL cells with STAT3/5 gains, out of which the most promising one seemed to be the PAK inhibitor, even though the mechanism of action remains to

be further elucidated, potentially by genetic experiments knocking down or genetically deleting PAK kinase member(s).

Regarding the robustness, we have now included the analysis on 15 patient-derived and 15 healthy control-derived PBMCs. Our drug screen revealed that indeed the three selected drugs are efficient in killing the patient cells, where particularly the PAK inhibitor has better efficiency in CTCL cells with *STAT3/5* copy number gains (**Figure 6E-H**, shown on page 14). The exact mechanism of action remains to be further elucidated and lead structure optimization is required to obtain a clinical grade candidate to make these new drugs better suitable for patient clinical trial phase direction.

Moreover, we also included an additional CTCL cell line with *STAT3/5* gain, named H9, and included its drug response data, which was similar to the other three CTCL cell lines with *STAT3/5* gains that we had already analyzed for targeted drug efficacy as originally submitted (**Figure 4A and Figure 5G**).

Figure 4

A

IC$_{50}$ [μM, 48 h]

Target	Drug	Hut78	SeAx	Myla	H9	Mac1
USP1	Pimozide	11.16	6.55	9.09	5.92	5.57
STAT5	AC-4-130	12.84	8.67	13.83	3.21	5.58
STAT3	DR-155	5.29	2.08	3.49	2.91	3.81
STAT3/5	JPX-0700	1.74	1.61	1.98	1.29	0.68
STAT3/5	JPX-0750	1.18	1.34	1.26	0.41	1.02
Unknown	IQDMA	0.97	1.63	0.69	0.56	0.54

Figure 4A in the revised manuscript

G

STAT3/5 gain (Hut78, SeAx, Myla, H9, Mac1); IC$_{50}$ [μM, 48 h]

Target	Affinity [nmol/L]	Drug	Hut78	SeAx	Myla	H9	Mac1	HH
Abl/PDGFRα/VEGFR2/FGFR1/Src	0.4/1.1/1.5/2.2/5.4	Ponatinib	2.22	1.83	1.49	1.51	0.92	2.05
ALK/IGF-1R/InsR/STK22D/FLT3	0.2/8/7/23/60	Ceritinib	3.61	1.68	1.97	2.87	2.05	2.09
JAK1/2/3	112/20/1	Tofacitinib	> 200	> 200	> 200	> 200	1.08	> 200
JAK1/2/3/TYK2	1/1/5/1	JAK inhibitor I	> 200	> 200	> 200	> 200	0.35	> 200
Raf-1/B-Raf/VEGFR2/3/PDGFRβ/FLT3/Kit	6/22/90/20/57/59/68	Sorafenib	9.82	10.78	13.11	6.24	8.86	10.17
PAK1/2/3	8/13/19	FRAx597	1.97	1.27	1.77	1.50	0.89	62.21
PIM1/2/3	0.4/5/1.9	AZD1208	54.52	35.71	54.26	25.51	20.28	61.58

Figure 5G in the revised manuscript

Upon testing the H9 cell line with the kinase drugs, we observed an unexpected effect of Sorafenib on H9 (IC_{50} = 6.24µM). Originally, we claimed that Sorafenib did not have any effect on the CTCL cell lines (IC_{50} value was <100µM for Hut78, SeAx, Myla, Mac1 and HH cells), prompting us to repeat the analysis. However, we observed that this might have been due to an oxidized drug aliquot. Thus, we have carried out multiple repeats to ensure results on Sorafenib. We now included IC_{50} values from newly performed experiments and we changed the description of Sorafenib according to the new reliable results. We apologize for inconvenience in original negative description to Sorafenib, that we hope to cover now better for possible future studies. Irrespective, *in vivo* application with Sorafenib is not advisable since the IC_{50} is in all L-CTCL model cell lines above 6 µM.

Furthermore, we also show drug synergies in the SeAx and Myla cells concluding that the JPX-0750 and IQDMA could be used in combination, and particularly the PAK inhibitor synergized with them, as well as with ponatinib and ceritinib (**Figure 6B-D**).

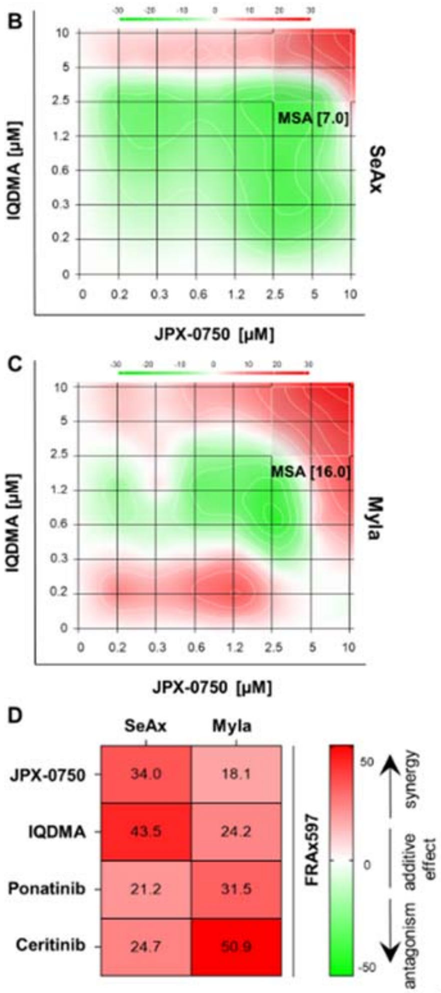

Figure 6B-D in the revised manuscript

Specific Points

1. My main concerns are the patient sample sizes used in the study, which is very small and the lack of in-vivo data, given the premise of the study is to evaluate the usefulness of a drug for inhibiting CTCL growth and survival. Given the multiple genetic aberrations identified in CTCL patients, sampling just six patients to study the effects of specific drugs in-vitro does not provide robust data to make generalized conclusions about its potential efficacy as a targeting strategy for leukemic CTCL. There are six patients included in this study and yet the biochemical and viability assays are only performed on patients 4 and 5 primarily and P6 in one experiment. Why were the others not analyzed and on what criteria were only these two patients chosen for the majority of the experiments with patient samples? As the demographics of this disease is that it affects older individuals, were the healthy control group age matched also? Please provide some more details.

We appreciate the reviewer concerns and have tried to address them by increasing the sample size and performing *in vivo* experiments to obtain more significant patient relevant results.

Thus, we reached out to several international collaborators explaining partly also the larger number of authors on the revised version due to clinical sampling from different centers, where we were able in networking efforts to increase the sample size of the study cohort. Altogether, we were able to collect another 29 additional L-CTCL patient samples, out of which 17 passed the stringent quality control check necessary for whole genome sequencing, resulting in a total of 23 patients (17 + 6 included in the original submission), out of which 74% presented with a *STAT3/5* copy number gain (**Figure 1**, shown on page 12). Out of the 23 patients, we performed drug testing with all three drugs with 15 samples, which contained enough viable PBMCs to perform the drug testing. Our drug screen revealed that indeed the three selected drugs are efficient in killing the patient cells. Furthermore, we discovered that particularly the PAK kinase inhibitor, FRAx597, has better efficacy in L-CTCL cells with *STAT3/5* copy number gains (**Figure 6E**, shown on page 14). Namely, its response inversley correlated with the respective CNA log2 ratios (**Figure 6G-H**, shown on page 14). The exact mechanism of action remains to be further elucidated and lead structure development and optimization towards a clinical grade candidate is required to obtain better inhibitors for arresting aggressive L-CTCL malignant cell growth, where one might be able to move into a Phase Ia clinical trial with such improved drugs, surely going beyond the scope of this pioneering therapy exploration.

Due to varying quality and small amounts of primary patient samples, we could not perform further Western blot and gene expression (RT-qPCR) analysis, since we kept the focus on targeted drug insight, clonality analysis and detailed sequencing. Therefore, the Western blot and RT-qPCR analysis contain the same number of samples as originally submitted (now shown in **Figure 2D-F-RT-qPCR data** and **Figure 2G-Western blot**).

Regarding the matched healthy individuals, we have also included drug testing on healthy PBMCs on both young (H1-H9, **Figure EV4**) and older individuals (H10-H16, **Figure EV4**). While the drugs seem to be relatively safe in younger individuals, the PBMCs isolated from elderly adults had very low IC_{50} values pointing to potential toxicity. Since all of the drugs evaluated are in the preclinical development stage, the toxicity issue has to be addressed towards work for a clinical grade targeted drug, which was not within the scope of this manuscript. We emphasize on these issues thanking the reviewer for insightful comments.

IC_{50} [μM, 48 h]

	JPX-0750	IQDMA	FRAx597
H1	6.78	16.16	3.10
H2	13.26	7.99	<100
H3	6.27	1.73	<100
H4	11.57	1.58	<100
H5	0.94	0.68	3.50
H6	3.51	2.52	<100
H7	9.50	8.02	1.59
H8	9.23	8.75	n.d.
H9	0.80	0.86	0.95
H10	1.54	0.97	1.64
H11	1.05	0.88	0.78
H12	1.22	0.71	1.41
H13	1.65	0.86	1.37
H14	0.95	1.00	0.86
H15	1.28	1.10	1.52

Figure EV4 in the revised manuscript

Finally, the *in vivo* experiment performed with an intradermal xenograft mouse model, demonstrated that the PAK inhibitor, FRAx597, has the most significant impact on reducing tumor growth and malignant cell dissemination, where we would like to again emphasize that lead structures such as JPX-0750 or IQDMA need to be developed further with medicinal chemistry lead structure development efforts to achieve better pharmacodynamics/pharmacokinetics for *in vivo* applications (**Figure 7** and **Figure EV5**, shown on pages 8 and 9, respectively).

2. The western blot depicted in Figure 4B should be quantified and statistics performed so that subtle changes in STAT3/5 protein (especially with IQDMA treatment) can be clearly evaluated. This experiment should also be performed on all the cell lines used in the viability and apoptosis assays, because for instance Hut78 is later used in the viability assay, but is not characterized for its STAT3/5 dose response to IQDMA and JPX0750.

We thank the reviewer for these suggestions to better quantify results and to include statistics, which we acquired now better, where we apologize for not doing so in original submission. The Western blots were now quantified and the effect on STAT3/5 activation and total protein levels is now improved visible (the whole Western blots together with quantification analysis are provided in the ´Source data´ files, as well as highlighted under the respective lane in the respective figures).

The cell lines initially selected for the Western blot analysis upon drug treatment had detectable STAT3/5 activation and total protein STAT3/5 levels. The Hut78 L-CTCL cell line was originally not included in the Western blot analysis due to the very low total STAT3/5 protein level and due to the lack of significant STAT5 tyrosine activation. However, respecting the reviewer request/advice, we have now included this analysis together with densitometry values (**Figure EV2A-D**).

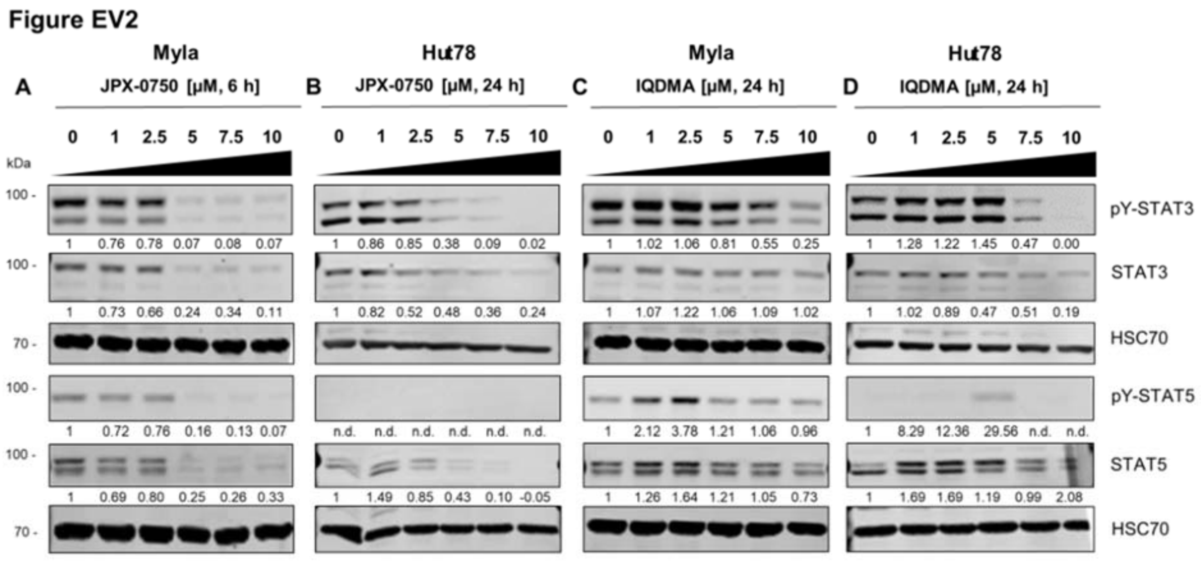

Figure EV2A-D in the revised manuscript

3. The viability and apoptosis experiments shown in Figure 4cd use different concentrations of drugs; apoptosis/necrosis at 2.5uM and viability 0.75-3.12 uM, with significant effects on viability observed at 1.56 uM and we are not shown a "no" treatment control. Why use different doses in the different experiments on the same cell lines? It's very confusing without explanation for why different doses are used, and also why a dose below 2.5uM (for JPX-0750) or 5uM (for IQDMA), which doesn't seem to have any effect of STAT3/5 proteins, has such a significant effect on cell viability and apoptosis?

The different assays were used to depict different effects on cell viability, cell death or the therapeutic window, which we have now better explained in the respective parts of the manuscript and we thank the reviewer for pointing this discrepancy out.

The RealTime-Glo™ Annexin V Apoptosis and Necrosis Assay was used to measure the real-time exposure of phosphatidylserine (PS) on the outer leaflet of cell membranes during the apoptotic process. The kinetics of apoptosis induction was originally performed at 1µM and 2.5µM, but yielded the best results at 2.5µM for both drugs in both cell lines, due to the sensitivity of the assay. It is of note, that the IC_{50} values are 1.34 and 1.26µM after 48h treatment with JPX-0750 in the SeAx and Myla cells, respectively, and 1.63 and 0.69µM after 48h treatment with IQDMA, respectively, as stated in Figure 4A. However, the assay reagent is configured to produce signal intensities proportional to the magnitude of the PS-exposure for up to 48h, as stated in the Promega manualfile:///C:/Users/Sorger/Downloads/RealTimeGlo%20Annexin%20V%20Apoptosis%20and%20Necrosis%20Assay%20Technical%20Manual,%20TM507.pdf), which is why we decided to

go with a higher dose (2.5 µM), to be able to detect the gradual PS-exposure within the 48h time frame.

For the therapeutic window analysis, we wanted to compare the viability of CTCL cells with cells that would normally be present in the skin in CTCL, namely fibroblasts and keratinocytes. For this we used a low dose range starting with the lowest respective IC_{50} value (namely 0.7µM) to the highest (namely 1.6µM) followed by an additional double concentration (3.1µM) to evaluate also the sensitivity of the primary juvenile fibroblasts (JF) and keratinocytes, HaCat cells and evaluate the therapeutic window. We introduced a short explanation into the text and legend as follows:

Text: We compared the viability of primary human fibroblasts (*i.e.* juvenile fibroblasts, JF) and HaCat cells with CTCL cells (Hut78, SeAx and Myla) at a low dose range (*i.e.* 0.7-3µM) and observed that both JF cells and HaCat remain 90% viable at the effective concentrations that kill L-CTCL cells, indicating a therapeutic window for using these drugs (**Figure 4H, I**).

Legend: The effect of **(H)** JPX-0750 and **(I)** IQDMA on the viability of CTCL cells in comparison to viability of primary juvenile fibroblasts (JF) and keratinocytes (HaCat) at a low dose range (0.7-3µM). One experiment, in triplicates, was performed with JF and HaCat cells, and three experiments, in triplicates with the CTCL cells. Statistical significance was calculated by two-way ANOVA with Tukey's multiple comparisons test. P-value: <0.05 (*), <0.01 (**), <0.001 (***), <0.0001 (****).

We hope we were able to better clarify the choice of concentration/experimental design to readers. We addressed the fact why a dose below 2.5µM (for JPX-0750) or 5µM (for IQDMA), which doesn't seem to have any effect of STAT3/5 proteins, has such a significant effect on cell viability and apoptosis, by proving that indeed IQDMA is not a STAT3/5 inhibitor (as shown by the thermal shift fluorescent polarization experiments in **Figure EV5A-B** in the revised version), but a multi-kinase inhibitor which indirectly downregulates STAT3/5 nuclear levels, even though the exact mechanism of action remains to be elucidated.

Figure EV3

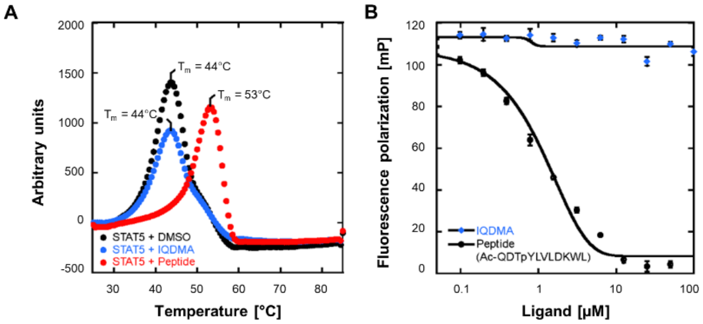

Figure EV5 panel A-B, which were part of the main Figure 5 in the original submission

4. In Figure 5C, again a different dose of IQDMA is used in the kinome analysis. This time the experiment has been done with 10uM IQDMA, at which dose viability and apoptosis seems to be maximally inhibited and from the western blot (fig 4b) the control protein HSC70 also seems to be affected, suggesting that the drug is acting by non-specifically inducing apoptosis at this dose. Therefore, the kinome screen should really be done at a much lower dose (<5uM) to be consistent with the earlier analyses and at where it might be functioning in a more STAT-specific manner. Does IQDMA target all these kinases (and specifically PAK1) at lower doses (<2.5uM), or only a subset?

We thank the reviewer for this insightful comment. As the reviewer pointed out, part of the rationale for the selection of the concentration of IQDMA in the kinome screen was to capture the maximal activity of the compound, and therefore broadly examine any potential targets that were captured. However, we agree with the reviewer, in that examining lower doses would also be important. We carried out a more comprehensive screening approach with a range of concentrations through global proteomics profiling which enabled us to examine IQDMA-specific effects at 1, 2.5 and 10µM in an unbiased and dose-dependent manner in SeAx cells, without limiting the analysis to kinases. We have shown that the differential protein levels in response to increasing IQDMA concentrations are related to multiple cellular processes in metabolism, ribosomal RNA processing/modification, protein translation, and importantly keratinization (**Figure EV3E**, shown on page 17). At the 1 µM and 2.5 µM doses, there was significant downregulation of epithelial proliferation promoters, while the higher doses were associated with downregulation of a STAT3/5 target (namely CCND2) (**Figure 5D-F**, shown on page 17). As previously mentioned, this effect was recapitulated in our Western blot studies, where the direct STAT3/5 targets were only affected

at concentrations higher than the IC_{50} values, further confirming IQDMA is not a direct STAT inhibitor, but impacts STAT3/5 signaling (**Figure 6A**, shown on page 18).

We have therefore amended and extended our conclusion, presenting IQDMA as a broad-range kinase inhibitor, which improves the prospects of potential kinase-targeting drugs in CTCL, to be more consistent with the data presented.

5. The western blot in figure 5E should be repeated multiple times and quantified or use another technique such as Imagestream to get quantitative data, as the data shown here is not convincing in terms of an effect on disruption of STAT nuclear translocation, which only seems to happen at 10uM, whereas all the earlier data seem to show an effect on viability/apoptosis at lower does <2.5uM. As such, together the data in Fig 5E and F would suggest that IQDMA has a generalised inhibitory effect on CTCL viability and apoptosis and is not mediated via inhibition of STAT5/3 (even indirectly).

We have repeated the Western blot and show the densitometry data in the source files, as well as quant. values underneath the respective protein lanes. After optimizing the time-point of treatment for the experiment (namely 24h, in comparison to 18h shown before), we now show that IQDMA is indeed downregulating levels of total and activated STAT5 in the nucleus at 5µM. However, this was not the case with STAT3, where we kept the data originally submitted with 18h treatment and the effect on activated STAT3 is visible at 10µM (**Figure 5B** and **Figure EV3D**, shown on page 16). This data together with the STAT3/5 target Western blots (**Figure 6A**, page 18) and proteomics data (**Figure 5**, page 17) display an effect on STAT3/5 downstream targets that better proves that IQDMA has an indirect effect on STAT3/5 signaling, even though it might be beyond the scope of this revised manuscript work to fully understand the mechanism of action.

6. The experiments in Figure 5E and F should be performed with the PAK1 inhibitor to show its effect on STAT3/5, and to prove that IQDMA is working through PAK1, ideally PAK1 should be knocked out/down and then treated with IQDMA to support the claim that the inhibition of STAT3/5 by IQDMA is mediated through PAK1.

We agree with the reviewer that we did not prove that IQDMA is inhibiting STAT3/5 via PAK kinase, and we have also removed these overstatements throughout the manuscript and we apologize for our misconception there. Our investigation of the mechanism of action of IQDMA revealed that it is not a STAT5 inhibitor, but rather a broad-range multi-kinase inhibitor engaging

with several tyrosine and serine/threonine kinases, one of which one was also PAK2. Prompted by the already reported role of the PAK kinase on STAT5 signaling, we investigated the effect of IQDMA on STAT3/5 nuclear levels and observed that it downregulates STAT5 activated and total nuclear levels at 5μM and activated STAT3 at 10μM (**Figure 5B and Figure EV3D**, page 16) (1, 2).

We therefore now describe IQDMA in the manuscript more cautious in the following way: IQDMA enabled us to select potential kinase drugs acting in CTCL cells with *STAT3/5* gains, out of which the most promising one seems to be the PAK inhibitor, even though the mechanism of action remains to be further elucidated potentially by genetic experiments knocking down or genetically deleting PAK kinase(s). We found that both IQDMA and FRAx597 suppress STAT3/5 nuclear levels and downstream signaling. Furthermore, they were both effective in primary patient cells *in vitro*, as well as in reducing tumor growth in a mouse xenograft model *in vivo*.

7. Figure 6A shows very varied effects on some STAT3/5 regulated target genes, with some being inhibited with increasing doses, while some not at all, and others in between these two scenarios. Again, these blots should be quantified by densitometry to provide significance to the results.

We have provided densitometry for all respective Western blots and the whole blots are shown in the source data files. We would also like to state that we are currently showing a new more representative blot for CDK6 and PIM1 protein levels. Additionally, due to clarity and relevance to signaling, as well as antibody/reproducibility issues, we removed the Bcl-2 and Bcl-x_L blots.

Minor point:

Figure legend to Fig 3b, please describe what HH is.

We thank the reviewer for this point. It was a typo mistake and the cell line that is shown on the Western blot was H9, a subclone of Hut78. However, at the time of the submission we did not have the respective drug testing data. We have now performed all the analysis also for this L-CTCL cell line and included it in the drug screens, aCGH analysis and also performed xenografting with it.

Additionally, we would like to state that the phospho-serine-STAT5A / STAT5A / STATB Western blot submitted in the original Figure S3 (now Appendix Figure S4) was removed since we were not able to reproduce the findings and include all cell lines into the analysis due to antibody issues. A new representative pY-STAT1 / STAT1 Western blot is now shown in main **Figure 3** (shown here on page 13).

Referee #3 (Comments on Novelty/Model System for Author):

L-CTCL patients with 17q copy number alterations show enhanced STAT3/5 expression which in turn correlates with disease severity. The authors hypothesize that STAT3/5 targeting could be beneficial in arresting L-CTCL development. They provide experimental support for this hypothesis using human cell models, and the analyses performed by the authors in this regard are adequate. The authors then continue to validate the action of IQDMA as a multi-kinase inhibitor and CTCL cell death inducer, followed by an exploration of the apoptosis mechanism.

PAK2 was determined to be one of the kinases whose activity was blocked by IQDMA. This was followed by the observation that the PAK2 inhibitor drug was the only one with activity specific to the STAT3/5 gain, as PAK2 regulates nuclear shuttling of STAT5. They conclude that L-CTCL cells can be targeted by blocking the STAT3/5 pathway directly or indirectly, through dual STAT inhibitors or PAK2 inhibitors respectively, to similar extents.

We thank the reviewer for recognizing the potential merit of this study. In order to potentiate our findings, we increased the sample size of the study cohort. Our efforts resulted in acquiring 29 additional L-CTCL patient samples, out of which 17 passed the quality control check necessary for whole genome sequencing, resulting in a total of 23 patients (17 + 6 included in the original submission), out of which 74% presented with a *STAT3/5* copy number gain (**Figure 1**, shown on page 12), confirming our initial findings. Out of the 23 patients, we performed drug testing with all three drugs with 15 samples, which contained enough viable PBMCs to perform the drug testing. Our drug screen revealed that indeed the three selected drugs are efficient in killing patient cells and demonstrated that the PAK inhibitor has better efficiency in cells with *STAT3/5* copy number gains (**Figure 6E**, page 14). Namely, its response correlated with the respective CNA $\log_2$ ratios (**Figure 6G-H**, page 14). In addition, the PAK inhibitor was also very potent *in vivo* in reducing tumor growth and malignant cell dissemination in an intradermal mouse xenograft model. The exact mechanism of action remains to be further elucidated and lead structure development is required to obtain better inhibitors for arresting aggressive L-CTCL malignant cell growth.

Referee #3 (Remarks for Author):

The manuscript is clear in analysis and content, but here are some minor comments to help improve the quality further.

1) More details need to be included in the Methods section to increase clarity for the readers. Certain areas include a) TCR-beta repertoire -- expand on technique, method, and identification of clones, b) CNA identification -- expand on ichorCNA and parameters using for identifying CNAs, p/q-values of alterations, etc. c) Synergy screen -- explain the purpose of ZIP model and cNMF algorithm, what 4 parameters in logistic regression, etc.. d) Expression analysis -- describe the Caprini lymphoma dataset, exactly what was extracted from Oncomine platform and the original paper, what was the Spearman correlation used for, and so on.

We thank the reviewer for this comment to improve our manuscript. We have included the requested explanations into the Materials and Methods part, as well as expanded description to it where necessary to the figure legends or result text body, respectively, as outlined in detail also below.

1. **TCR-beta repertoire**

Identification of malignant clones by TCR Vβ repertoire and sorting

Malignant T-cell clones were identified from L-CTCL patients PBMCs using the TCR Vβ Repertoire Kit (Beckman Coulter #PN IM3497, USA). The TCR Kit is a multiparametric analysis tool designed for quantitative determination of the TCR Vβ repertoire of human T lymphocytes by flow cytometry. The kit is composed of TCR Vβ antibodies corresponding to 24 different specificities and allows about 70% coverage of the normal human TCR Vβ repertoire. Upon identification of the malignant clone, the respective $CD3^{+}TCRV\beta^{+}$ malignant clones were sorted from patient PBMCs using fluorescence-activated cell sorting. For P1-P6, $CD19^{+}$ B cells were also sorted from the same samples for control purposes.

Following is a representative image of a TCR vβ analyzed patient sample, which was also included as Appendix Figure S1:

Appendix Figure S1

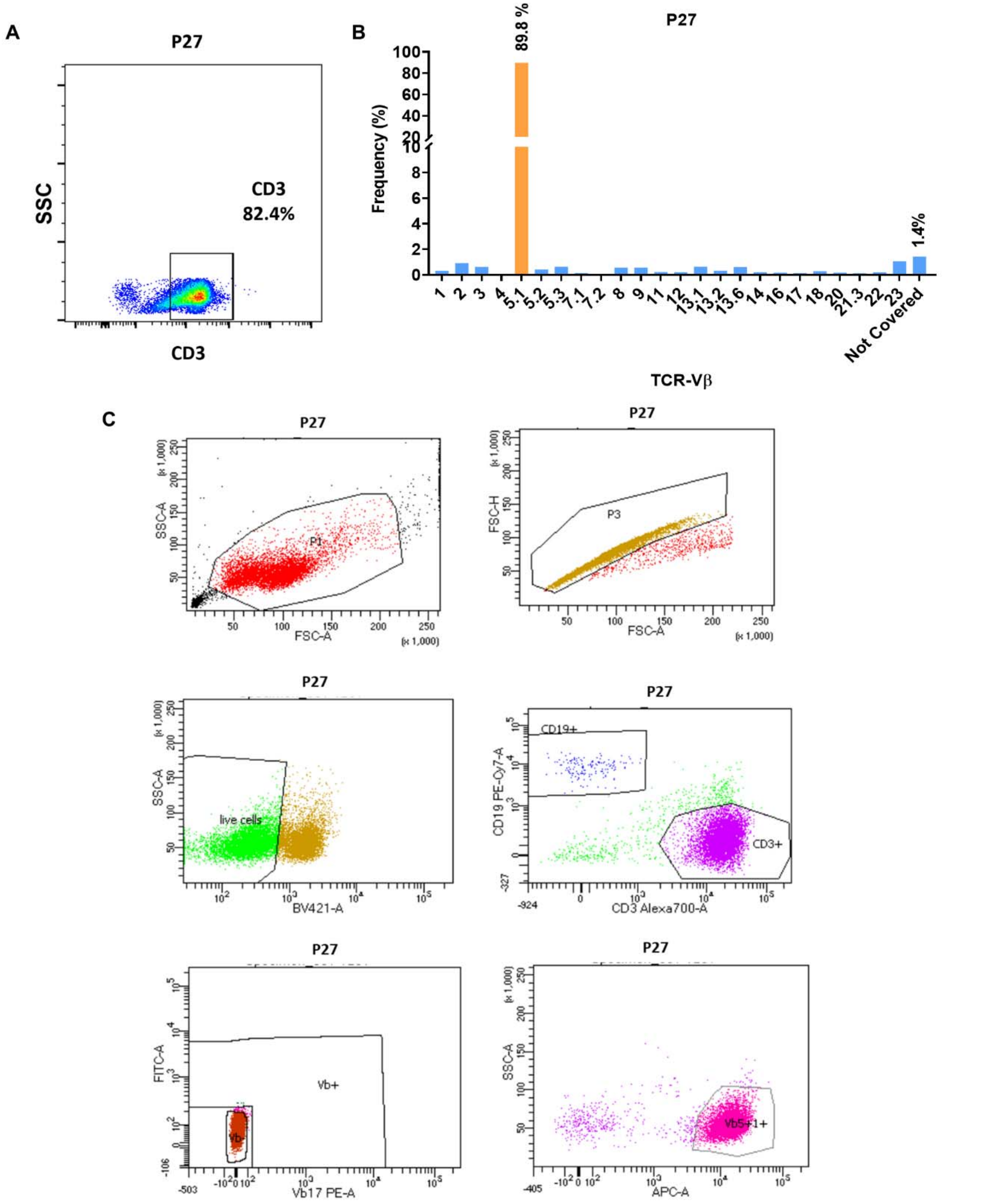

Appendix Figure S1. Identification of the TCRVβ⁺ malignant clonal population in L-CTCL patients.

A-C Patient PBMCs are subjected to live/dead staining, CD3 staining and TCRVβ staining, a multi-parametric analysis tool designed for quantitative determination of the TCRVβ repertoire of human T lymphocytes by flow cytometry. (**A**) representative image of the CD3 gating strategy. (**B**) TCRVβ clonal frequency analysis plot. A dominant clonal population (TCRVβ 5.1) is identified from P27, shown as representative. (**C**) A representative figure from P27 showing the flow cytometric gating strategy for sorting/enrichment of the $CD3^+$ $TCRV\beta^+$ clonal population.

2. CNA identification

For chromosomal copy number identification from shallow whole genome sequencing (sWGS) data (P1-P6) we used the broad institute ichorCNA program which is a tool for estimating the fraction of tumor in cell-free DNA from sWGS (0.1x coverage). ichorCNA uses a probabilistic model, implemented as a hidden Markov model (HMM), to simultaneously segment the genome, predict large-scale copy number alterations, and also estimate the tumor fraction of an sWGS sample (ULP-WGS). ichorCNA is optimized for low coverage (~0.1x) sequencing of samples and has been benchmarked using patient and healthy donor cfDNA samples. For the WGS sequencing above 1X coverage we used the Gencove cloud service for mapping to the Human low-pass GRCh38 v1.2. After mapping, an imputated variant calling analysis was performed on the Gencove analysis platform to produce SNPs and CNVs using the configuration "Human low-pass GRCh38 v1.2. (Gencove capability id: dc5fc85b-1a9a-4198-bb5f-7b61fc171e33). This imputation reference panel is comprised of the phased release of genotype calls from the New York Genome Center's resequencing efforts of individuals from the 1000 Genomes Project. The panel is comprised of 3202 individuals, including the original 2504 and an additional 798 relatives. The threshold for CNA evaluation was set to +/-0.1 $\log_2$ ratio, where +0.1 represents gain, and -0.1 loss.

3. Synergy screen

The analysis was performed using the SynergyFinder 2.0 web application (3). The degree of synergy was quantified using the Zero interaction potency (ZIP) model, that captures the drug interaction relationships by comparing the change in the potency of the dose–response curves between individual drugs and their combinations (4). The ZIP model enabled calculation of the most synergistic (MSA) area score, which represents the most synergistic 3-by-3 dose-window.

The Zero interaction potency model was applied to quantify the degree of synergy, according to which an MSA score below -10 indicates that drugs are antagonistic, a score between -10 and 10 indicates that 2 drugs are additive, while a score above 10 indicates a synergistic effect. The four-parameter logistic regression (LL4) was used as the curve-fitting algorithm (3). ~~The cNMF algorithm was applied to detect and replace outlier measurements when necessary (5).~~ All assays were repeated in two biological replicates.

We omitted the analysis with the cNMF algorithm for better reproducibility.

4. **Expression/Oncomine analysis**

Expression data were extracted from the Oncomine™ Platform (Thermo Fisher, Ann Arbor, MI) Caprini Lymphoma dataset, which includes 32 Sezary syndrome patients (6) (www.oncomine.org). In the study, the $CD3^+$ mononuclear cells from blood of patients were subjected to the Affymetrix HG-U133A assay. We extracted $CD3^+$ clonality and genetic data, namely information on 17q status, from the original study. Only patients with 17q gains were included into the analysis. Spearman correlation analysis (nonparametric) was used to correlate the percentage of clonal $CD3^+$ cells and the respective STAT3/5A/5B expression values to obtain the r and p values. Simple linear regression was used to plot the graph using GraphPad Prism 8.4.2 software.

2) Include test identity, metrics, p-values in the main results section whenever a statistical comparison/evaluation is made. This improves clarity for the reader and provides context to how conclusions were drawn in the text.

We thank the reviewer for the comment and have included the statistical metrics throughout the manuscript text, Materials and Methods section and in the figures/figure legends.

Collectively, we wish to thank the reviewers and editorial team for their work and efforts, giving us objective critics and advice, where we hope that we have answered all relevant points in specific detail. We hope that we were able with your help to improve the revised version of the manuscript carefully, also with approval and comments obtained prior to uploading from all authors involved in this study.

REFERENCES:

1. Berger A, Hoelbl-Kovacic A, Bourgeais J, Hoefling L, Warsch W, Grundschober E, et al. PAK-dependent STAT5 serine phosphorylation is required for BCR-ABL-induced leukemogenesis. Leukemia. 2014;28(3):629-41.
2. Chatterjee A, Ghosh J, Ramdas B, Mali RS, Martin H, Kobayashi M, et al. Regulation of Stat5 by FAK and PAK1 in Oncogenic FLT3- and KIT-Driven Leukemogenesis. Cell Rep. 2014;9(4):1333-48.
3. Ianevski A, Giri AK, Aittokallio T. SynergyFinder 2.0: visual analytics of multi-drug combination synergies. Nucleic Acids Research. 2020;48(W1):W488-W93.
4. Yadav B, Wennerberg K, Aittokallio T, Tang J. Searching for Drug Synergy in Complex Dose-Response Landscapes Using an Interaction Potency Model. Comput Struct Biotechnol J. 2015;13:504-13.
5. Ianevski A, Giri AK, Gautam P, Kononov A, Potdar S, Saarela J, et al. Prediction of drug combination effects with a minimal set of experiments. Nature Machine Intelligence. 2019;1(12):568-77.
6. Caprini E, Cristofoletti C, Arcelli D, Fadda P, Citterich MH, Sampogna F, et al. Identification of Key Regions and Genes Important in the Pathogenesis of Sézary Syndrome by Combining Genomic and Expression Microarrays. Cancer Research. 2009;69(21):8438-46.

12th Sep 2022

Dear Prof. Moriggl,

Thank you for the submission of your revised manuscript to EMBO Molecular Medicine and please accept my apologies for the delay in getting back to you as the reports came when I was on annual leave. Moreover, as you will see from the reports below, the referees have diverging views on your revised manuscript, and I further consulted with them and discussed with my colleagues before making a decision.
Indeed, while referee #1 is now supportive of publication, referee #3 is rather negative and criticizes the lack of novelty of the study. Given the extent of the revisions that were performed, and based on the report from referee #1, we nevertheless decided to proceed further with your manuscript, and I am therefore pleased to inform you that we will be able to accept your manuscript once the following points will be addressed:

1/ Referees' reports:
- Please address the points from referee #3 in a detailed rebuttal letter, and clearly highlight the novelty of your study in your main manuscript text.

2/ Main manuscript text:
- Please accept the changes, remove the highlights in yellow, and only keep in track changes mode any new modification.
- Thank you for providing a Data Availability section. Please add an URL to access datasets. Please note that the accession number you provided for proteomics data is not valid.

3/ Figures and Appendix:
- Please provide exact p values in all figures or in their legends (i.e. Fig 4H and Fig. EV2E). Some people found that to keep the figures clear, providing a supplemental table with all exact p-values was preferable. You are welcome to do this if you want to.
- Data from figure 1A were reused in figure EV2, and similarly data from figure 3A were reused in figure S4. This should be mentioned in the figure legends.

4/ An ORCID identifier is missing for P. Gunning.

5/ Thank you for providing a synopsis. Please add 3 to 5 one-sentences bullet points that summarize the key new findings. Please resize your nice visual abstract to 550px wide x 300-600px high and make sure that the text remains legible.

We note that you agree with the publication of the RPF.

I look forward to receiving your revised manuscript.

With kind regards,

Lise Roth

Lise Roth, PhD

Senior Editor

EMBO Molecular Medicine

To submit your manuscript, please follow this link: Link Not Available

***** Reviewer's comments *****

Referee #1 (Comments on Novelty/Model System for Author):

Extensive corrections made that have removed any lack of clarity or overstatements and greatly strengthened the findings in the revised manuscript.

Referee #1 (Remarks for Author):

Thank you for addressing all the points satisfactorily and all the extra experimentation, especially including the new in-vivo mouse data that greatly strengthens the validity of the inhibitors. Good job!

Referee #3 (Comments on Novelty/Model System for Author):

The authors have received feedback and made attempts to address all the questions raised by the reviewers, but unfortunately, in light of the new results, I believe there is now a lack of a novelty component to their work thereby making their paper unsuitable for publication. The main novel premise of their submitted manuscript was identifying and proving the efficacy of IQDMA in direct/indirect inhibition of STAT3/5, but this isn't statistically significant in their new in-vivo analyses with expanded samples. The remaining premises claimed in their paper (as listed below) are not novel to the field, and have already been shown in prior publications.

1) Result section 1 - Frequent STAT3/5 gains in CTCL. This has been previously known and established with rigor.
2) Result section 2 - STAT3/5 copy number gains correlate with increased clonal proliferation and result in STAT3/5 overexpression and hyperactivation in L-CTCL - The authors have added a lot of new analyses here, but it still addresses aspects that aren't novel to the field and not the main premise of the paper.
3) Result section 3 emphasizes the usefulness of cell lines as models for in-vitro testing; while this is well received it isn't a replacement for the requested affirmation of efficacy from in-vivo studies necessary for therapeutic translation of tested drugs.
4) Next few sections on tests on cell-lines show the efficacy of all three drugs; same comment as above.
5) Last few sections comment on the efficacy of FRAx597 in-vivo and the overall usefulness of PAK inhibition -- this drug is already a known PAK inhibitor with prior in vivo efficacy support, and the findings here are not novel.

Referee #3 (Remarks for Author):

I recommend that the authors re-write to focus the article on what was truly novel and statistically significant in their study.

Reply to EMM-2021-15200-V2 Decision Letter

Point-by-point response:

Referee #1 (Comments on Novelty/Model System for Author):

Extensive corrections made that have removed any lack of clarity or overstatements and greatly strengthened the findings in the revised manuscript.

Referee #1 (Remarks for Author):

Thank you for addressing all the points satisfactorily and all the extra experimentation, especially including the new in-vivo mouse data that greatly strengthens the validity of the inhibitors. Good job!

We thank the reviewer for providing constructive criticism and for suggesting experiments that made the paper significantly better during the process of the revision.

Referee #3 (Comments on Novelty/Model System for Author):

The authors have received feedback and made attempts to address all the questions raised by the reviewers, but unfortunately, in light of the new results, I believe there is now a lack of a novelty component to their work thereby making their paper unsuitable for publication. The main novel premise of their submitted manuscript was identifying and proving the efficacy of IQDMA in direct/indirect inhibition of STAT3/5, but this isn't statistically significant in their new in-vivo analyses with expanded samples.

CTCL is a very rare malignancy affecting ~1/100 000 individuals, out of which 10% will progress into the highly aggressive leukemic L-CTCL phenotype, displaying peripheral organ and blood malignant cell dissemination (Bagherani & Smoller, 2016). Given the struggle that CTCL patients face due to high symptomatic burden, inefficient treatments, and considerable mortality, the main premise of the paper was to explore novel targeted therapy options particularly for advanced and leukemic CTCL (L-CTCL).

In order to identify most common disease aberrations and thereby identify the targetable nodes, it was necessary to characterize all patient samples using whole exome/genome sequencing

(WES/WGS). We initially performed analysis on 6 L-CTCL patients, where the genomic analysis appointed to 17q (*STAT3/5*) gain as one of the most common genomic abnormalities (**Appendix Figure S2** in the revised manuscript). This finding was supported during the process of revision, where we included an additional 17 samples that passed the stringent quality control check necessary for WGS, resulting in a total of 23 patients (17 + 6 included in the original submission), out of which 74% (n=17/23) presented now with a *STAT3/5* copy number gain (**Figure 1**). We acknowledge that previous studies also detected chromosome 17q copy number alterations (CNA) (Caprini *et al*, 2009; Choi *et al*, 2015; da Silva Almeida *et al*, 2015; Fanok *et al*, 2018; Kiel *et al*, 2015; Lin *et al*, 2012; Prasad *et al*, 2016; van Doorn *et al*, 2009; Wang *et al*, 2015; Woollard *et al*, 2016). However, none of the previous studies addressed the efficacy of direct STAT3/5 targeting and all studies published so far despite genetic evidence lack any proof of concept that STAT3 and/or STAT5 are oncoproteins. This is particularly important since recurrent mutations in hotspots or STAT3, STAT5A or STAT5B are not found, but they are increased in expression and we also provide evidence that they are constitutively activated due to impaired negative feedback controls and STAT1 antagonism. Thus, we here show for the first time, that the three STAT3, STAT5A and STAT5B transcription factors act oncogenic in L-CTCL. We want to point out to the reviewer that STAT3 or STAT5A or STAT5B can be oncogene or tumor suppressor and that is mutational context as well as cell type dependent and not clear for L-CTCL. Thus, to define these three key transcription factors for lymphocyte function and cell fate as oncoproteins in L-CTCL is a novelty confirming and extending, plus explaining tumor biology consequences of genetic findings by others. We do cite the literature of others and it was proposed to be linked to inadequate responses to already used targeted therapies, such as HDACi (Eriksen *et al*, 2001; Fantin *et al*, 2008).

Upon applying a focused drug screen using confirmed and putative STAT3/5 inhibitors, we observed that novel direct STAT3/5 small molecule degraders were as effective as IQDMA, a potent drug with an unknown mechanism of action (Chien *et al*, 2008; Yang *et al*, 2008), advertised as a STAT5 inhibitor (https://www.abcam.com/iqdma-cell-permeable-stat5-inhibitor-ab141192.html, https://www.sigmaaldrich.com/AT/en/product/mm/420294) (**Figure 4A**).
In order to investigate the mechanism of action of IQDMA, we explored its target engagement thereby identifying several kinases acting upstream of STAT3/5 (ABL, ALK, TYK2, JAK3, B-RAF, PAK2, PIM3), indicating that its effect was indirect (**Figure 5A**). Acknowledging the

power of multikinase inhibition for personalized therapy development (Garuti *et al*, 2015), we compared IQDMA with already approved multikinase inhibitors, but also with more specific kinase inhibitors, such as JAK and PAK inhibitors (**Figure 5G**). We observed that JAK kinase inhibitors were ineffective, whereas multikinase inhibitors such as ceritinib, ponatinib and sorafenib were more effective, and interestingly, the PAK kinase inhibitor, FRAx597, was specifically acting in cells with *STAT3/5* gains (**Figure 5G**).

We further observed that both IQDMA and FRAx597 suppress STAT3/5 nuclear levels (**Figure 5B, EV3D** and **Figure EV3F**, respectively) and downstream signaling (**Figure 6A**). Moreover, they were both effective in primary patient cells *in vitro* (**Figure 6E**), as well as in reducing tumor size, as measured by tumor perimeter, in a mouse xenograft model *in vivo* (**Figure 7E, F**). However, the PAK inhibitor exhibited marked specificity for patient cells carrying *STAT3/5* gains (**Figure 6A**), eluding to a key role of the PAK kinase in STAT3/5-induced oncogene induction in L-CTCL, likely regulated by STAT3/5 nuclear shuttling.

In conclusion, our manuscript shows that dual STAT3/5 degradation through small molecular covalent inhibitors is potent *in vitro*, but blocking of upstream kinase action, particularly PAK kinase, is necessary *in vivo* and could represent a promising therapeutic strategy to eradicate malignant L-CTCL cells carrying *STAT3/5* gains.

Therefore, IQDMA enabled selection of potential kinase drugs acting in CTCL cells with *STAT3/5* gains, out of which the most promising one seems to be the PAK inhibitor, that suppresses STAT3/5 nuclear levels.

The remaining premises claimed in their paper (as listed below) are not novel to the field and have already been shown in prior publications.

1) ***Result section 1 - Frequent STAT3/5 gains in CTCL. This has been previously known and established with rigor.***

We acknowledge that *STAT3/5* gain was already reported as a frequent abnormality of CTCL and that STAT3/5 hyperactivation was suggested to contribute to disease progression (Eriksen *et al.*, 2001; Netchiporouk *et al*, 2014), but a genetic claim is not an experimental proof. However, our study also elucidated the genetic contribution of simultaneous oncogene gain and tumor suppressor loss in the pathogenesis of progressed CTCL and we used pharmacologic targeting for

proof, plus multiple model cell line systems in a unique fashion to illuminate driver gene context in L-CTCL. Namely, through aCGH or patient sample genetic analysis shown in the revised manuscript and never displayed before in these cell lines or described in L-CTCL patient samples we noted key genetic changes in tumor suppressors co-occurring with *STAT3/5* gains. *STAT1* copy number losses were present in 41% (n = 7/17) and *SOCS1* losses in 18% of samples (n = 3/17; **Figure 1B-C**). Genetic deletion of *SOCS1*, as a negative regulator of JAK-STAT signaling has recently been detected in skin biopsies of patients with tumor-stage mycosis fungoides (Bastidas Torres *et al*, 2018). However, loss of *STAT1*, as a negative regulator of STAT3/5, co-occuring with *STAT3/5* gain in L-CTCL is a novel concept for disease pathogenesis and supports the generation of extremely aggressive CTCL cells resistant to therapy, as previously shown with generation of IFNα-resistant Hut78 cells that lacked STAT1 expression (Sun *et al*, 1998). Even more significant is the virtual absence of any significant SOCS1 protein expression that we also show in the revised manuscript versus control, arguing possibly for either *SOCS1* locus methylation as observed frequent in liver cancer for example or for SOCS1 degradation, where future studies need to investigate that further. To the best of our knowledge, neither genetic deletion of STAT1, nor lost SOCS1 negative feedback control was associated with L-CTCL and our work highlights the need for STAT3/5-directed therapies which was the focus since restoring tumor suppressor function is much more difficult.

2) ***Result section 2 - STAT3/5 copy number gains correlate with increased clonal proliferation and result in STAT3/5 overexpression and hyperactivation in L-CTCL - The authors have added a lot of new analyses here, but it still addresses aspects that aren't novel to the field and not the main premise of the paper***.

We regard this as a side finding and correlative analysis, but the significance of a lymphoproliferation with a clonal expansion for disease manifestation can be also seen in autoimmunity e.g. affecting children with somatic or germline *JAK-STAT-SOCS* mutations, really incompletely understood why that would lead to a clonal T-cell dominance, but relevant for the patient immune system consequence. Thus, we are probably the first to show a direct correlation between *STAT3/5* copy number gain and $CD3^+$ T-cell blood clonality in patients, which we did not discuss due to space constrains in full detail, but we believe this side finding has also sufficient novelty and could be a relevant clinical feature of L-CTCL etiology. Specifically, we

show that high STAT3/5 copy number gains (log_2 ratio) significantly positively correlate with the percentage of clonal $CD3^+$ malignant cells detected in peripheral blood of L-CTCL patients (**Figure 2A-C**). These findings suggest that enhanced oncogenic STAT3/5 activation could be an important disease-driving event. This is difficult to prove without a time-dependent analysis of malignant T-cell clones and tracking of patients from the time-point of diagnosis to the time-point of progression detection, bringing also into light the difficult diagnosis of CTCL, where patients are misclassified as benign inflammatory dermatoses (BID) (Bagherani & Smoller, 2016). For the majority of cases, it takes an average of 6 years from disease onset until correct diagnosis (Bagherani & Smoller, 2016; Diamandidou *et al*, 1996), due to lack of proper diagnostic methods/tools. Identifying malignant cells in the peripheral blood of patients with CTCL is a critical marker for detecting Sézary syndrome in early stages and determining prognosis (Gibson *et al*, 2016; Kirsch *et al*, 2015). So far Ki67, CD34, and AgNORs, as parameters of cell proliferation and angiogenesis, as used as markers for CTCL progression (Maj *et al*, 2015), however there are no markers guiding treatment-decisions. We propose that clonal T-cell expansion paired with detection of *STAT3/5* gains and co-occuring *STAT1/SOCS-1* losses upon peripheral blood clone identification could be more meaningful to help guide treatment decisions and frequency of follow-up assessment to ultimately improve patient outcomes.

3) ***Result section 3 emphasizes the usefulness of cell lines as models for in-vitro testing; while this is well received it isn't a replacement for the requested affirmation of efficacy from in-vivo studies necessary for therapeutic translation of tested drugs.***
4) ***Next few sections on tests on cell-lines show the efficacy of all three drugs; same comment as above.***

These are not really two separate points in novelty aspects and the reviewer repeats partly a biased view of usefulness of model cell systems. As many studies in EMBO MOL MED are published using disease relevant models it is true that we also have used disease relevant cell lines for the selection of the most potent/appropriate drug evaluations or for genetic hallmark analysis and biochemical protein or target analysis. Here, we were careful that we used not only two cell lines, but five to be more representative and we profiled these models also for the first time in detail genetically, where we could verify that they represent a surrogate model cell system. We concluded that SeAx cells are most closest to a cytokine dependent L-CTCL clonal expansion with copy number gain on #17q, but they fail to make L-CTCL in mice simply due to

common gamma chain cytokine dependence, which emphasises that JAK1/3 action will be part of the vulnerable kinase signalling node in L-CTCL, which future studies could mimic better using e.g. a humanized NSG mouse with human common gamma chain cytokine expression. Therefore, we aimed to validate that several of the cell lines used are representative of L-CTCL patient samples and can form leukemia in mice or a s.c. tumor when intradermally injected. In addition, we have also done extensive (ex)-vivo testing with patient-derived primary PBMCs to substantiate the efficacy of the drugs in the patient samples. Indeed, our study cohort for drug testing is one of the largest, among published studies on drug testing in primary L-CTCL patient cells, but we also observed mixed results or imperfect targeting efficacy. This is particularly to be emphasized when the rarity of the disease is considered in association with genetic complexity and individual differences in model cell systems used, detailed very much in the revised manuscript. The other so far published studies only used CTCL established cell lines for drug testing (da Silva Almeida *et al.*, 2015; Fanok *et al.*, 2018; Kiel *et al.*, 2015; McGirt *et al*, 2015). We have isolated and tested cells from a total of 15 L-CTCL patients (**Figure 6E**), together with thorough genomic characterization which helped to draw better conclusions. The focused drug testing with patient samples elucidated the effectiveness of all drugs in patient samples, namely of the small molecule STAT3/5 degrader, JPX-0750; IQDMA and the PAK inhibitor, FRAx597 (**Figure 6E**). It also elucidated the selectiveness of the PAK inhibitor towards cells with *STAT3/5* gain (**Figure 6E**), where the response inversely correlated with the respective CNA $\log_2$ ratios (**Figure 6G-H**).

In addition, while all the drugs worked *in vitro*, our *in vivo* studies show that indeed (multi)kinase inhibition is necessary *in vivo*, where IQDMA and FRAx597 significantly reduced tumor size in an intradermal mouse xenograft model, as measured by tumor perimeter (**Figure 7E, F**). Analysis of both tumor size and tumor weight was included due to specific flat shape of the tumors, and tumor weight was thereby significantly reduced only by FRAx597 (**Figure 7C, D**). Both drugs reduced malignant cell dissemination to the lymph nodes, even though this was only significant for FRAx597 (**Figure EV5A**).

5) **Last few sections comment on the efficacy of FRAx597 in-vivo and the overall usefulness of PAK inhibition -- this drug is already a known PAK inhibitor with prior in vivo efficacy support, and the findings here are not novel.**

Even though the observation that FRAx597 is a PAK inhibitor and that it is effective *in vivo* has previously been shown in Schwannomas (Licciulli *et al*, 2013) and prostate cancer (Wang *et al*, 2020), the effectiveness of PAK inhibition in CTCL has only been moderately investigated (Wang *et al*, 2018; Wang *et al*, 2019). Confirmatory of our premises, PAK-silenced CTCL cells exhibited spontaneous apoptosis *in vitro* and a decreased rate of tumor growth in comparison to control cells *in vivo* (Wang *et al.*, 2018). We did not find any study using PAK kinase inhibitors for CTCL *ex vivo* or *in vivo*. Therefore, even though the drug that we used in our study is not novel and has been preclinically tested in other cancer entities, this is the first study testing the effectiveness of PAK kinase inhibition on primary patient L-CTCL samples *ex-vivo* and in an intradermal xenograft model *in vivo*, where we conclude the overstatement of a negative novelty critic of the reviewer is not justified and could have been raised already upon original submission.

Moreover, the fact that Hut78 and Myla cells displayed very different in vivo tumor cell growth upon intradermal xenografting in NSG mice and responded differently to drug treatment, indicates that indeed for some L-CTCL patients a combination kinase therapy might be necessary, as we also briefly explored here and show that FRAx597 exhibits synergy in combination with JPX-0750, IQDMA, ceritinib and ponatinib (**Figure 6D**), providing the possibility of a combined treatment, that remains to be further tested for L-CTCL.

Referee #3 (Remarks for Author):

I recommend that the authors re-write to focus the article on what was truly novel and statistically significant in their study.

In order to emphasize the novelty aspects and make the manuscript better understandable and easier to follow for the readers of EMBO MOL MED, we suggest the renaming/changing of the following headlining to account a bit better for novelty aspects worked out together (as marked in yellow):

1) *STAT3/5* copy number gains are frequent in L-CTCL and co-occur with copy number loss of *STAT1/SOCS1*

2) Elevated *STAT3/5* copy number and expression levels correlate with increased clonal proliferation in L-CTCL ~~and result in STAT3/5 overexpression and hyperactivation in L-CTCL~~
3) ~~Established CTCL cell lines carrying *STAT3/5* gains can be employed as patient-like models for *in vitro* testing recapitulate genomic characteristic of~~
4) Pharmacologic targeting of STAT3/5 using direct small molecule degraders and IQDMA is effective *in vitro*
5) IQDMA is a broad-range multi-kinase inhibitor that suppresses STAT3/5 nuclear levels
6) Upstream kinase inhibition, particularly of the PAK kinase, is effective in L-CTCL and suppresses STAT3/5 downstream signaling
7) PAK kinase inhibition is specific for L-CTCL patient cells carrying *STAT3/5* gains *ex vivo*
8) ~~The PAK inhibitor FRAx597 blocks STAT3/5 downstream signaling and eradicates L-CTCL patient cells carrying STAT3/5 gains~~
9) ~~PAK inhibition is highly effective in an intradermal L-CTCL xenograft mouse model~~ Upstream (multi)kinase inhibition attenuates L-CTCL growth in xenograft mouse model

References:

Bagherani N, Smoller BR (2016) An overview of cutaneous T cell lymphomas. *F1000Res* 5

Bastidas Torres AN, Cats D, Mei H, Szuhai K, Willemze R, Vermeer MH, Tensen CP (2018) Genomic analysis reveals recurrent deletion of JAK-STAT signaling inhibitors HNRNPK and SOCS1 in mycosis fungoides. *Genes, Chromosomes and Cancer* 57: 653-664

Caprini E, Cristofoletti C, Arcelli D, Fadda P, Citterich MH, Sampogna F, Magrelli A, Censi F, Torreri P, Frontani M *et al* (2009) Identification of Key Regions and Genes Important in the Pathogenesis of Sézary Syndrome by Combining Genomic and Expression Microarrays. *Cancer Research* 69: 8438-8446

Chien C-M, Yang S-H, Lin K-L, Chen Y-L, Chang L-S, Lin S-R (2008) Novel indoloquinoline derivative, IQDMA, suppresses STAT5 phosphorylation and induces apoptosis in HL-60 cells. *Chemico-biological interactions* 176: 40-47

Choi J, Goh G, Walradt T, Hong BS, Bunick CG, Chen K, Bjornson RD, Maman Y, Wang T, Tordoff J (2015) Genomic landscape of cutaneous T cell lymphoma. *Nature genetics* 47: 1011-1019

da Silva Almeida AC, Abate F, Khiabanian H, Martinez-Escala E, Guitart J, Tensen CP, Vermeer MH, Rabadan R, Ferrando A, Palomero T (2015) The mutational landscape of cutaneous T cell lymphoma and Sézary syndrome. *Nat Genet* 47: 1465-1470

Diamandidou E, Cohen PR, Kurzrock R (1996) Mycosis fungoides and Sezary syndrome. *Blood* 88: 2385-2409

Eriksen K, Kaltoft K, Mikkelsen G, Nielsen M, Zhang Q, Geisler C, Nissen M, Röpke C, Wasik M, Ødum N (2001) Constitutive STAT3-activation in Sezary syndrome: tyrphostin AG490 inhibits STAT3-activation, interleukin-2 receptor expression and growth of leukemic Sezary cells. *Leukemia* 15: 787

Fanok MH, Sun A, Fogli LK, Narendran V, Eckstein M, Kannan K, Dolgalev I, Lazaris C, Heguy A, Laird ME (2018) Role of dysregulated cytokine signaling and bacterial triggers in the pathogenesis of cutaneous T-cell lymphoma. *Journal of Investigative Dermatology* 138: 1116-1125

Fantin VR, Loboda A, Paweletz CP, Hendrickson RC, Pierce JW, Roth JA, Li L, Gooden F, Korenchuk S, Hou XS (2008) Constitutive activation of signal transducers and activators of transcription predicts vorinostat resistance in cutaneous T-cell lymphoma. *Cancer research* 68: 3785-3794

Garuti L, Roberti M, Bottegoni G (2015) Multi-kinase inhibitors. *Curr Med Chem* 22: 695-712

Gibson JF, Huang J, Liu KJ, Carlson KR, Foss F, Choi J, Edelson R, Hussong JW, Mohl R, Hill S *et al* (2016) Cutaneous T-cell lymphoma (CTCL): Current practices in blood assessment and the utility of T-cell receptor (TCR)-Vβ chain restriction. *J Am Acad Dermatol* 74: 870-877

Kiel MJ, Sahasrabuddhe AA, Rolland DC, Velusamy T, Chung F, Schaller M, Bailey NG, Betz BL, Miranda RN, Porcu P (2015) Genomic analyses reveal recurrent mutations in epigenetic modifiers and the JAK–STAT pathway in Sézary syndrome. *Nature communications* 6: 8470

Kirsch IR, Watanabe R, O'Malley JT, Williamson DW, Scott LL, Elco CP, Teague JE, Gehad A, Lowry EL, LeBoeuf NR *et al* (2015) TCR sequencing facilitates diagnosis and identifies mature T cells as the cell of origin in CTCL. *Sci Transl Med* 7: 308ra158

Licciulli S, Maksimoska J, Zhou C, Troutman S, Kota S, Liu Q, Duron S, Campbell D, Chernoff J, Field J *et al* (2013) FRAX597, a small molecule inhibitor of the p21-activated kinases, inhibits tumorigenesis of neurofibromatosis type 2 (NF2)-associated Schwannomas. *J Biol Chem* 288: 29105-29114

Lin WM, Lewis JM, Filler RB, Modi BG, Carlson KR, Reddy S, Thornberg A, Saksena G, Umlauf S, Oberholzer PA (2012) Characterization of the DNA copy-number genome in the blood of cutaneous T-cell lymphoma patients. *Journal of investigative dermatology* 132: 188-197

Maj J, Jankowska-Konsur AM, Hałoń A, Woźniak Z, Plomer-Niezgoda E, Reich A (2015) Expression of CXCR4 and CXCL12 and their correlations to the cell proliferation and angiogenesis in mycosis fungoides. *Postepy Dermatol Alergol* 32: 437-442

McGirt LY, Jia P, Baerenwald DA, Duszynski RJ, Dahlman KB, Zic JA, Zwerner JP, Hucks D, Dave U, Zhao Z (2015) Whole-genome sequencing reveals oncogenic mutations in mycosis fungoides. *Blood* 126: 508-519

Netchiporouk E, Litvinov IV, Moreau L, Gilbert M, Sasseville D, Duvic M (2014) Deregulation in STAT signaling is important for cutaneous T-cell lymphoma (CTCL) pathogenesis and cancer progression. *Cell Cycle* 13: 3331-3335

Prasad A, Rabionet R, Espinet B, Zapata L, Puiggros A, Melero C, Puig A, Sarria-Trujillo Y, Ossowski S, Garcia-Muret MP (2016) Identification of gene mutations and fusion genes in patients with Sezary syndrome. *Journal of Investigative Dermatology* 136: 1490-1499

Sun WH, Pabon C, Alsayed Y, Huang PP, Jandeska S, Uddin S, Platanias LC, Rosen ST (1998) Interferon-α Resistance in a Cutaneous T-Cell Lymphoma Cell Line Is Associated With Lack of STAT1 Expression. *Blood* 91: 570-576

van Doorn R, van Kester MS, Dijkman R, Vermeer MH, Mulder AA, Szuhai K, Knijnenburg J, Boer JM, Willemze R, Tensen CP (2009) Oncogenomic analysis of mycosis fungoides reveals major differences with Sezary syndrome. *Blood* 113: 127-136

Wang J, Zhu Y, Chen J, Yang Y, Zhu L, Zhao J, Yang Y, Cai X, Hu C, Rosell R *et al* (2020) Identification of a novel PAK1 inhibitor to treat pancreatic cancer. *Acta Pharmaceutica Sinica B* 10: 603-614

Wang L, Ni X, Covington KR, Yang BY, Shiu J, Zhang X, Xi L, Meng Q, Langridge T, Drummond J (2015) Genomic profiling of Sezary syndrome identifies alterations of key T cell signaling and differentiation genes. *Nature genetics* 47: 1426

Wang Y, Gu X, Li W, Zhang Q, Zhang C (2018) PAK1 overexpression promotes cell proliferation in cutaneous T cell lymphoma via suppression of PUMA and p21. *J Dermatol Sci* 90: 60-67

Wang Y, Li W, Zhang Q, Gu X, He X, Men Y, Zhang C (2019) Targeting Phosphorylation of p21-activated Kinase 1 at Thr423 Induces Cell Cycle Arrest and Apoptosis in Cutaneous T-cell Lymphoma Cells. *Acta Dermato-Venereologica* 99: 1022-1028

Woollard WJ, Pullabhatla V, Lorenc A, Patel VM, Butler RM, Bayega A, Begum N, Bakr F, Dedhia K, Fisher J (2016) Candidate driver genes involved in genome maintenance and DNA repair in Sezary syndrome. *Blood, The Journal of the American Society of Hematology* 127: 3387-3397

Yang SH, Chien CM, Su JC, Chen YL, Chang LS, Lin SR (2008) Novel indoloquinoline derivative, IQDMA, inhibits STAT5 signaling associated with apoptosis in K562 cells. *Journal of biochemical and molecular toxicology* 22: 396-404

2nd Oct 2022

Dear Prof. Moriggl,

Thank you for submitting your revised files. I am pleased to inform you that your manuscript is now accepted for publication in EMBO Molecular Medicine!

There are a few modifications that still need to be done:
- The link provided for the proteomics data seems not to be valid, please check and update. Please also remove the sentence: "All other data associated with this study are presents in the paper or the supplementary materials."
- Thank you for providing the exact p values in Tables EV5 and 6. Please compile them in an appendix table at the end of your appendix, labeled Appendix Table S1. Please update the callouts in the manuscript text.

Once this is done, you may accept all changes and send us the revised manuscript and appendix via email, we will upload them in the system.

Thank you for bearing with these last changes, and congratulations on your work!

With kind regards,

Lise

Lise Roth, Ph.D
Senior Editor
EMBO Molecular Medicine

EMBO Press Author Checklist

Corresponding Author Name: Patrick T. Gunning, Lukas Kenner, Richard Moriggl, Peter Wolf	
Journal Submitted to: EMBO Molecular Medicine	
Manuscript Number: EMM-2021-15200	

USEFUL LINKS FOR COMPLETING THIS FORM
[The EMBO Journal - Author Guidelines]
[EMBO Reports - Author Guidelines]
[Molecular Systems Biology - Author Guidelines]
[EMBO Molecular Medicine - Author Guidelines]

Reporting Checklist for Life Science Articles (updated January 2022)
This checklist is adapted from Materials Design Analysis Reporting (MDAR) Checklist for Authors. MDAR establishes a minimum set of requirements in transparent reporting in the life sciences (see Statement of Task: 10.31222/osf.io/9sm4x). Please follow the journal's guidelines in preparing your manuscript.
Please note that a copy of this checklist will be published alongside your article.

Abridged guidelines for figures
1. Data
The data shown in figures should satisfy the following conditions:
- the data were obtained and processed according to the field's best practice and are presented to reflect the results of the experiments in an accurate and unbiased manner.
- ideally, figure panels should include only measurements that are directly comparable to each other and obtained with the same assay.
- plots include clearly labeled error bars for independent experiments and sample sizes. Unless justified, error bars should not be shown for technical replicates.
- if n<5, the individual data points from each experiment should be plotted. Any statistical test employed should be justified.
- Source Data should be included to report the data underlying figures according to the guidelines set out in the authorship guidelines on Data Presentation.

2. Captions
Each figure caption should contain the following information, for each panel where they are relevant:
- a specification of the experimental system investigated (eg cell line, species name).
- the assay(s) and method(s) used to carry out the reported observations and measurements.
- an explicit mention of the biological and chemical entity(ies) that are being measured.
- an explicit mention of the biological and chemical entity(ies) that are altered/varied/perturbed in a controlled manner.
- the exact sample size (n) for each experimental group/condition, given as a number, not a range;
- a description of the sample collection allowing the reader to understand whether the samples represent technical or biological replicates (including how many animals, litters, cultures, etc.).
- a statement of how many times the experiment shown was independently replicated in the laboratory.
- definitions of statistical methods and measures:
  - common tests, such as t-test (please specify whether paired vs. unpaired), simple χ2 tests, Wilcoxon and Mann-Whitney tests, can be unambiguously identified by name only, but more complex techniques should be described in the methods section;
  - are tests one-sided or two-sided?
  - are there adjustments for multiple comparisons?
  - exact statistical test results, e.g., P values = x but not P values < x;
  - definition of 'center values' as median or average;
  - definition of error bars as s.d. or s.e.m.

Please complete ALL of the questions below.
Select "Not Applicable" only when the requested information is not relevant for your study.

Materials

Newly Created Materials	Information included in the manuscript?	In which section is the information available? (Reagents and Tools Table, Materials and Methods, Figures, Data Availability Section)
New materials and reagents need to be available; do any restrictions apply?	Yes	The small molecule inhibitors/drugs used in this study are either available in the market, whose details can be found in the appendix data set 'drugs used,' or they were already published, and we correctly cited them in the reference

Antibodies	Information included in the manuscript?	In which section is the information available? (Reagents and Tools Table, Materials and Methods, Figures, Data Availability Section)
For **antibodies** provide the following information:<br>- Commercial antibodies: RRID (if possible) or supplier name, catalogue number and or/clone number<br>- Non-commercial: RRID or citation	Yes	The antibodies used in this study are commercially available in the market; their details, catalog number, etc., are disclosed in the appendix section data set 'antibodies.'

DNA and RNA sequences	Information included in the manuscript?	In which section is the information available? (Reagents and Tools Table, Materials and Methods, Figures, Data Availability Section)
**Short novel DNA or RNA including primers, probes**: provide the sequences.	Yes	All the primers used in this study, their gene ID, species name, amplicon size, primer sequence (5' -> 3'), length, location, efficiency, R^2, and slope, are reported in the appendix dataset; under 'Primers.'

Cell materials	Information included in the manuscript?	In which section is the information available? (Reagents and Tools Table, Materials and Methods, Figures, Data Availability Section)
**Cell lines:** Provide species information, strain. Provide accession number in repository **OR** supplier name, catalog number, clone number, and/**OR** RRID.	Yes	All the cell lines used in this study are described/discussed in the Supplementary Materials and Methods section.
**Primary cultures:** Provide species, strain, sex of origin, genetic modification status.	Yes	Primary patient cells used in this study were described /discussed in the Results section, in the materials & method section under 'human samples', and in the appendix/supplementary table 1 (L-CTCL Patient clinical data
Report if the cell lines were recently **authenticated** (e.g., by STR profiling) and tested for mycoplasma contamination.	Yes	All cell lines are STR profiled and routinely checked for mycoplasma contamination. STR profiling reports are attached in appendix data "STR profiling."

Experimental animals	Information included in the manuscript?	In which section is the information available? (Reagents and Tools Table, Materials and Methods, Figures, Data Availability Section)
**Laboratory animals or Model organisms:** Provide species, strain, sex, age, genetic modification status. Provide accession number in repository **OR** supplier name, catalog number, clone number, **OR** RRID.	Yes	Laboratory animals used in this study are described in the materials and method section of the animal experiment.
**Animal observed in or captured from the field:** Provide species, sex, and age where possible.	Not Applicable	
Please detail **housing and husbandry conditions**.	Yes	Described in Materials and Methods

Plants and microbes	Information included in the manuscript?	In which section is the information available? (Reagents and Tools Table, Materials and Methods, Figures, Data Availability Section)
**Plants:** provide species and strain, ecotype and cultivar where relevant, unique accession number if available, and source (including location for collected wild specimens).	Not Applicable	
**Microbes:** provide species and strain, unique accession number if available, and source.	Not Applicable	

Human research participants	Information included in the manuscript?	In which section is the information available? (Reagents and Tools Table, Materials and Methods, Figures, Data Availability Section)
If collected and within the bounds of privacy constraints report on age, sex and gender or ethnicity for all study participants.	Yes	Primary patient data used in this study were described in the appendix/supplementary table 1 under "L-CTCL Patient clinical data table."

Core facilities	Information included in the manuscript?	In which section is the information available? (Reagents and Tools Table, Materials and Methods, Figures, Data Availability Section)
If your work benefited from core facilities, was their service mentioned in the acknowledgments section?	Yes	Core facility service personnel are included in the author list or acknowledgment list.

Design

Study protocol	Information included in the manuscript?	In which section is the information available? (Reagents and Tools Table, Materials and Methods, Figures, Data Availability Section)
If study protocol has been **pre-registered, provide DOI in the manuscript**. For clinical trials, provide the trial registration number **OR** cite DOI.	Not Applicable	
Report the **clinical trial registration number** (at ClinicalTrials.gov or equivalent), where applicable.	Not Applicable	

Laboratory protocol	Information included in the manuscript?	In which section is the information available? (Reagents and Tools Table, Materials and Methods, Figures, Data Availability Section)
Provide DOI OR other citation details if **external detailed step-by-step protocols** are available.	Not Applicable	

Experimental study design and statistics	Information included in the manuscript?	In which section is the information available? (Reagents and Tools Table, Materials and Methods, Figures, Data Availability Section)
Include a statement about **sample size** estimate even if no statistical methods were used.	Yes	Pilot studies were conducted, and tumor growth curves were estimated for all three different graft models in NSG mice. Based on the pilot study, N=6 tumor grafts were considered a good sample size for carrying on with further
Were any steps taken to minimize the effects of subjective bias when allocating animals/samples to treatment (e.g. **randomization procedure**)? If yes, have they been described?	Yes	After successful engraftment of the tumor, mice were randomized before dividing into the vehicle and different treatment groups.
Include a statement about **blinding** even if no blinding was done.	Not Applicable	
Describe **inclusion/exclusion criteria** if samples or animals were excluded from the analysis. Were the criteria pre-established?<br><br>If sample or data points were omitted from analysis, report if this was due to attrition or intentional exclusion and provide justification.	Yes	Patients with clinically diagnosed Sezary syndrome or leukemic advanced MF Mycosis fungoides patients with a prominent clone in the blood were the initial criteria for selection in this study.<br>For whole-genome sequencing, quality criteria were ≥80% of bases to be ≥Q30. For animal experimentation: only successfully grafted mice were randomized into different groups before starting the treatment
For every figure, are **statistical tests** justified as appropriate? Do the data meet the assumptions of the tests (e.g., normal distribution)? Describe any methods used to assess it. Is there an estimate of variation within each group of data? Is the variance similar between the groups that are being statistically compared?	Yes	Welch's corrections were used for all the unpaired T-tests to account for unequal SD between the groups.

Sample definition and in-laboratory replication	Information included in the manuscript?	In which section is the information available? (Reagents and Tools Table, Materials and Methods, Figures, Data Availability Section)
In the figure legends: state number of times the experiment was **replicated** in laboratory.	Yes	Where applicable, the number of technical/biological replicates (n) is mentioned in figure legends.
In the figure legends: define whether data describe **technical or biological replicates**.	Yes	Where applicable, the number of technical/biological replicates (n) is mentioned in figure legends.

Ethics

Ethics	Information included in the manuscript?	In which section is the information available? (Reagents and Tools Table, Materials and Methods, Figures, Data Availability Section)
Studies involving **human participants**: State details of **authority granting ethics approval** (IRB or equivalent committee(s), provide reference number for approval.	Yes	Ethical approval numbers, granting authority, etc., are described in the materials & methods section of the manuscript under the human samples.
Studies involving **human participants**: Include a statement confirming that **informed consent** was obtained from all subjects and that the experiments conformed to the principles set out in the WMA Declaration of Helsinki and the Department of Health and Human Services Belmont Report.	Yes	The study was conducted in accordance with the Declaration of Helsinki, and its included in the materials & methods section of the manuscript under the human samples.
Studies involving **human participants:** For publication of **patient photos**, include a statement confirming that consent to publish was obtained.	Not Applicable	
Studies involving experimental **animals**: State details of **authority granting ethics approval** (IRB or equivalent committee(s), provide reference number for approval. Include a statement of compliance with ethical regulations.	Yes	IRB approval number and granting authority details are included in the materials & methods section of the manuscript under the mouse experiments section.
Studies involving **specimen and field samples:** State if relevant **permits** obtained, provide details of authority approving study; if none were required, explain why.	Not Applicable	

Dual Use Research of Concern (DURC)	Information included in the manuscript?	In which section is the information available? (Reagents and Tools Table, Materials and Methods, Figures, Data Availability Section)
Could your study fall under dual use research restrictions? Please check biosecurity documents and list of **select agents and toxins** (CDC): https://www.selectagents.gov/sat/list.htm	Not Applicable	
If you used a select agent, is the security level of the lab appropriate and reported in the manuscript?	Not Applicable	
If a study is subject to dual use research of concern regulations, is the name of the **authority granting approval and reference number** for the regulatory approval provided in the manuscript?	Not Applicable	

Reporting

The MDAR framework recommends adoption of discipline-specific guidelines, established and endorsed through community initiatives. Journals have their own policy about requiring specific guidelines and recommendations to complement MDAR.

Adherence to community standards	Information included in the manuscript?	In which section is the information available? (Reagents and Tools Table, Materials and Methods, Figures, Data Availability Section)
State if relevant guidelines or checklists (e.g., **ICMJE, MIBBI, ARRIVE, PRISMA**) have been followed or provided.	Not Applicable	
For **tumor marker prognostic studies,** we recommend that you follow the **REMARK** reporting guidelines (see link list at top right). See author guidelines, under 'Reporting Guidelines'. Please confirm you have followed these guidelines.	Not Applicable	
For **phase II and III randomized controlled trials**, please refer to the **CONSORT** flow diagram (see link list at top right) and submit the CONSORT checklist (see link list at top right) with your submission. See author guidelines, under 'Reporting Guidelines'. Please confirm you have submitted this list.	Not Applicable	

Data Availability

Data availability	Information included in the manuscript?	In which section is the information available? (Reagents and Tools Table, Materials and Methods, Figures, Data Availability Section)
Have **primary datasets** been deposited according to the journal's guidelines (see 'Data Deposition' section) and the respective accession numbers provided in the Data Availability Section?	Yes	The raw sequencing data (WES and sWGS) are available from the European Genome-phenome archive (EGA) database under the accession code is mentioned in the "Data and materials availability" section. All other data associated with this study are presents in the paper or the supplementary
Were **human clinical and genomic datasets** deposited in a public access-controlled repository in accordance to ethical obligations to the patients and to the applicable consent agreement?	Yes	Yes, the human clinical and genomic datasets were collected and deposited in the presence of original informed consent agreements signed by the participants involved in our study.
Are **computational models** that are central and integral to a study available without restrictions in a machine-readable form? Were the relevant accession numbers or links provided?	Not Applicable	
If publicly available data were reused, provide the respective **data citations in the reference list**.	Yes	Expression data were downloaded and from the Oncomine database, and corresponding study was cited in the text.
